# Supplementary material for: Functional rescue of a disease-linked ERAD pathway mutation via alternative splicing
Source: EMBO J. 2026 Mar 20;45(9):3230–51. doi: 10.1038/s44318-026-00757-5 (PMC13144729; doi:10.1038/s44318-026-00757-5)
Supplement: Supplementary file 1 — Appendix [file 44318_2026_757_MOESM1_ESM.pdf]

Appendix for

Functional rescue of a disease-linked ERAD pathway mutation via alternative splicing

Appendix Figure S1

Appendix Table S1

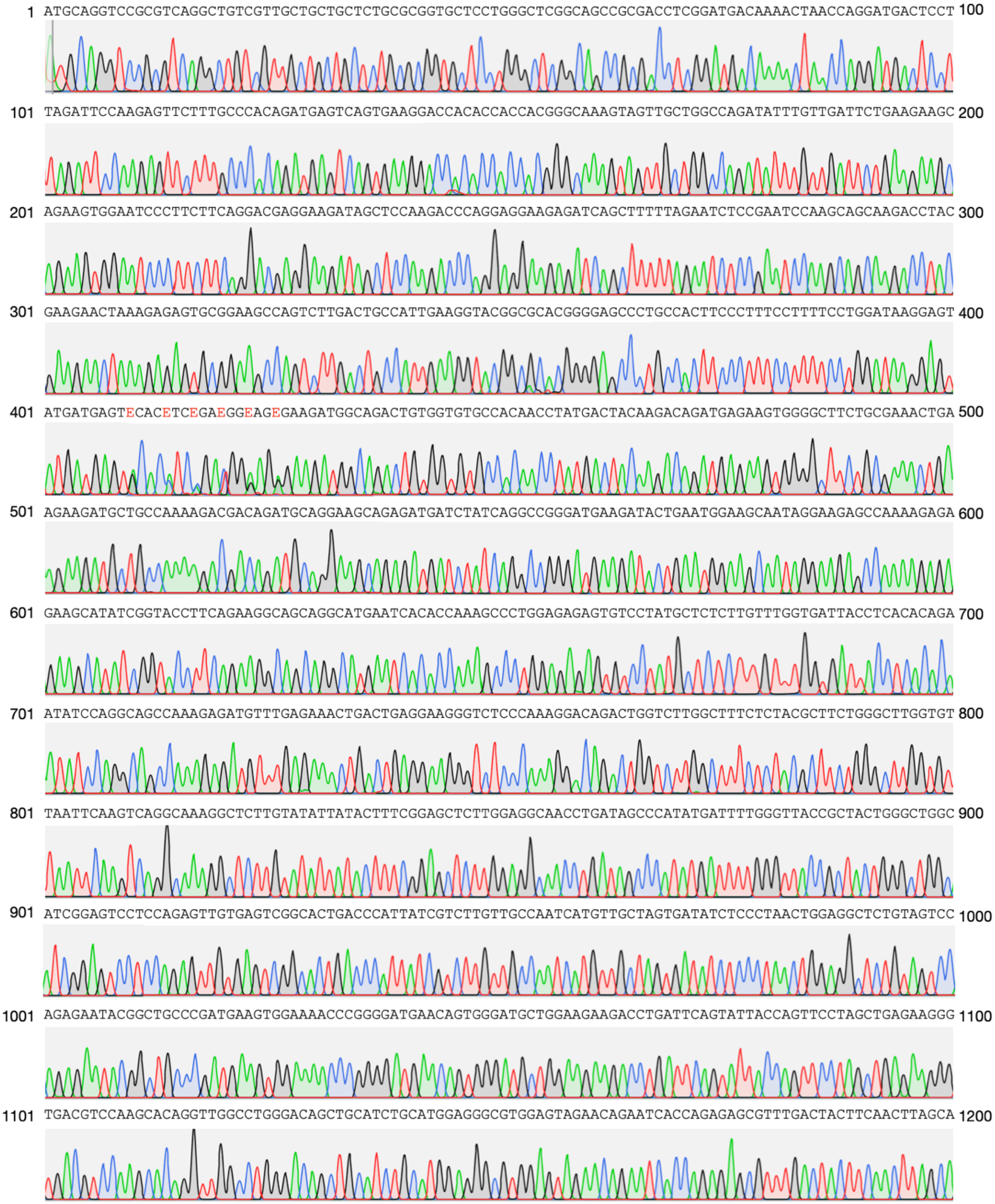
1 ATGCAGGTCCGCGTCAGGCTGTCGTTGCTGCTGCTCTGCGCGGTGCTCCTGGGCTCGGCAGCCGCGACCTCGGATGACAAAACTAACCAGGATGACTCCT 100
101 TAGATTCCAAGAGTTCTTTGCCCACAGATGAGTCAGTGAAGGACCACACCACCACGGGCAAAGTAGTTGCTGGCCAGATATTTGTTGATTCTGAAGAAGC 200
201 AGAAGTGGAATCCCTTCTTCAGGACGAGGAAGATAGCTCCAAGACCCAGGAGGAAGAGATCAGCTTTTTAGAATCTCCGAATCCAAGCAGCAAGACCTAC 300
301 GAAGAACTAAAGAGAGTGCGGAAGCCAGTCTTGACTGCCATTGAAGGTACGGCGCACGGGGAGCCCTGCCACTTCCCTTTCCTTTTCCTGGATAAGGAGT 400
401 ATGATGAGTECACETCEGAEGGEAGEGAAGATGGCAGACTGTGGTGTGCCACAACCTATGACTACAAGACAGATGAGAAGTGGGGCTTCTGCGAAACTGA 500
501 AGAAGATGCTGCCAAAAGACGACAGATGCAGGAAGCAGAGATGATCTATCAGGCCGGGATGAAGATACTGAATGGAAGCAATAGGAAGAGCCAAAAGAGA 600
601 GAAGCATATCGGTACCTTCAGAAGGCAGCAGGCATGAATCACACCAAAGCCCTGGAGAGAGTGTCCTATGCTCTCTTGTTTGGTGATTACCTCACACAGA 700
701 ATATCCAGGCAGCCAAAGAGATGTTTGAGAAACTGACTGAGGAAGGGTCTCCCAAAGGACAGACTGGTCTTGGCTTTCTCTACGCTTCTGGGCTTGGTGT 800
801 TAATTCAAGTCAGGCAAAGGCTCTTGTATATTATACTTTCGGAGCTCTTGGAGGCAACCTGATAGCCCATATGATTTTGGGTTACCGCTACTGGGCTGGC 900
901 ATCGGAGTCCTCCAGAGTTGTGAGTCGGCACTGACCCATTATCGTCTTGTTGCCAATCATGTTGCTAGTGATATCTCCCTAACTGGAGGCTCTGTAGTCC 1000
1001 AGAGAATACGGCTGCCCGATGAAGTGGAAAACCCCGGGGATGAACAGTGGGATGCTGGAAGAAGACCTGATTCAGTATTACCAGTTCCTAGCTGAGAAGGG 1100
1101 TGACGTCCAAGCACAGGTTGGCCTGGGACAGCTGCATCTGCATGGAGGGCGTGGAGTAGAACAGAATCACCAGAGAGCGTTTGACTACTTCAACTTAGCA 1200

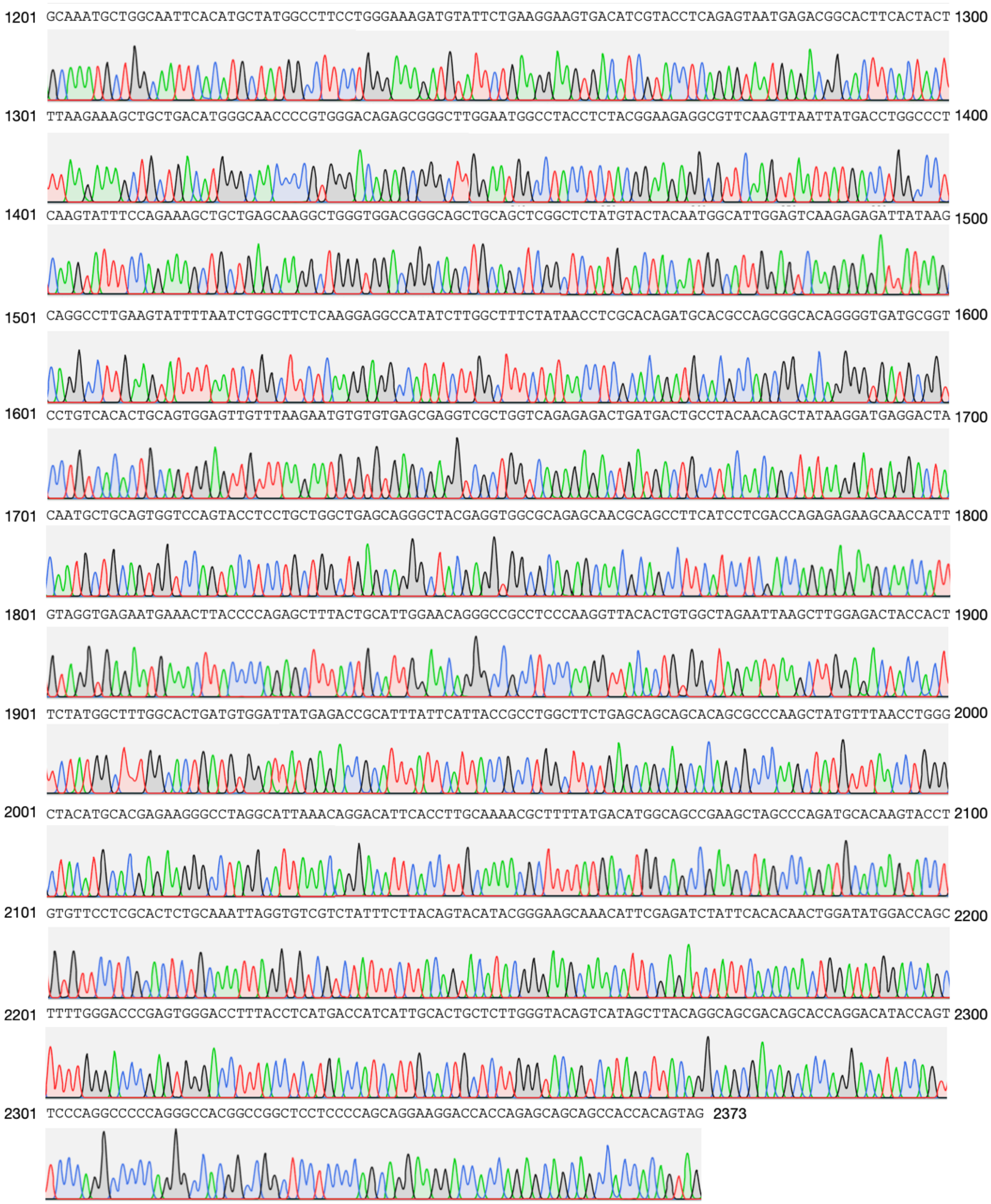

Appendix Figure S1. The Sanger sequencing confirmation of *Sel1L* C141Y Heterozygous mouse coding DNA sequence. Sanger sequencing confirmation of the *Sel1L* C141Y heterozygous coding DNA sequence in mouse liver, obtained from multiple Sanger sequencing fragments. E, heterozygous allele.

Appendix Table S1. The primers and ASOs used in the study

Name	Sequence
Genotyping primer for *Sel1L*^{C141Y} allele	F 5’- AGTACACATCCGATGGAAGAGAAG-3’;<br>R 5’-GAAAATGCCTTCCAAATGCTGC-3’;
Genotyping primer for *Sel1L* wildtype allele	F 5’- AGTGCACCTCAGACGGGAGGG-3’;<br>R 5’- GAAAATGCCTTCCAAATGCTGC-3’;
Sequencing primer for *mSel1L* genomic DNA Exon 4 and intron 4	F 5’- CTTAAGAACTCAAAGTCTACACTAAGTCT-3’;<br>R 5’- CAGCTTGCCTCAAGGGTTTACAGAA-3’;
Mutagenesis primers for alternative splicing donor site (Mutation 1)	F 5’-CCATTGAAGGCACGGCGCACGGGGA-3’<br>R 5’-GCGCCGTGCCTTCAATGGCAGTCAAGA-3’
Mutagenesis primers for Exon 4 canonical splicing donor site mutation (Mutation 2)	F 5’-CTTCTGCGAAAGGCGTATTGTTCAAGTGGGG-3’<br>R 5’-AACAATACGCCTTTCGCAGAAGCCCCACTTCT-3’
Mutagenesis primers for Exon 4 C137Y mutation (Mutation 3)	F 5’- GTATGATGAGTACACCTCAGACG-3’<br>R 5’- CGTCTGAGGTGTACTCATCATAC-3’
Mutagenesis primers for Exon 4 synonymous mutations (Mutation 4)	F 5’- GATGAGTGCACATCCGATGGAAGAGAAGATGG-CAGACTGTGG-3’<br>R 5’- GCCATCTTCTCTTCCATCGGATGTGCACTCATCAT-ACTCC-3’
mSEL1L Full length (F1/R1)	F 5’- ATGCAGGTCCGCGTCAGGCTGTCGTTGCTGCT-3’;<br>R 5’- CTACTGTGGTGGCTGCTGCTCTGG-3’;
hSEL1L Full length (F1’/R1’):	F 5’- GCGGCTAGCATGCGGGTCCGGATAGGGCT-3’;<br>R 5’- GCGAAGCTTTTACTGTGGTGGCTGCTGCTCTG-3’;
mSEL1L Exon4 for acrylamide gel (F2/R2)	F 5’- AGCAAGACCTACGAAGAACT-3’;<br>R 5’- GAAGGTACCGATATGCTTCTCTCT-3’;
mSEL1L Exon4 for agarose gel (F3/R3)	F 5’- ATGCAGGTCCGCGTCAGGCTGTCGTTGCTGCT -3’;<br>R 5’- CTACTGTGGTGGCTGCTGCTCTGG -3’;
Minigene Exon4 (F4/R4)	F 5’- TGGTGAGCAAGGGCGAGG-3’;<br>R 5’- CGTCCTTGAAGAAGATGGTGCG-3’;
hSEL1L Exon4 (F5/R5)	F 5’- GGGGAAAGTGTCACAGAAGATATCAG-3’;<br>R 5’- GACACTCTCTCCAGGGCTTTG-3’;

mXbp1s	F 5’- ACGAGGTTCCAGAGGTGGAG-3’;<br>R 5’- AAGAGGCAACAGTGTCAGAG-3’;
hXBP1s	F 5’- GAATGAAGTGAGGCCAGTGG-3’;<br>R 5’- ACTGGGTCCTTCTGGGTAGA-3’;
mL32	F 5’- GAGCAACAAGAAAACCAAGCA-3’;<br>R 5’- TGCACACAAGCCATCTACTCA-3’;
hPPIA	F 5’- GGCAAATGCTGGACCCAACACA-3’;<br>R 5’- TGCTGGTCTTGCCATTCCTGGA-3’
Standard control oligos	CCTCTTACCTCAGTTACAATTTATA
ASO1 (+167bp - +192bp)	TGCCTCCTACTGAGCAATACTTACT
ASO2 (+137bp - +162bp)	AAAAGCCCCACTTTTCATCTGCTTT
ASO3 (+107bp - +132bp)	CATAGGTTGTAGCACACCACAGTCT
ASO4 (+77bp - +102bp)	CTTCCCTCCCATCTGATGTATATTC
ASO5 (+47bp - +86bp)	ACTCCTTATCTAGGAAAAGAAAAGG
ASO6 (+17bp - +42bp)	GGCAGGGCTCCCCATGTGCTGTGCC
ASO7 (-14bp - +11bp)	GGCGGTCAAAGCTGGAATGACAAGA
