# Supplementary figures and images for: Functional rescue of a disease-linked ERAD pathway mutation via alternative splicing
Source: EMBO J. 2026 Mar 20;45(9):3230–51. doi: 10.1038/s44318-026-00757-5 (PMC13144729; doi:10.1038/s44318-026-00757-5)

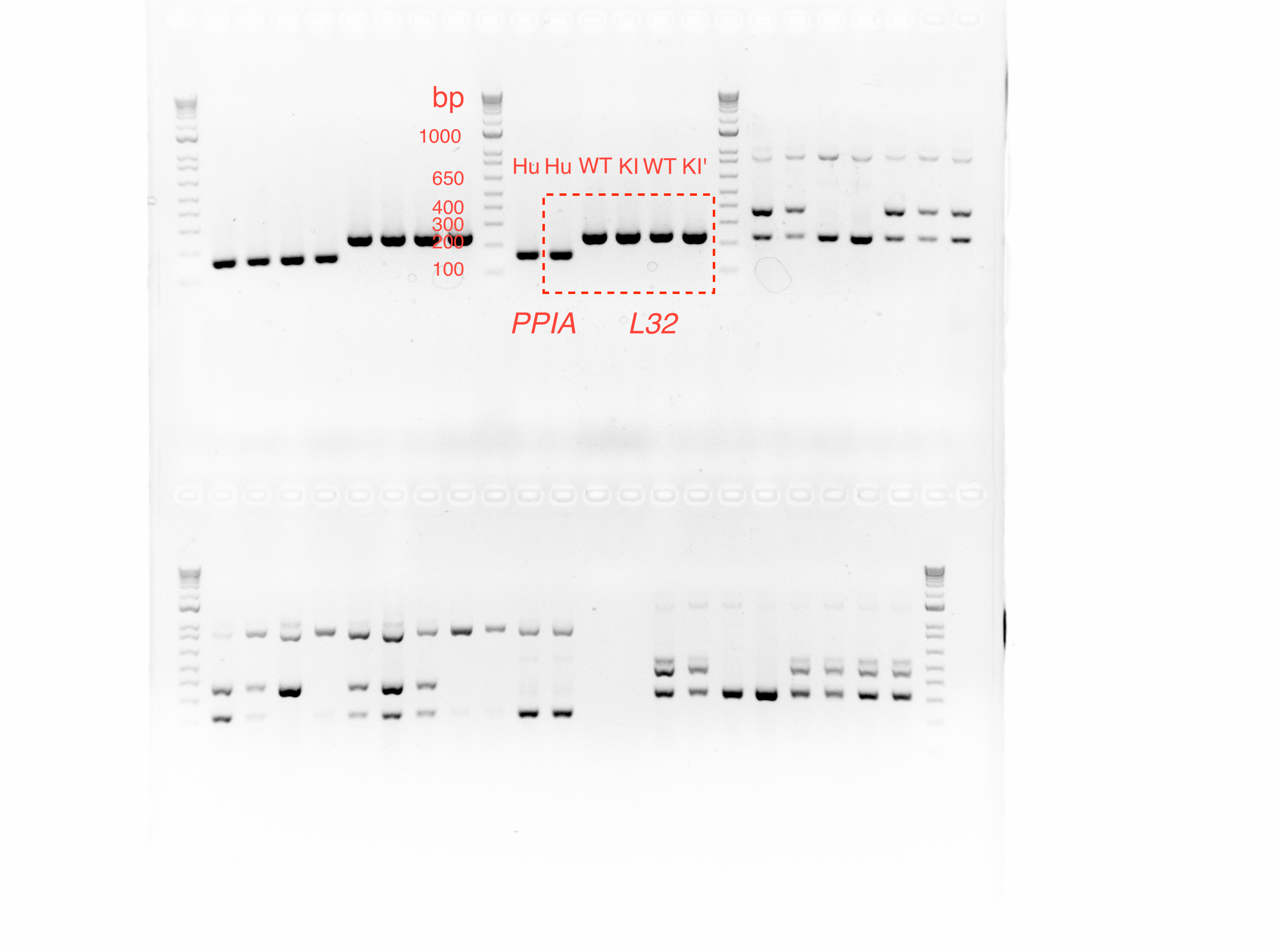

Supplement: Supplementary file 3 — Source data Fig. 1 [file 44318_2026_757_MOESM3_ESM.zip › Figure 1/Figure 1F/Agarose gel PPIA and L32.tif]

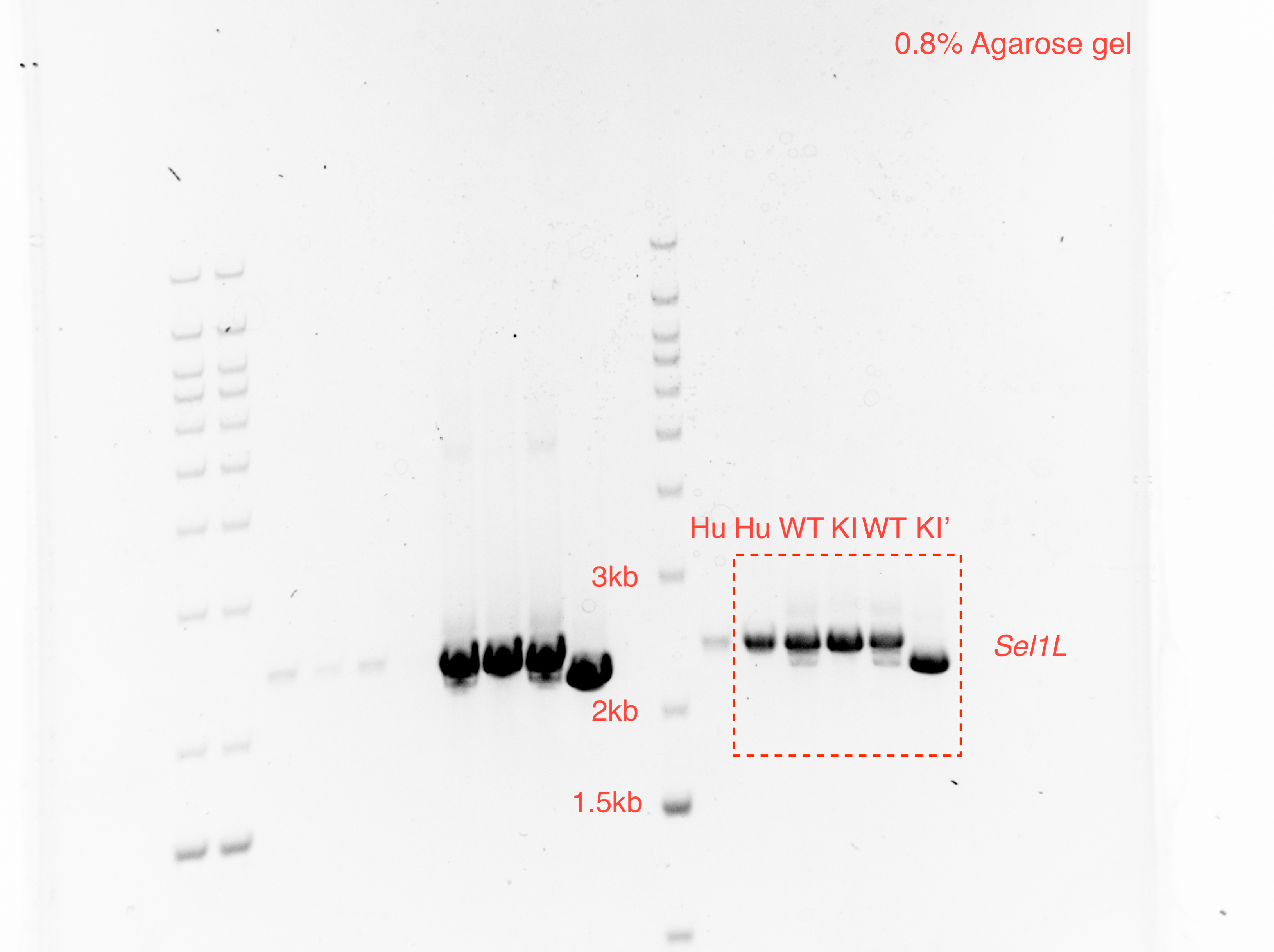

Supplement: Supplementary file 3 — Source data Fig. 1 [file 44318_2026_757_MOESM3_ESM.zip › Figure 1/Figure 1F/Agarose gel SEL1L Full length.tif]

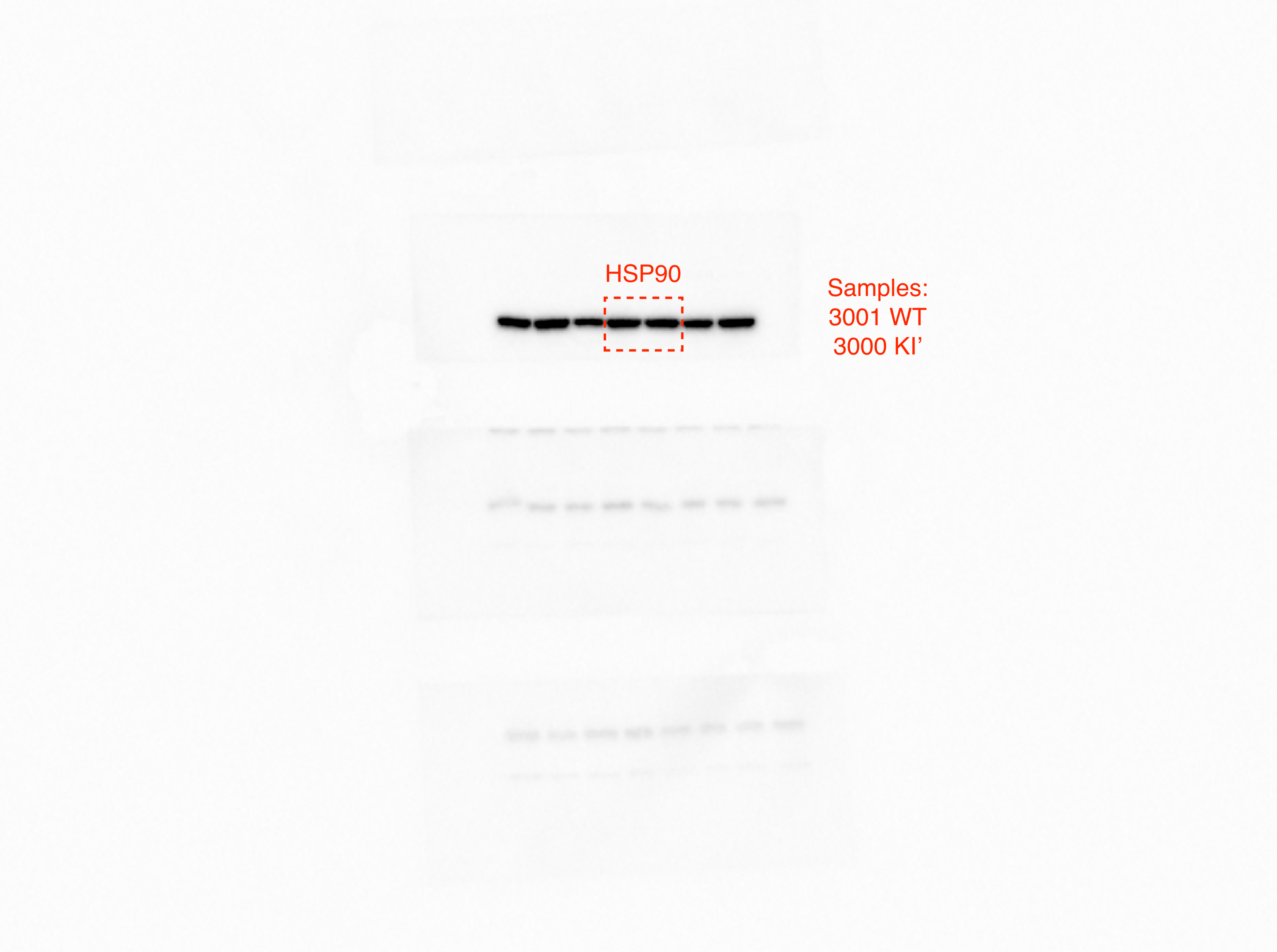

Supplement: Supplementary file 4 — Source data Fig. 2 [file 44318_2026_757_MOESM4_ESM.zip › Figure 2/Figure 2G/IP HSP90.tif]

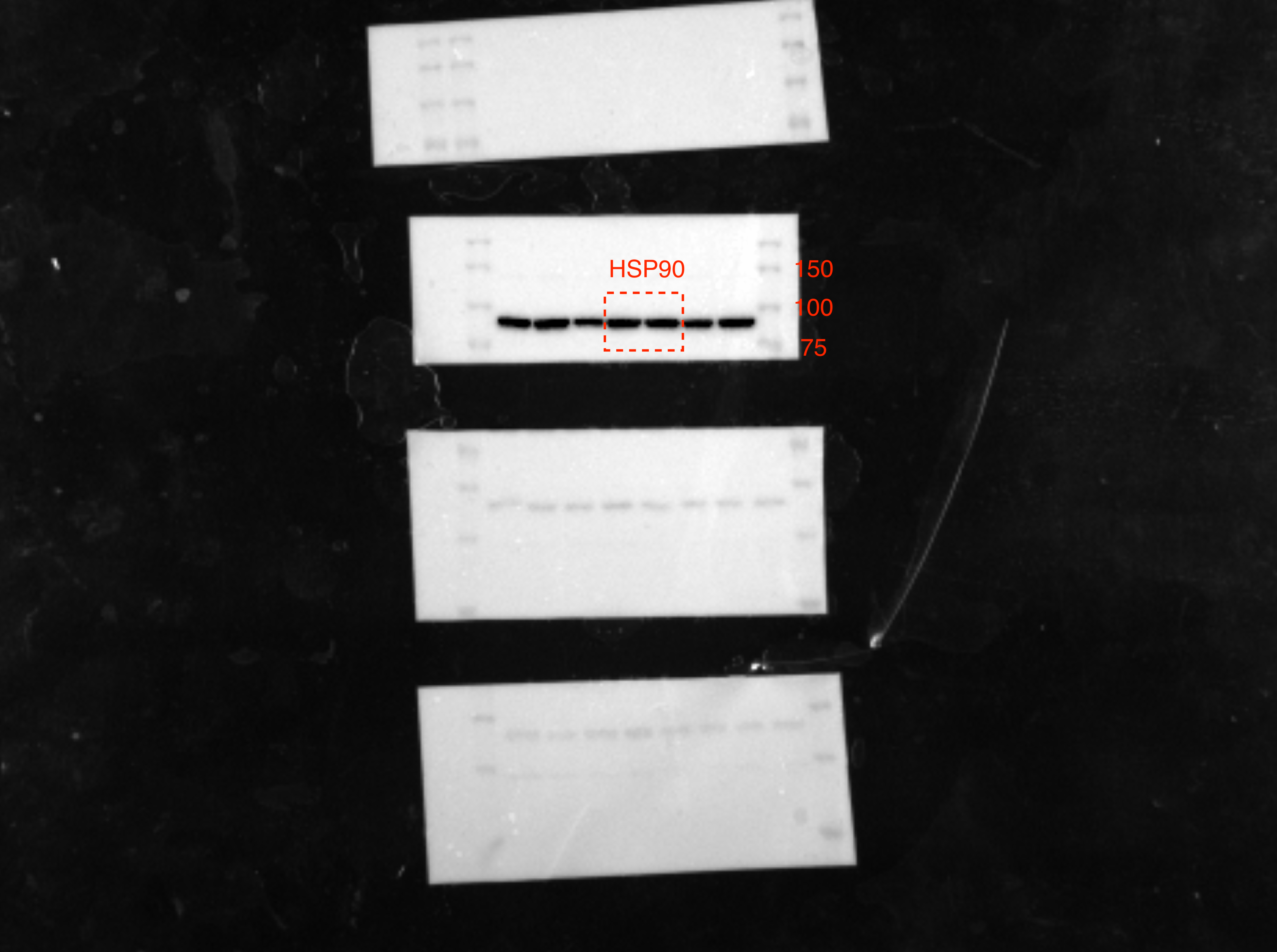

Supplement: Supplementary file 4 — Source data Fig. 2 [file 44318_2026_757_MOESM4_ESM.zip › Figure 2/Figure 2G/IP HSP90 merged with marker.tif]

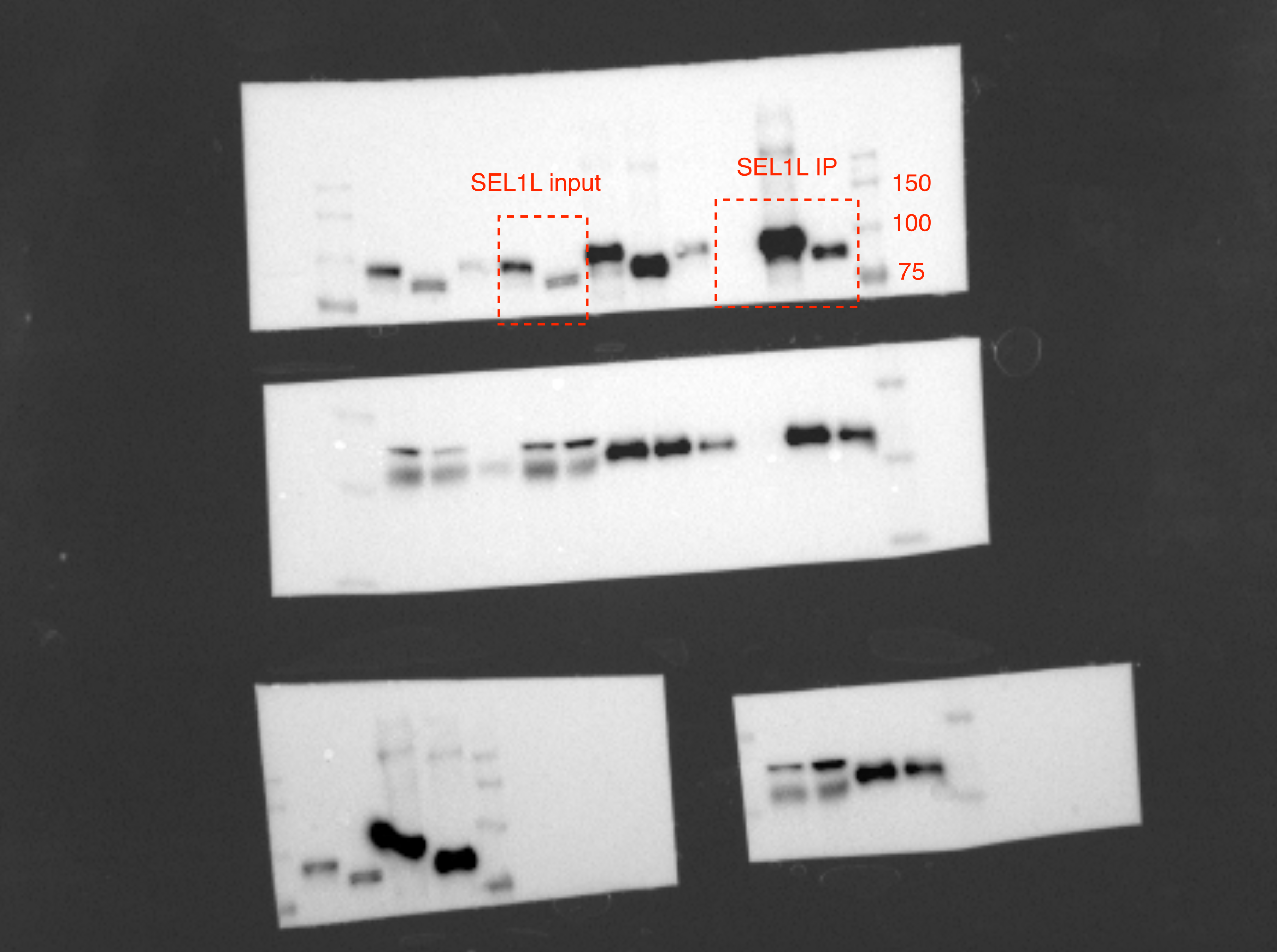

Supplement: Supplementary file 4 — Source data Fig. 2 [file 44318_2026_757_MOESM4_ESM.zip › Figure 2/Figure 2G/IP SEL1L merged with marker.tif]

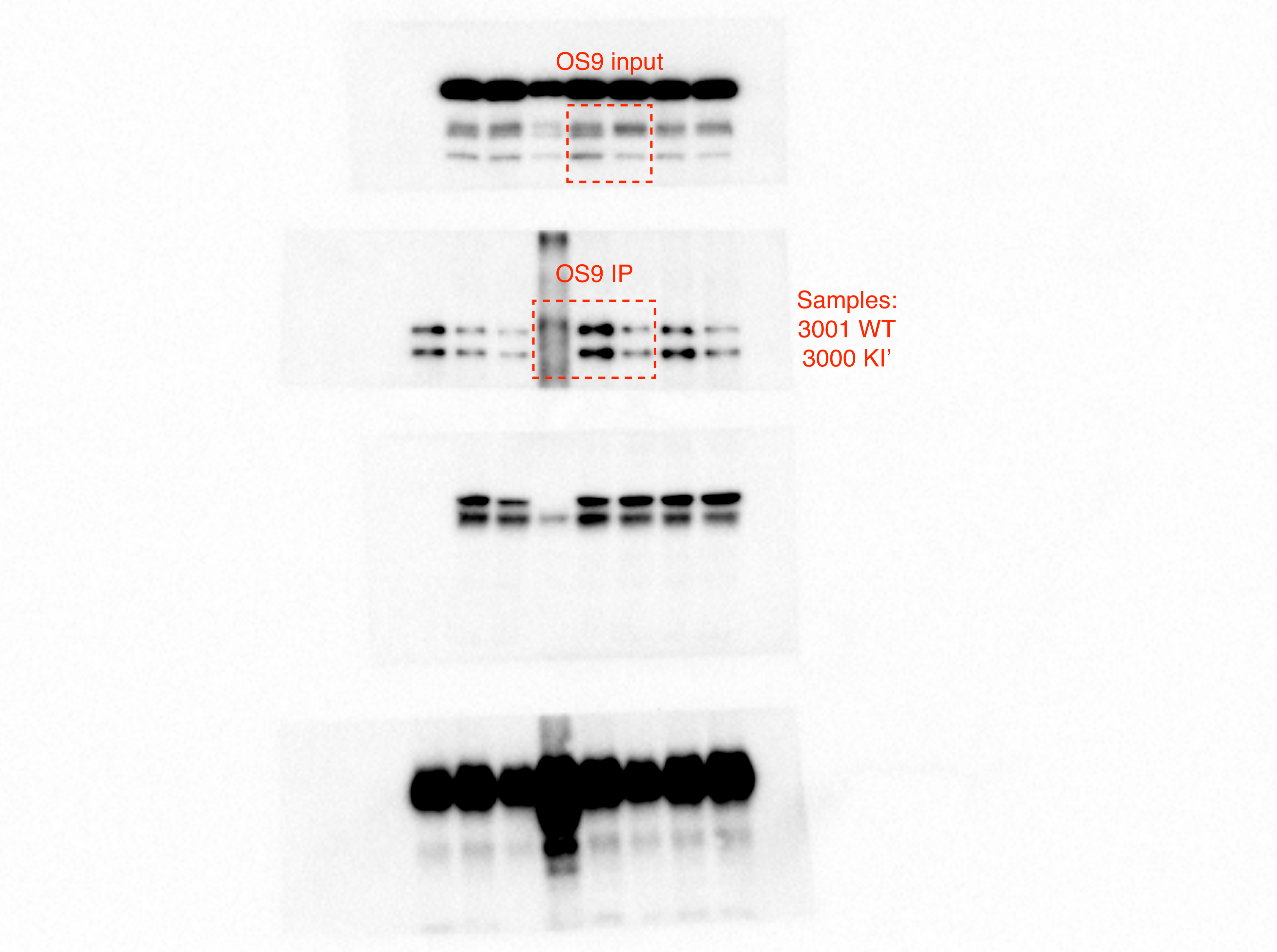

Supplement: Supplementary file 4 — Source data Fig. 2 [file 44318_2026_757_MOESM4_ESM.zip › Figure 2/Figure 2G/IP OS9.tif]

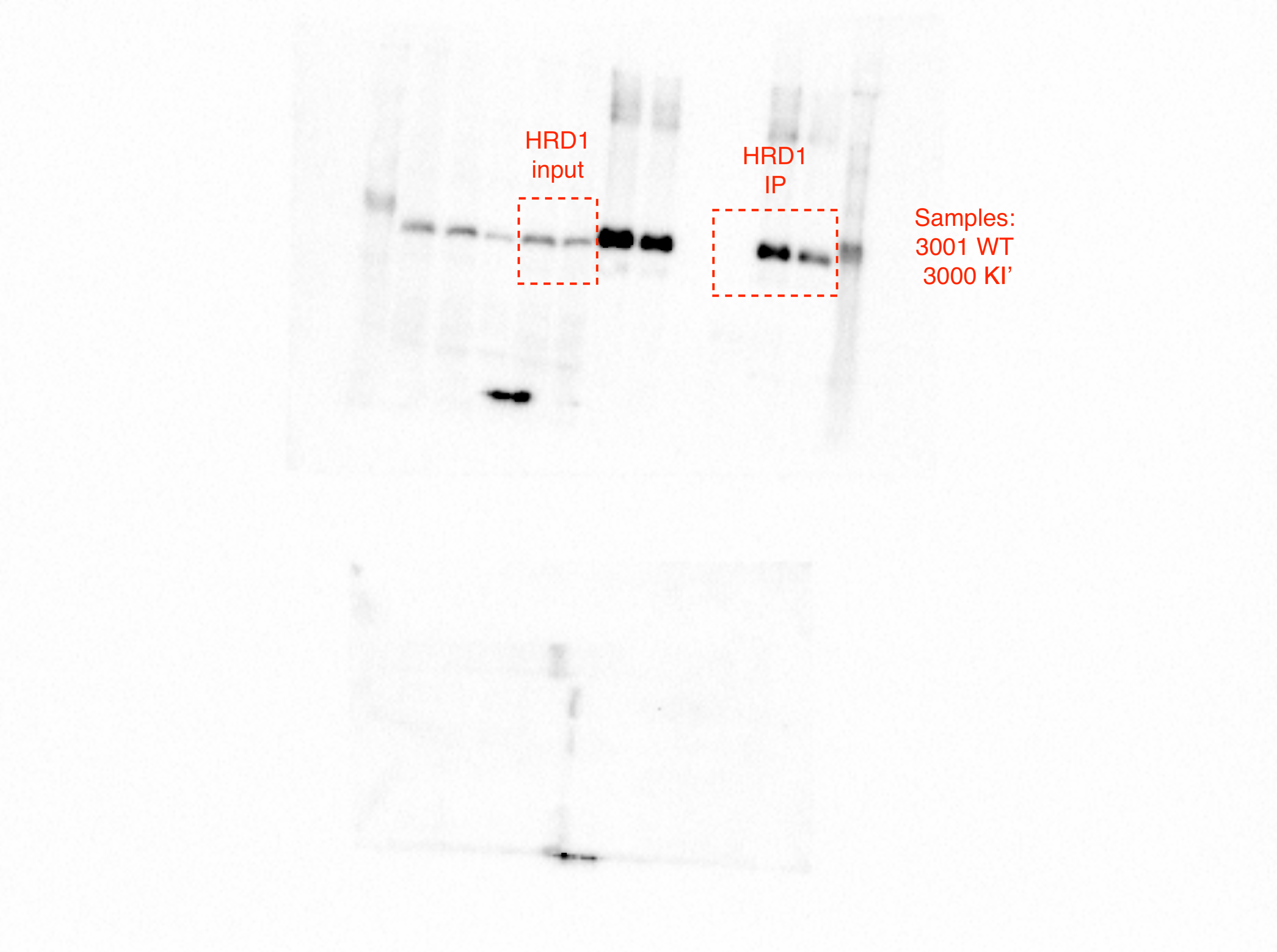

Supplement: Supplementary file 4 — Source data Fig. 2 [file 44318_2026_757_MOESM4_ESM.zip › Figure 2/Figure 2G/IP HRD1 no marker.tif]

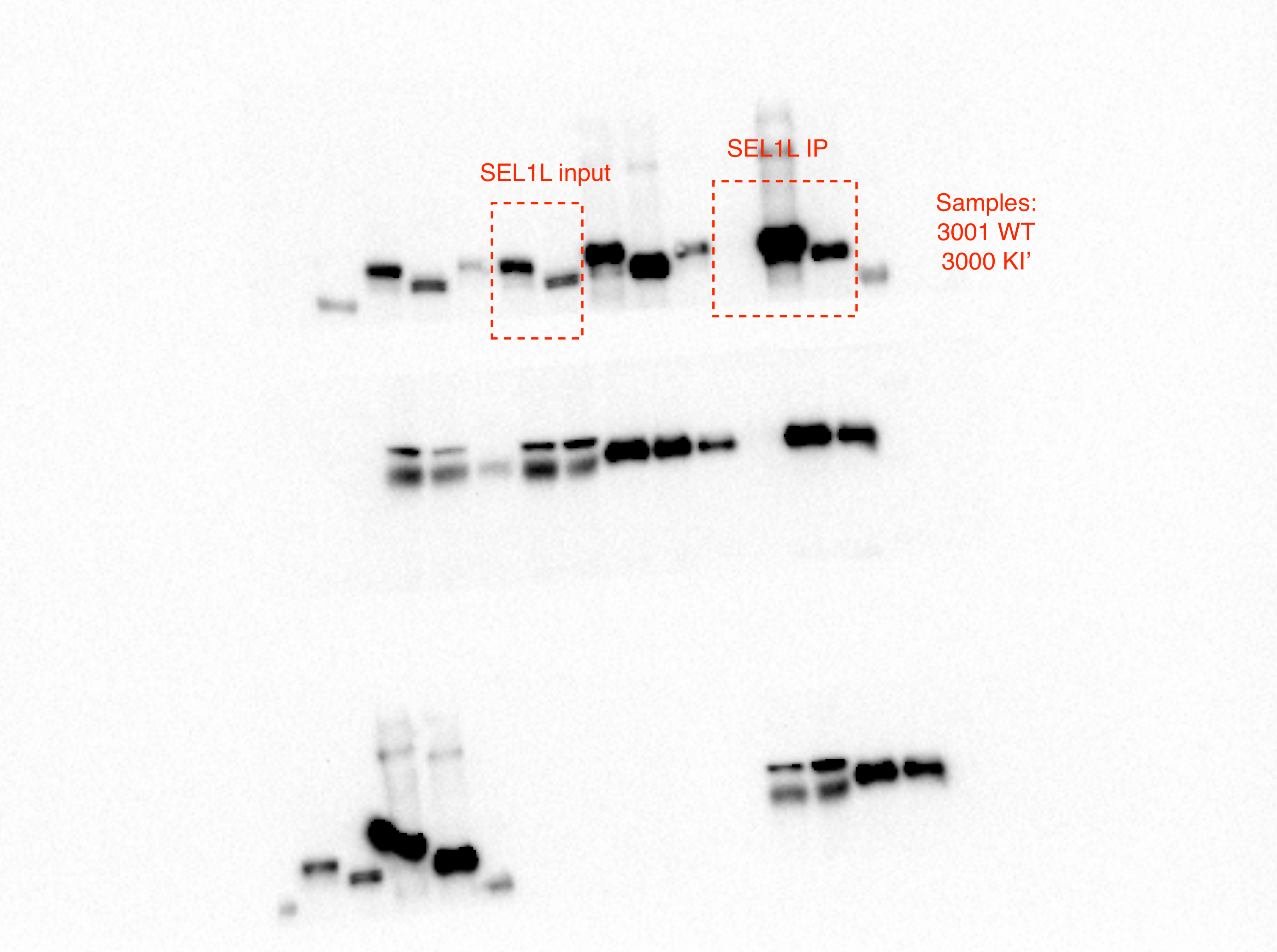

Supplement: Supplementary file 4 — Source data Fig. 2 [file 44318_2026_757_MOESM4_ESM.zip › Figure 2/Figure 2G/IP SEL1L.tif]

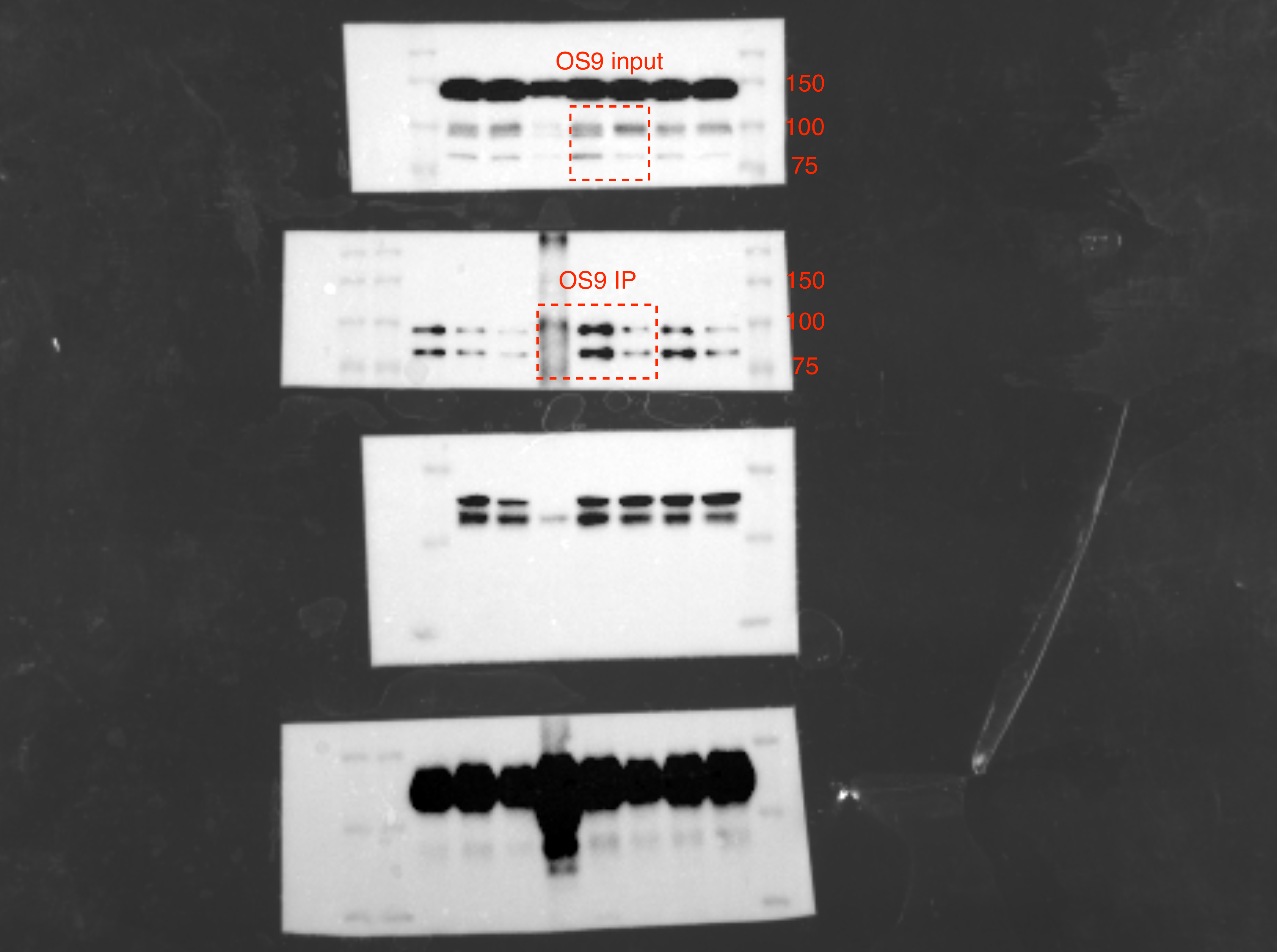

Supplement: Supplementary file 4 — Source data Fig. 2 [file 44318_2026_757_MOESM4_ESM.zip › Figure 2/Figure 2G/IP OS9 merged with marker.tif]

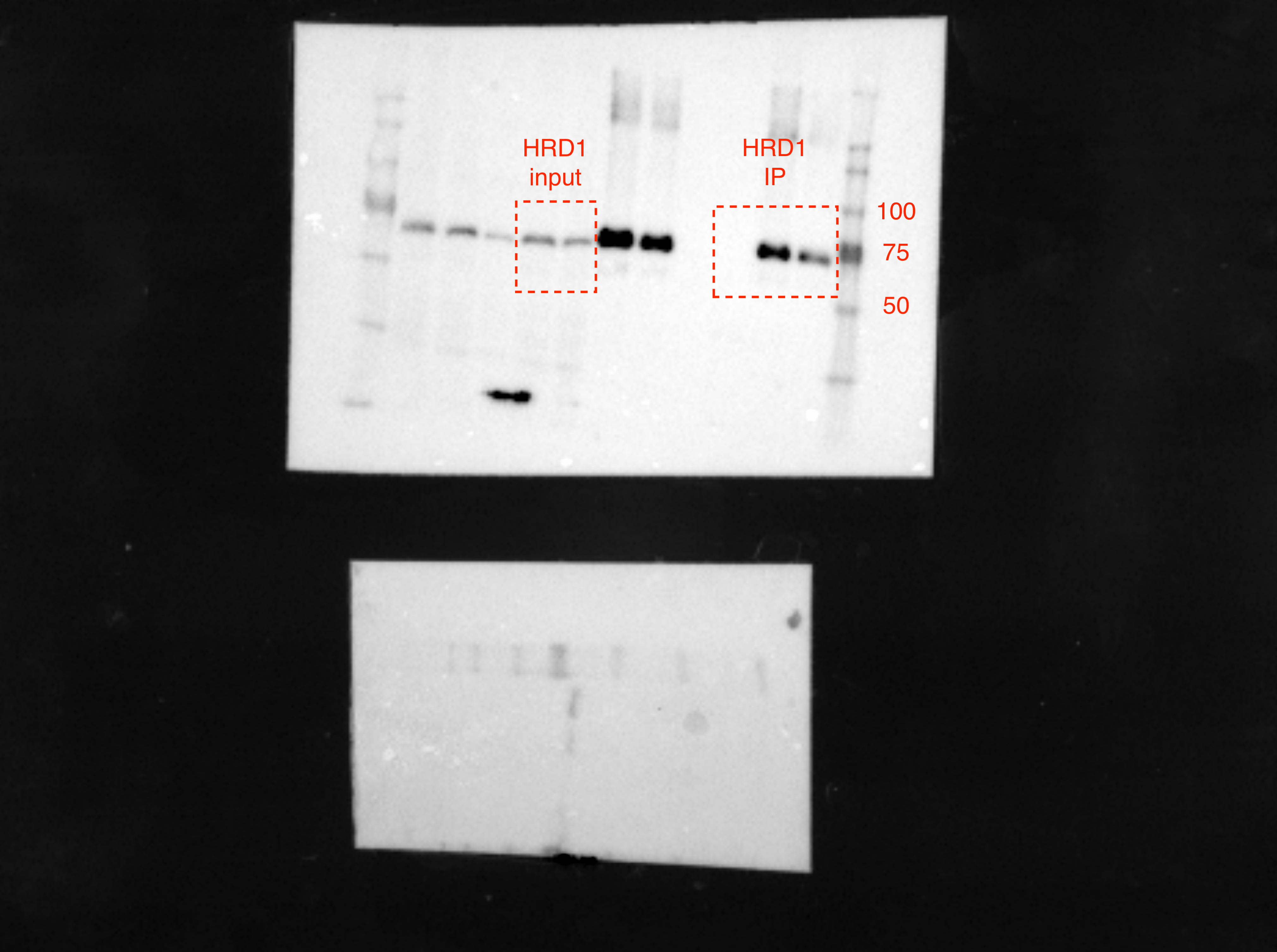

Supplement: Supplementary file 4 — Source data Fig. 2 [file 44318_2026_757_MOESM4_ESM.zip › Figure 2/Figure 2G/IP HRD1 merged with marker.tif]

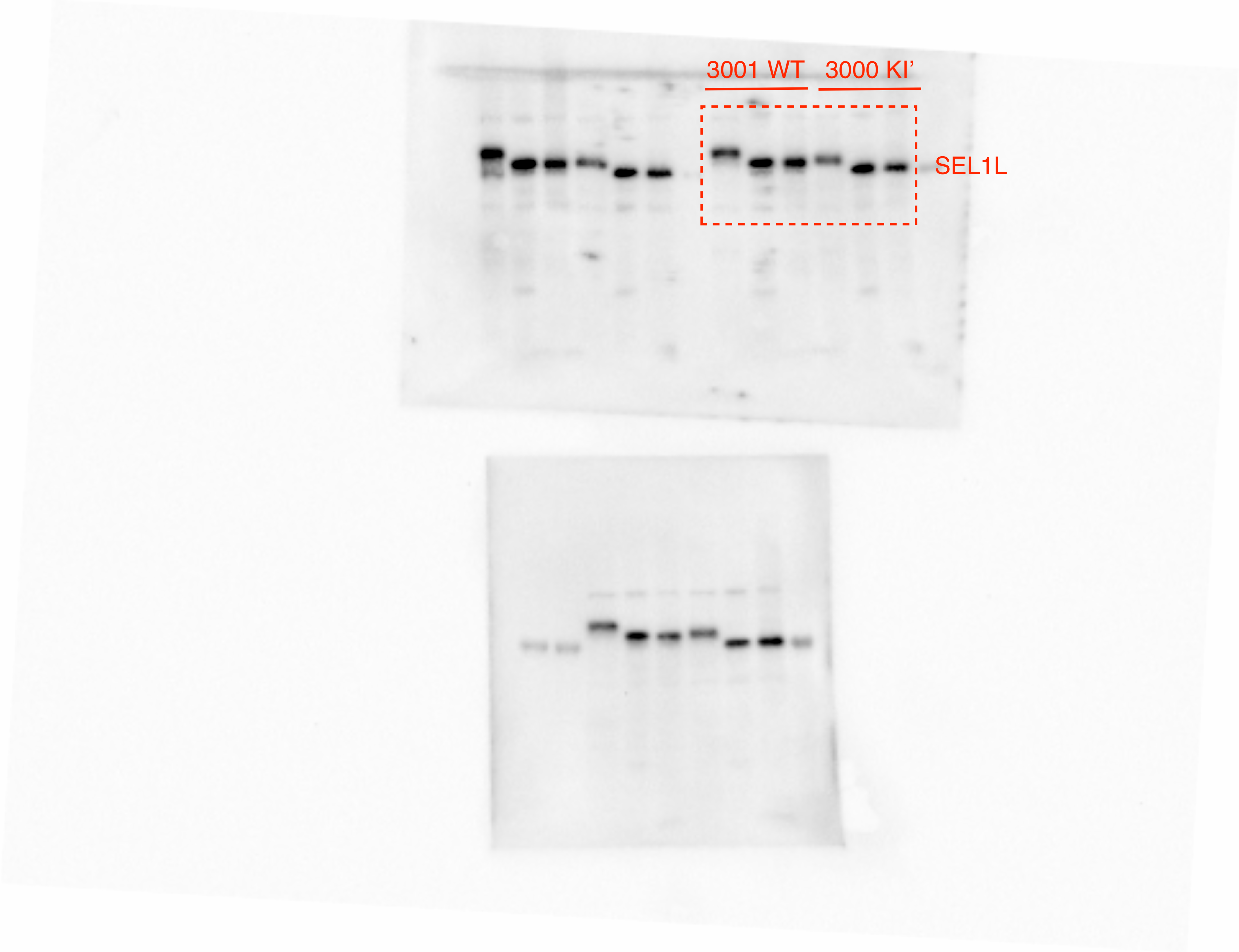

Supplement: Supplementary file 4 — Source data Fig. 2 [file 44318_2026_757_MOESM4_ESM.zip › Figure 2/Figure 2H/WB SEL1L no marker.tif]

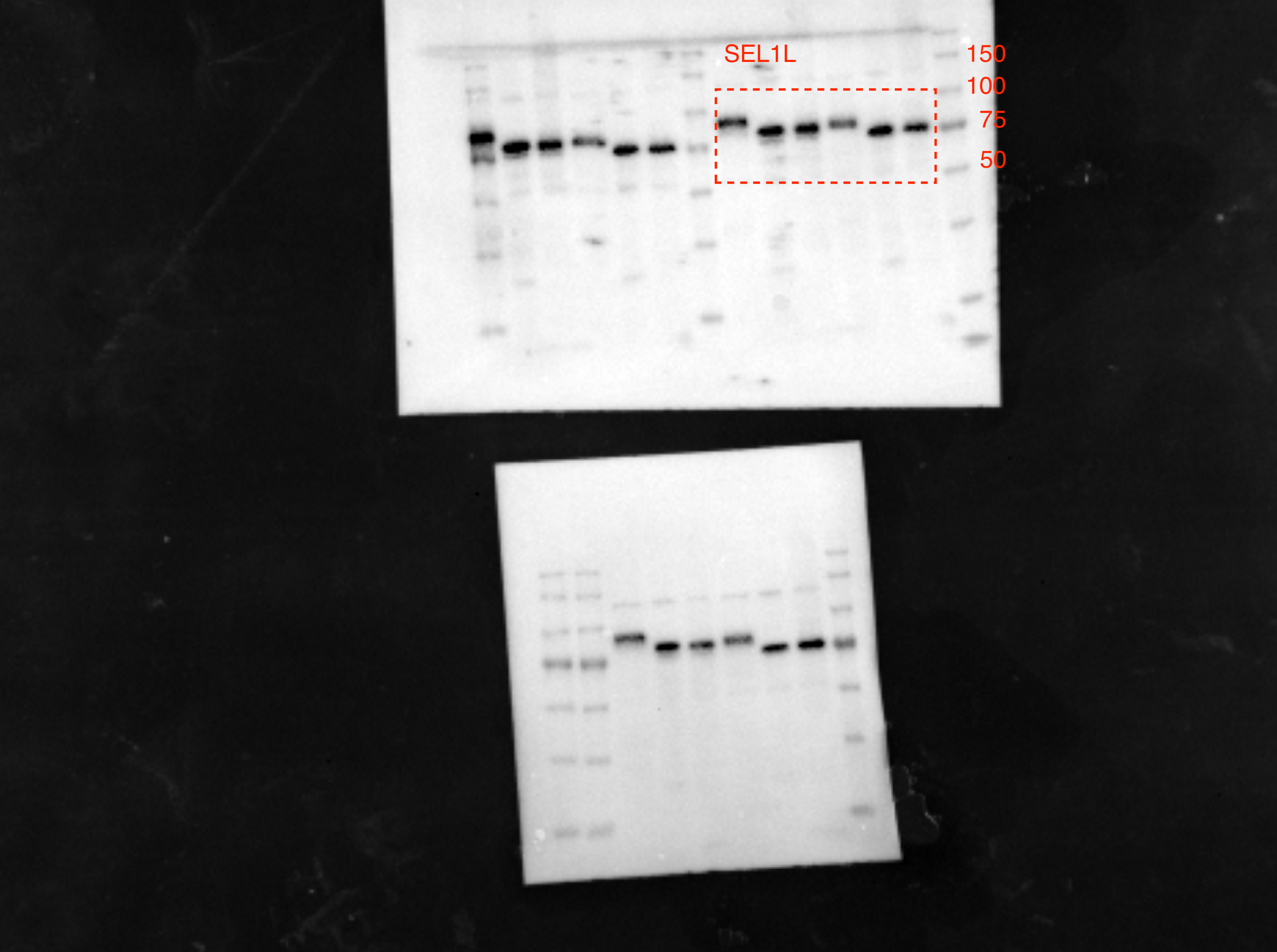

Supplement: Supplementary file 4 — Source data Fig. 2 [file 44318_2026_757_MOESM4_ESM.zip › Figure 2/Figure 2H/WB SEL1L merged with marker.tif]

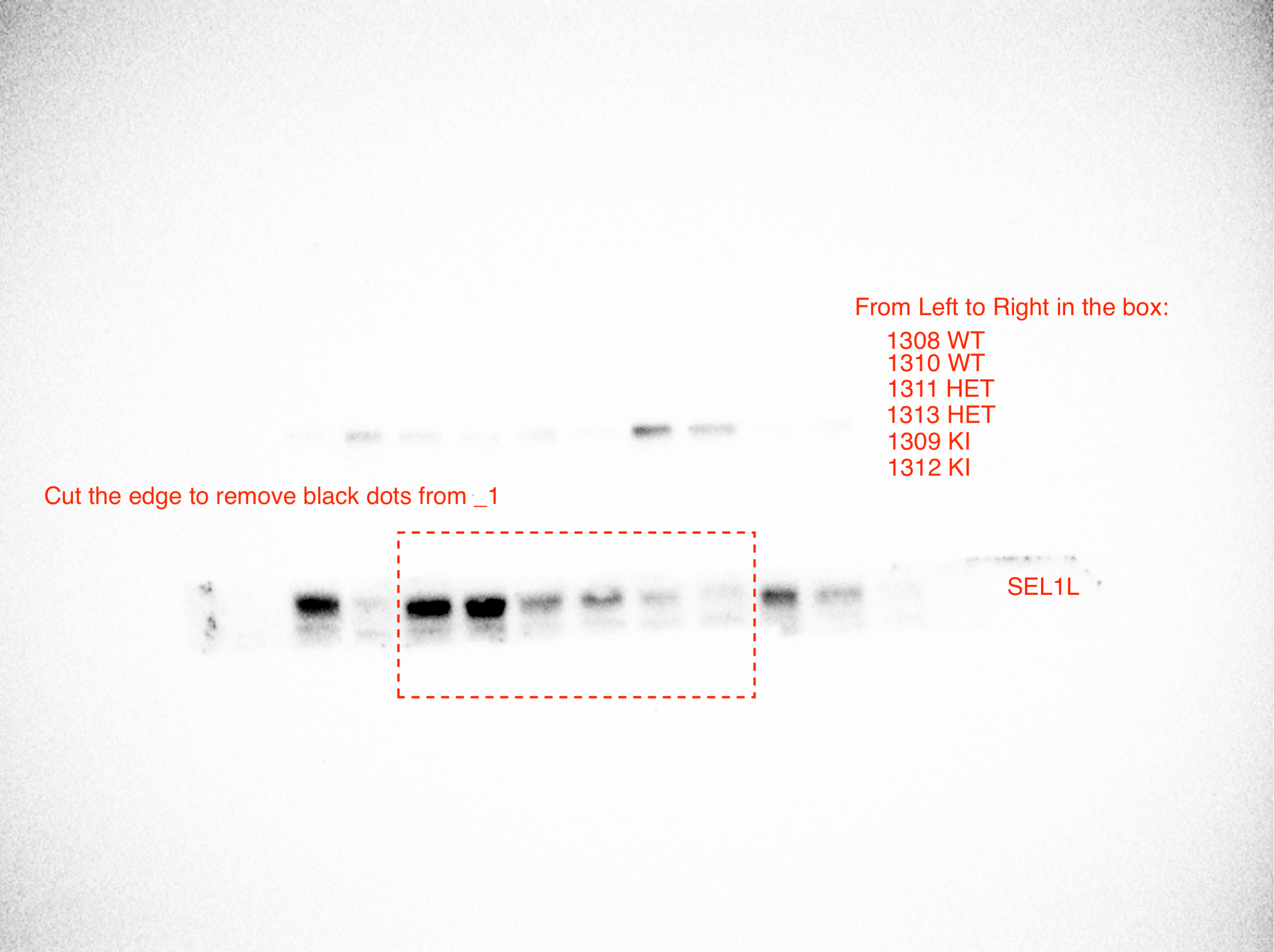

Supplement: Supplementary file 4 — Source data Fig. 2 [file 44318_2026_757_MOESM4_ESM.zip › Figure 2/Figure 2F/WT SEL1L Line B no marker_2.tif]

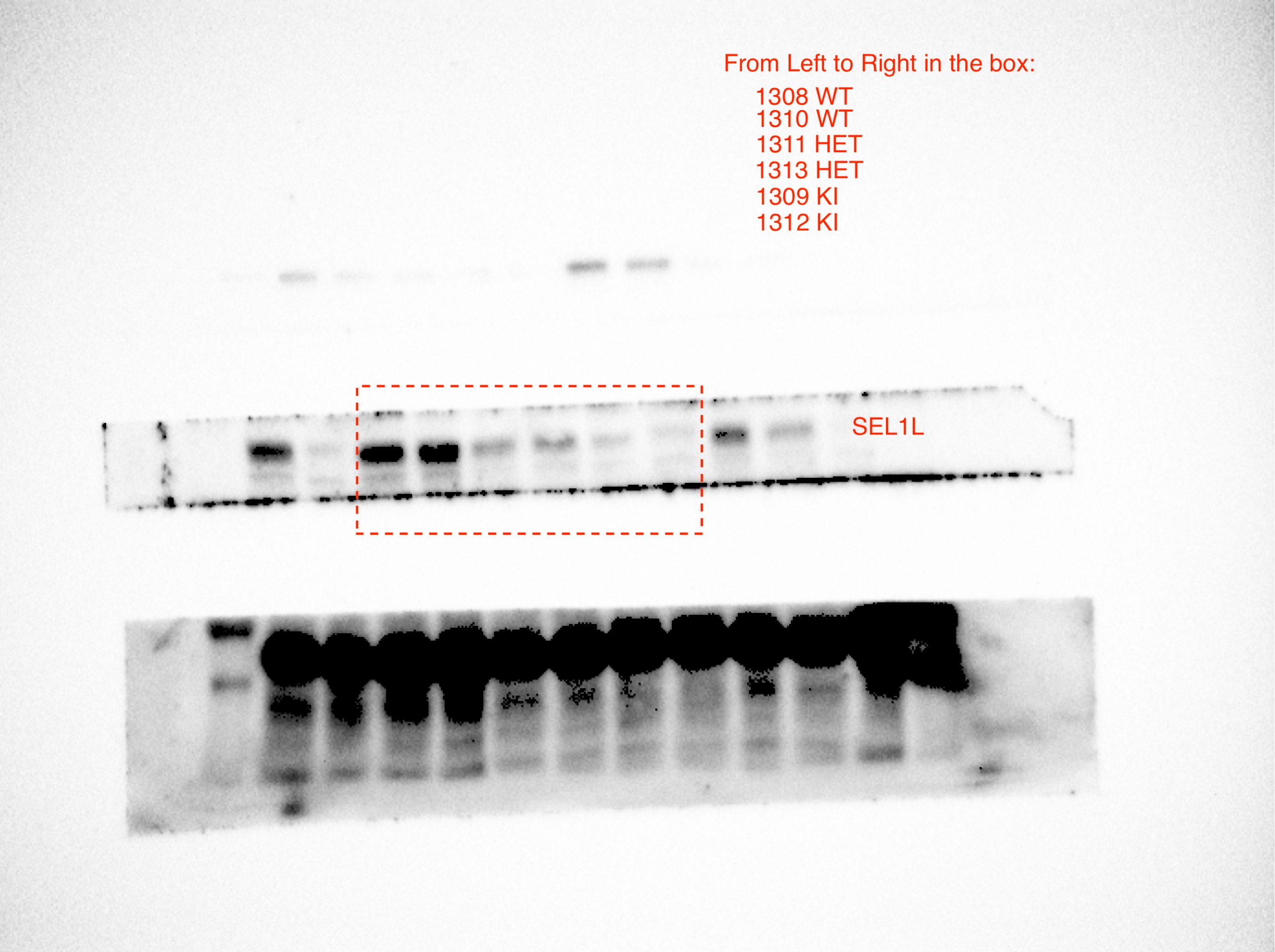

Supplement: Supplementary file 4 — Source data Fig. 2 [file 44318_2026_757_MOESM4_ESM.zip › Figure 2/Figure 2F/WT SEL1L Line B no marker_1.tif]

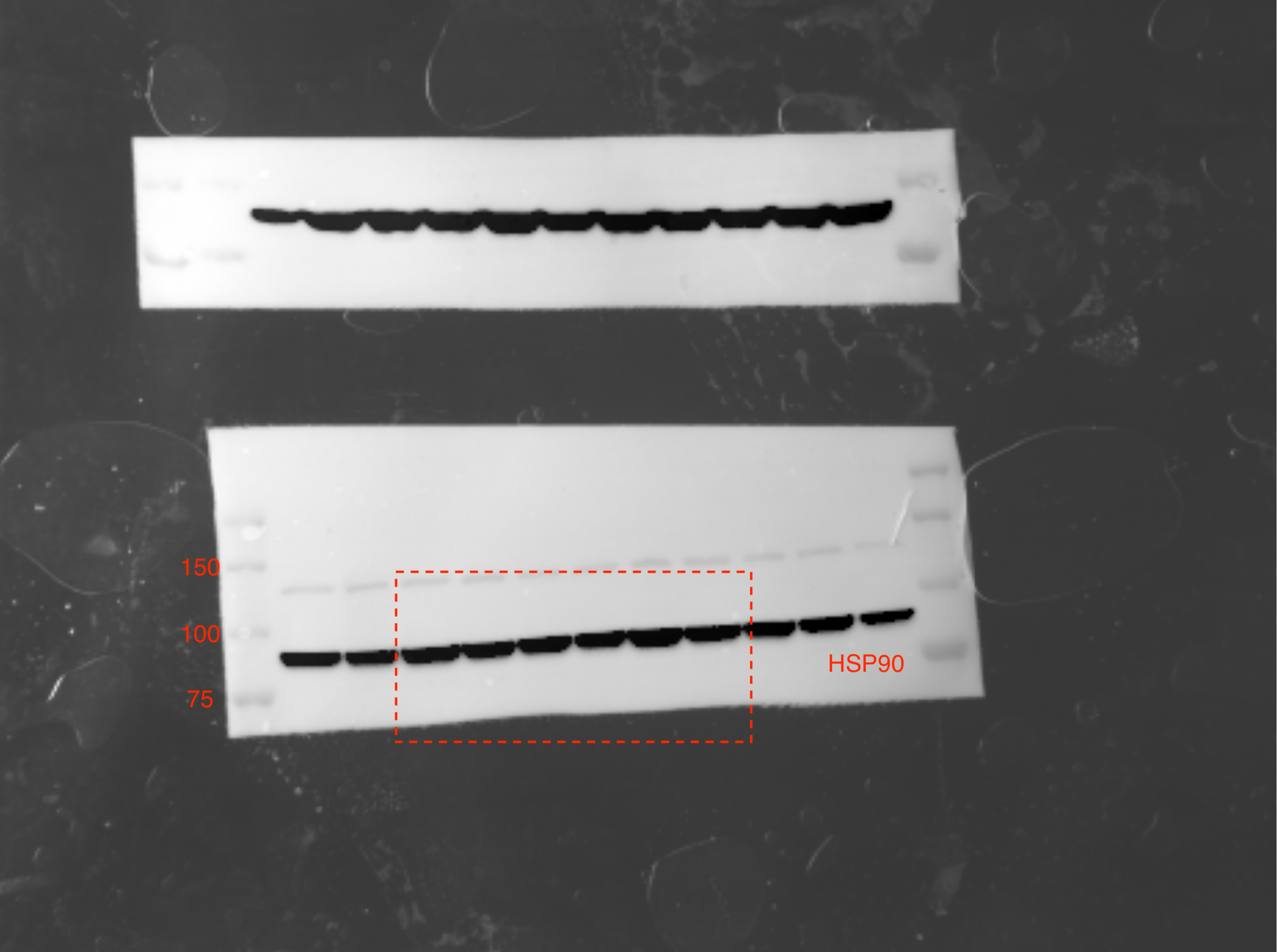

Supplement: Supplementary file 4 — Source data Fig. 2 [file 44318_2026_757_MOESM4_ESM.zip › Figure 2/Figure 2F/WB HSP90 for SEL1L Line B merged with marker.tif]

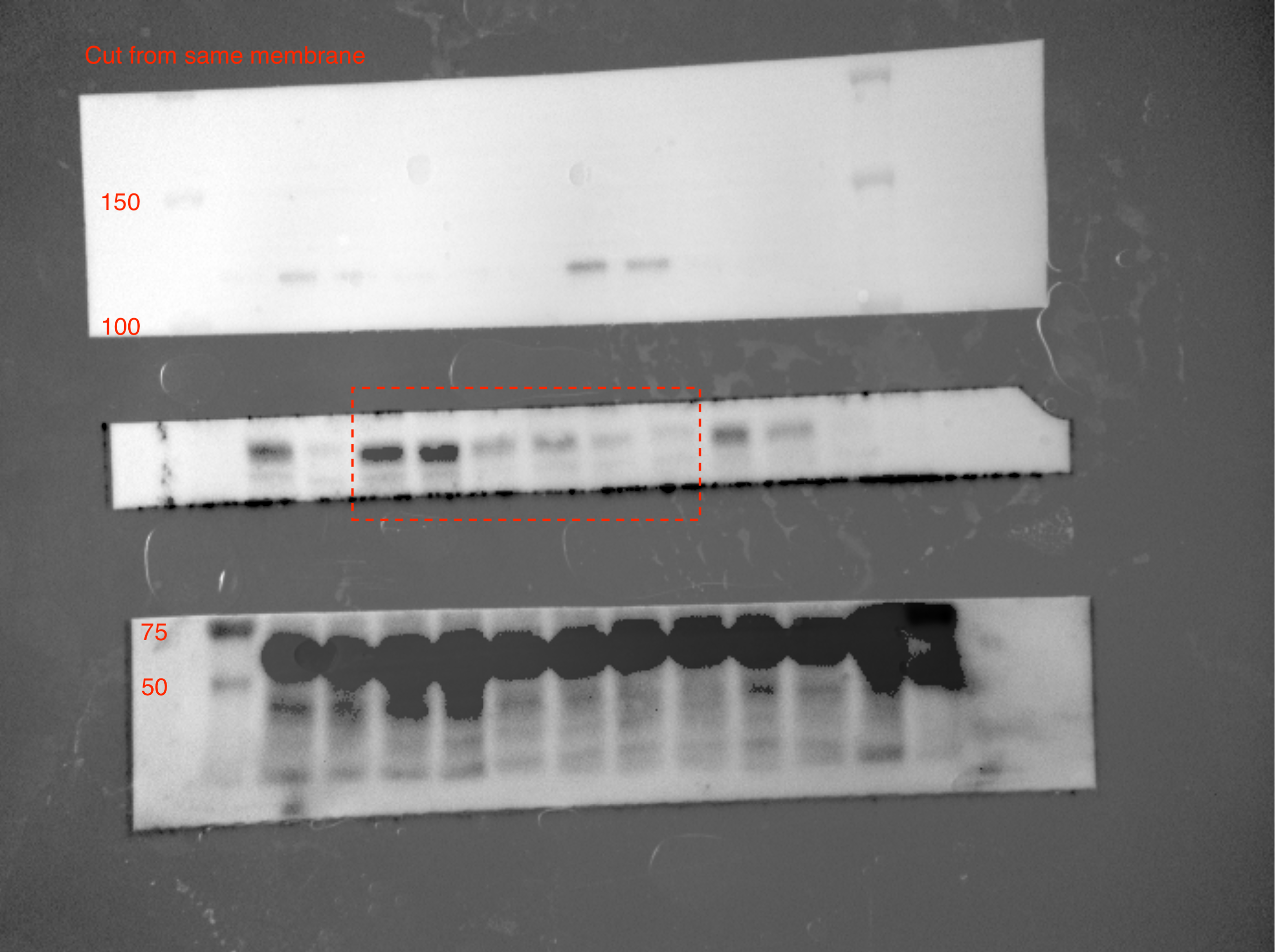

Supplement: Supplementary file 4 — Source data Fig. 2 [file 44318_2026_757_MOESM4_ESM.zip › Figure 2/Figure 2F/WT SEL1L Line B merged with marker.tif]

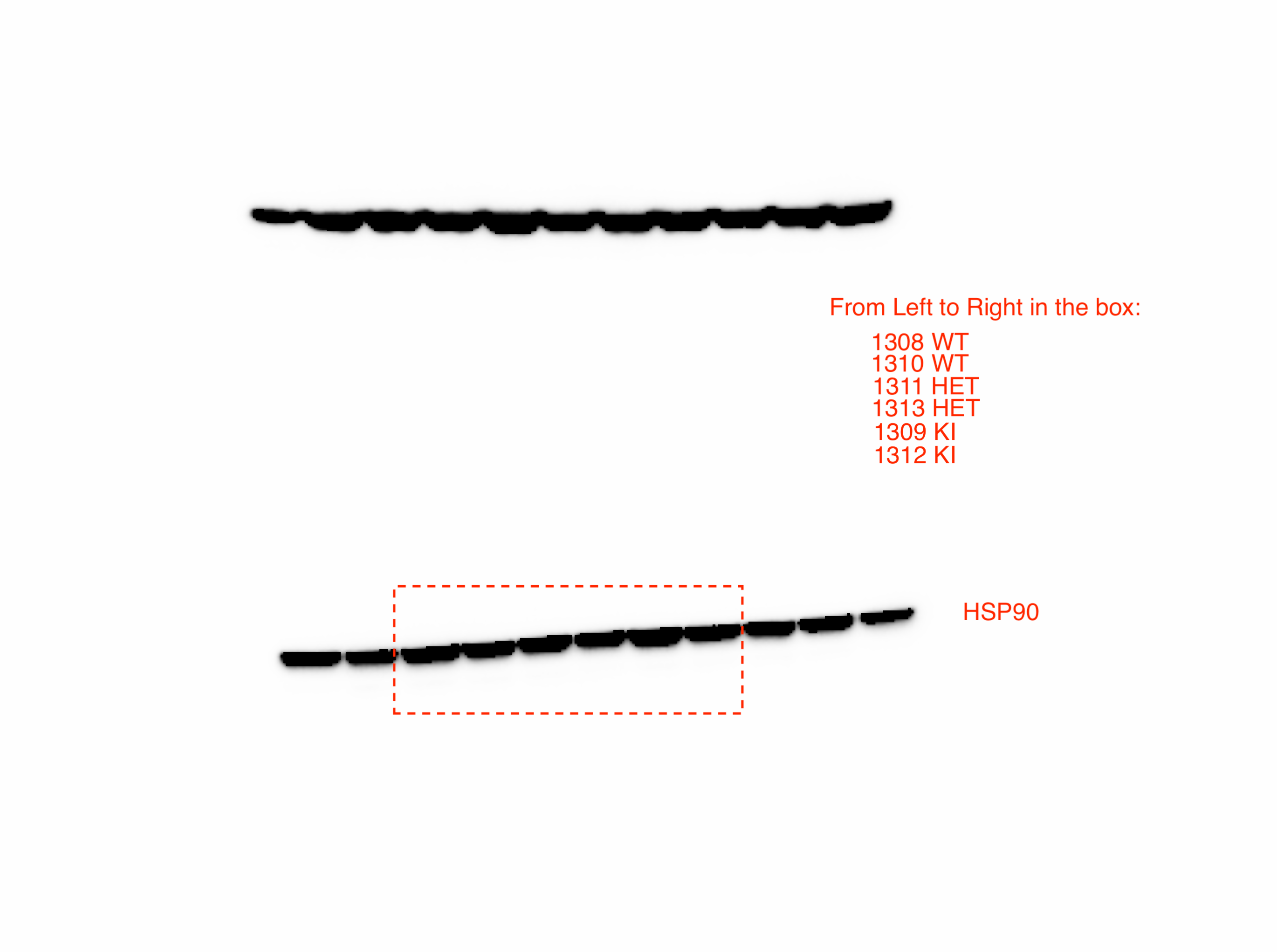

Supplement: Supplementary file 4 — Source data Fig. 2 [file 44318_2026_757_MOESM4_ESM.zip › Figure 2/Figure 2F/WB HSP90 for SEL1L Line B no marker.tif]

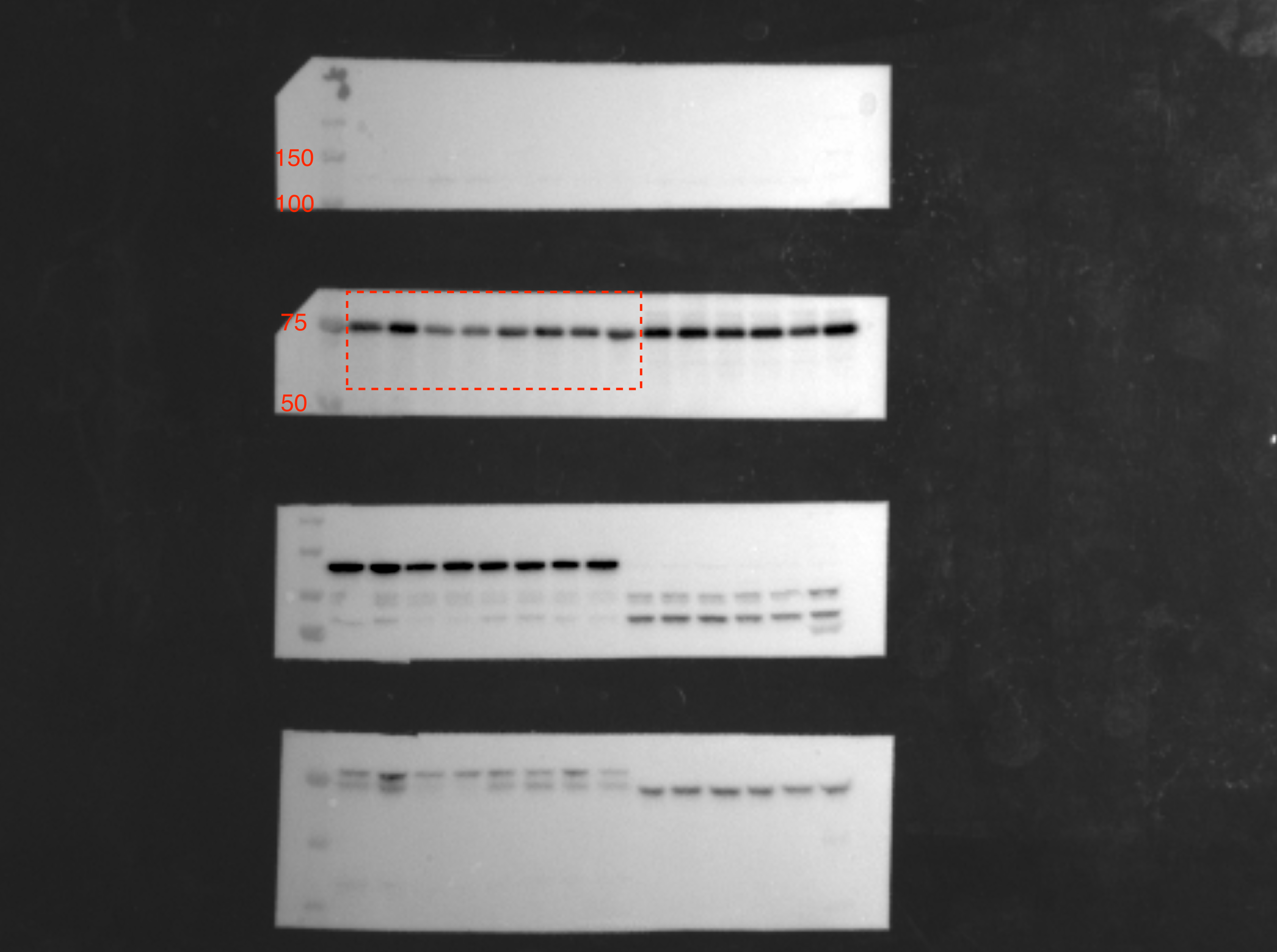

Supplement: Supplementary file 4 — Source data Fig. 2 [file 44318_2026_757_MOESM4_ESM.zip › Figure 2/Figure 2C/WB HRD1 merged with marker.tif]

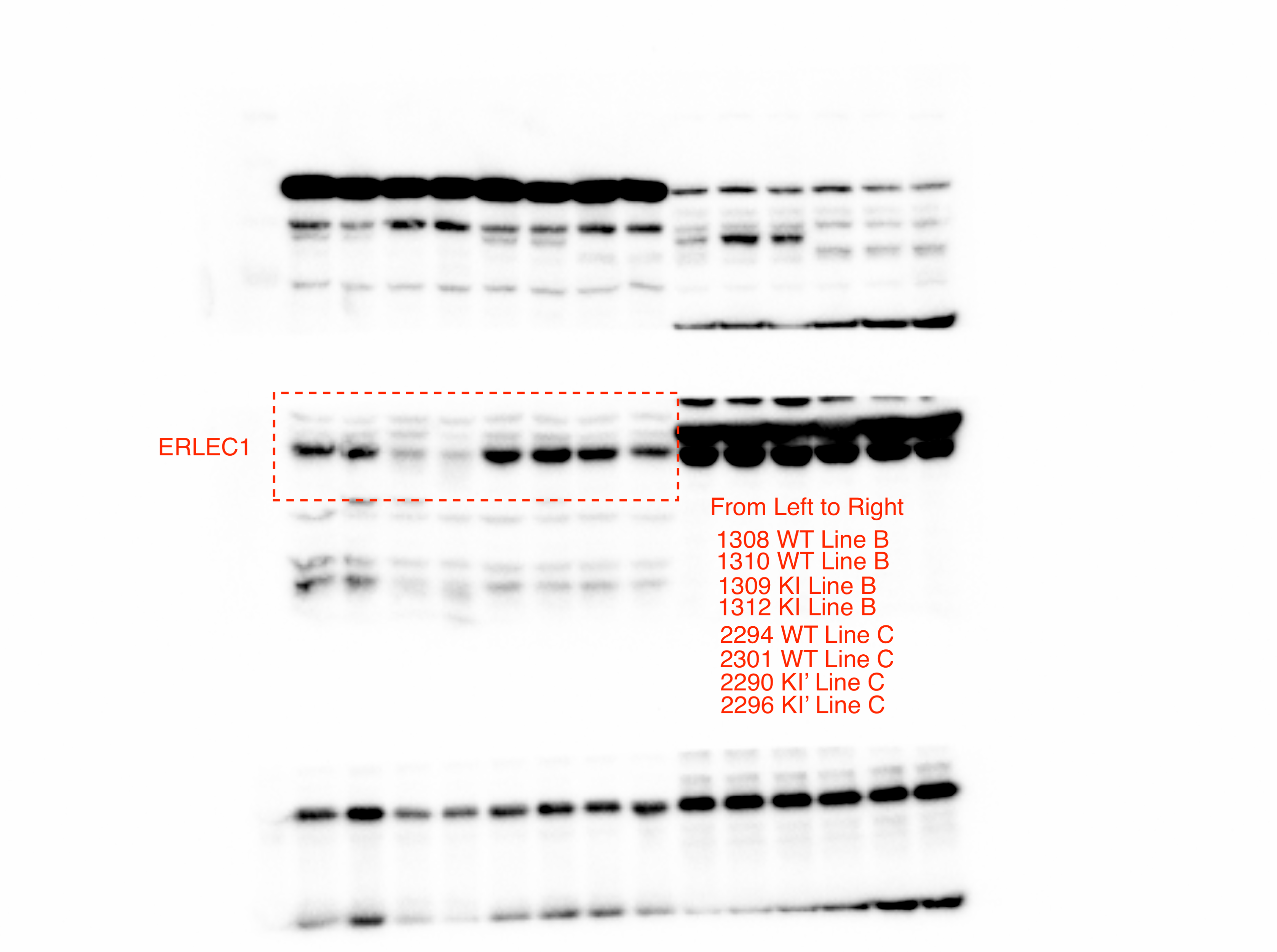

Supplement: Supplementary file 4 — Source data Fig. 2 [file 44318_2026_757_MOESM4_ESM.zip › Figure 2/Figure 2C/WB ERLEC1 no marker.tif]

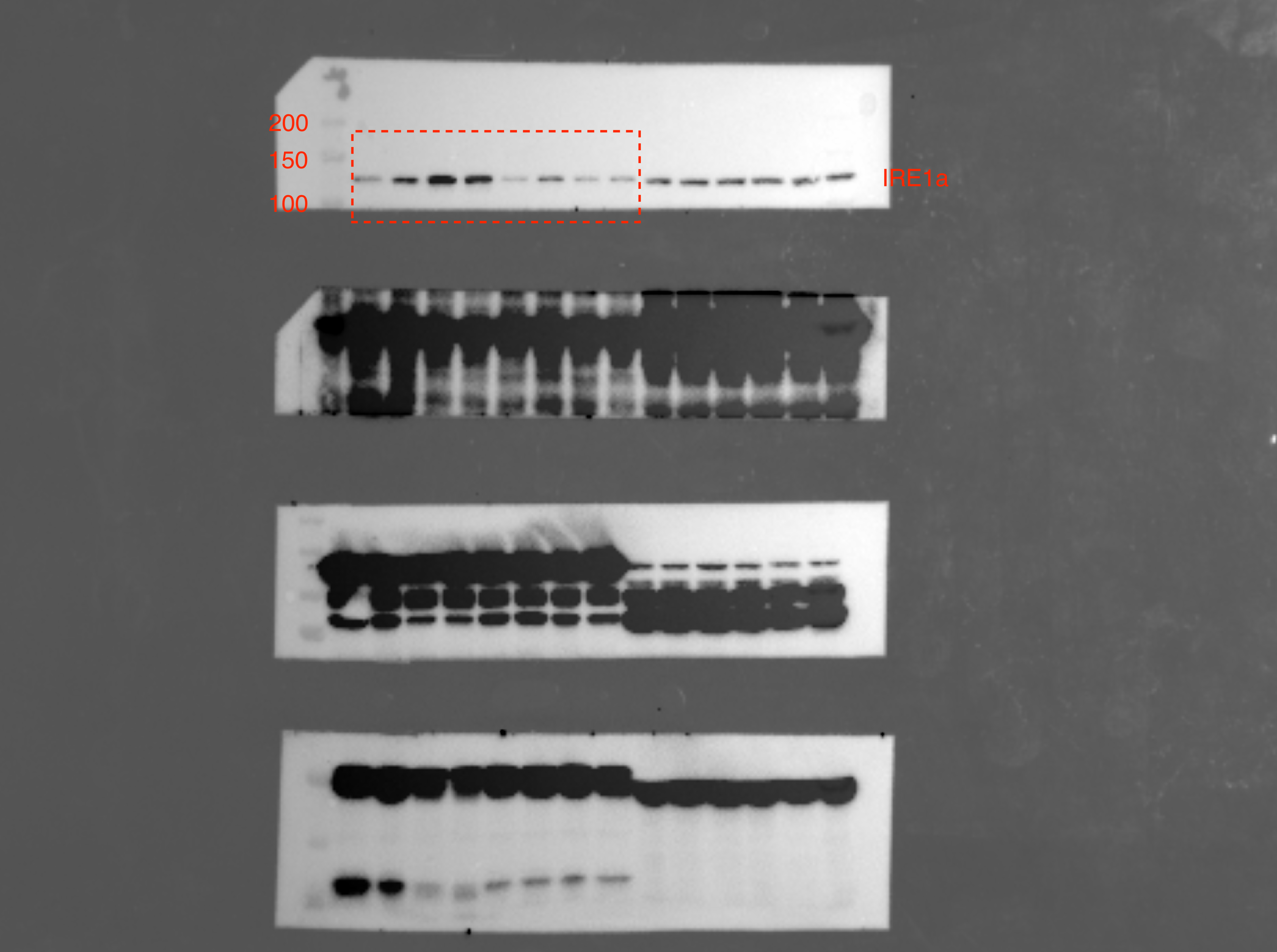

Supplement: Supplementary file 4 — Source data Fig. 2 [file 44318_2026_757_MOESM4_ESM.zip › Figure 2/Figure 2C/WB IRE1a merged with marker.tif]

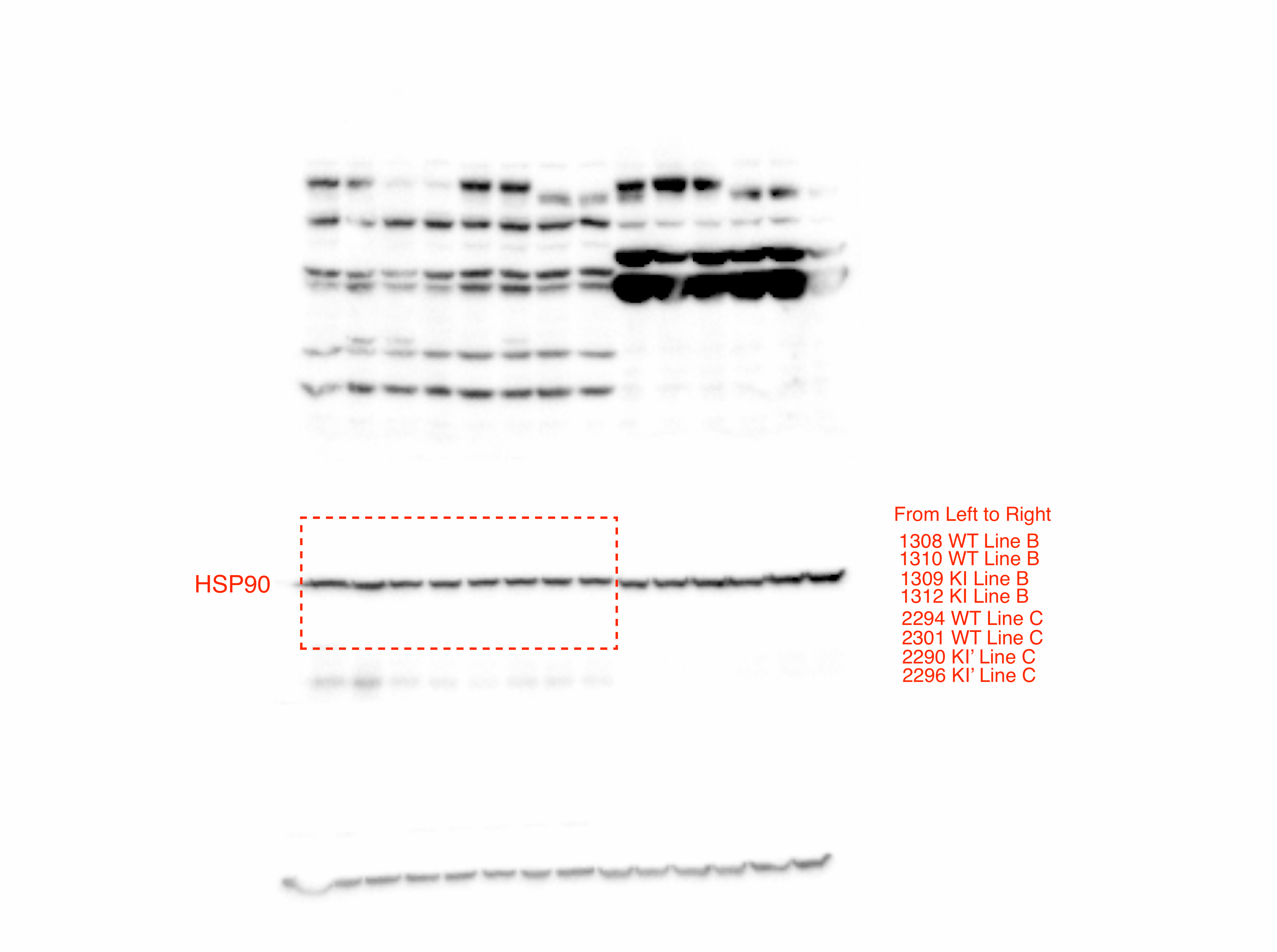

Supplement: Supplementary file 4 — Source data Fig. 2 [file 44318_2026_757_MOESM4_ESM.zip › Figure 2/Figure 2C/WB HSP90 no marker.tif]

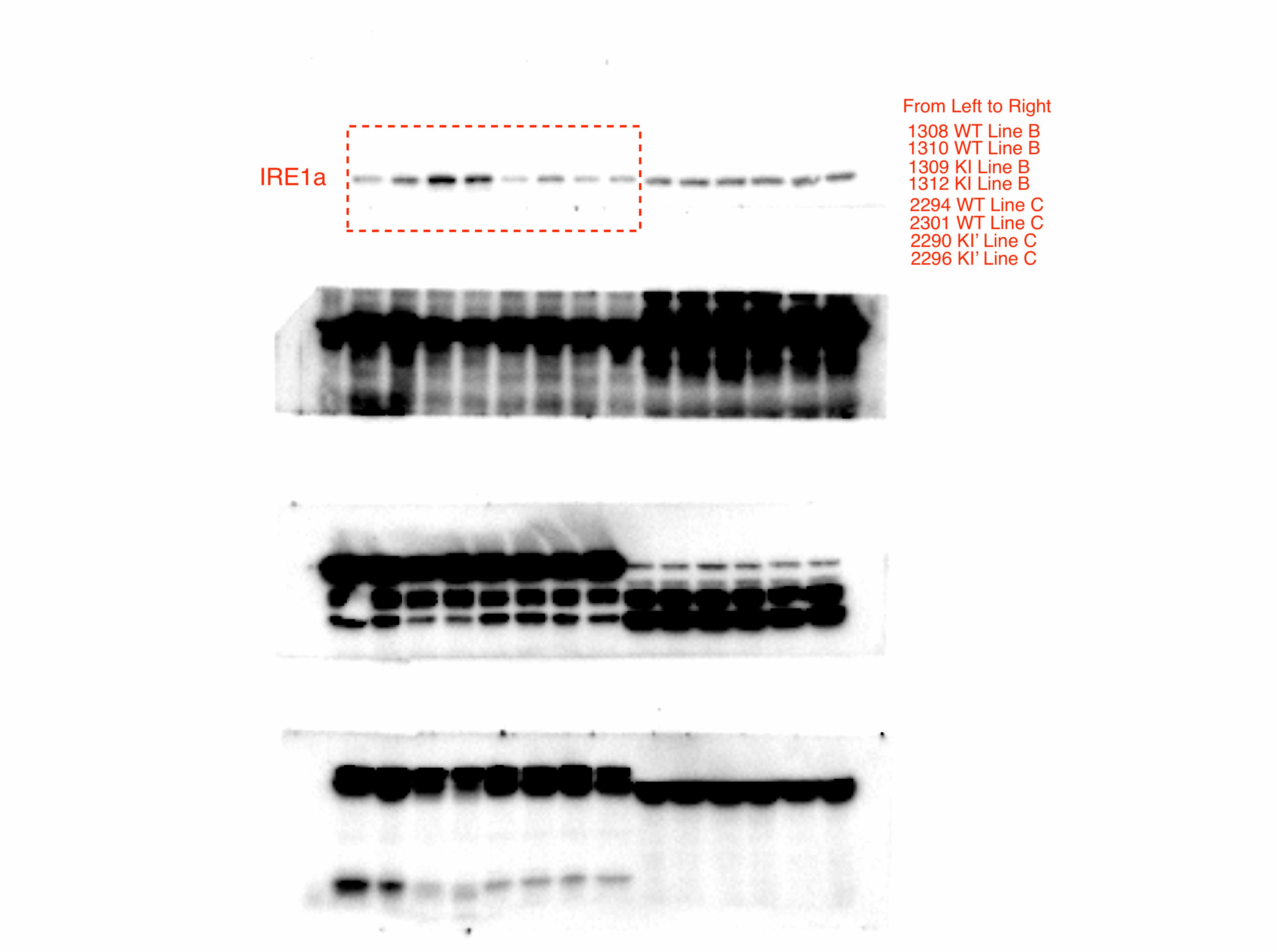

Supplement: Supplementary file 4 — Source data Fig. 2 [file 44318_2026_757_MOESM4_ESM.zip › Figure 2/Figure 2C/WB IRE1a no marker.tif]

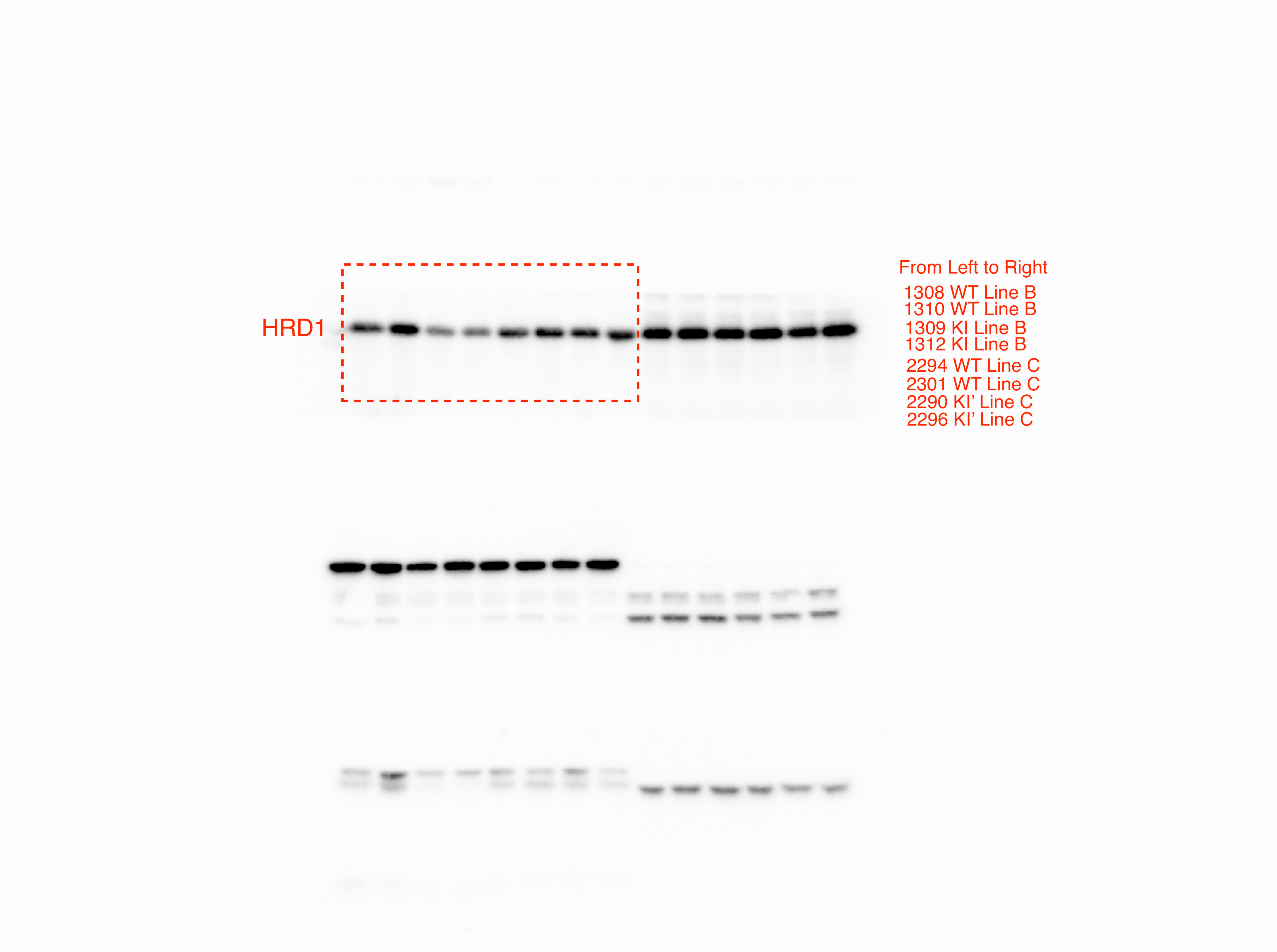

Supplement: Supplementary file 4 — Source data Fig. 2 [file 44318_2026_757_MOESM4_ESM.zip › Figure 2/Figure 2C/WB HRD1 no marker.tif]

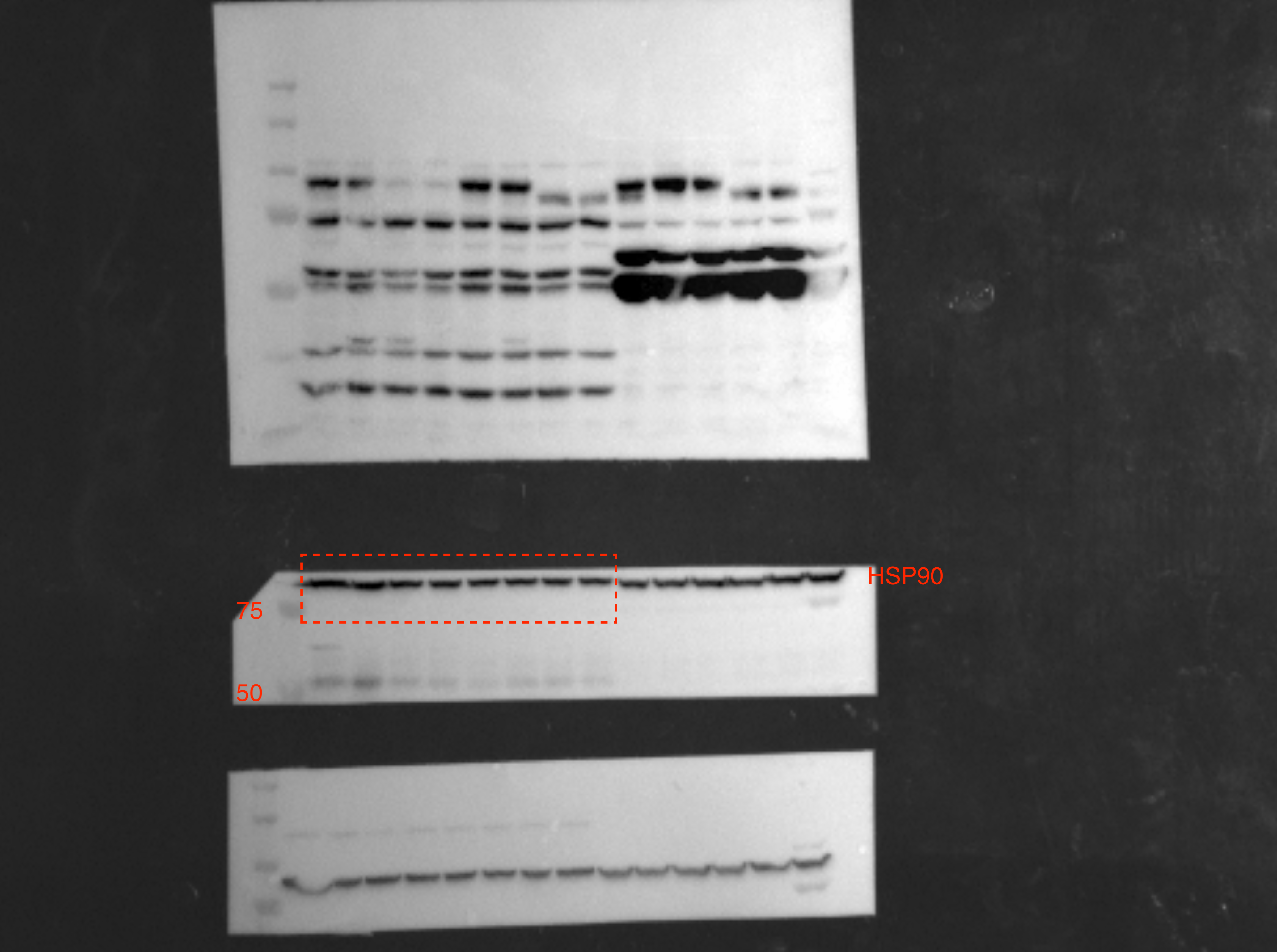

Supplement: Supplementary file 4 — Source data Fig. 2 [file 44318_2026_757_MOESM4_ESM.zip › Figure 2/Figure 2C/WB HSP90 merged with marker.tif]

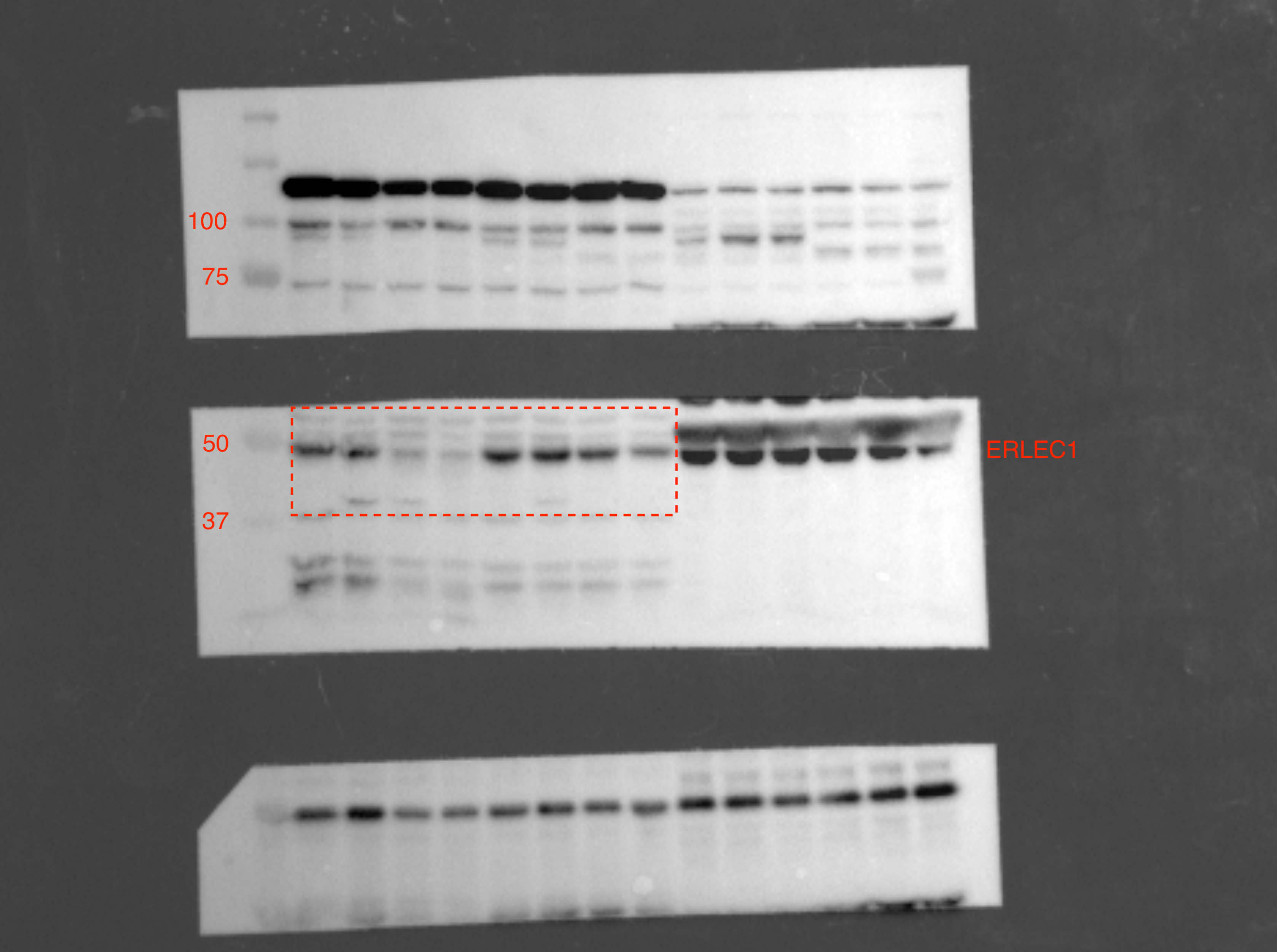

Supplement: Supplementary file 4 — Source data Fig. 2 [file 44318_2026_757_MOESM4_ESM.zip › Figure 2/Figure 2C/WB ERLEC1 merged with marker.tif]

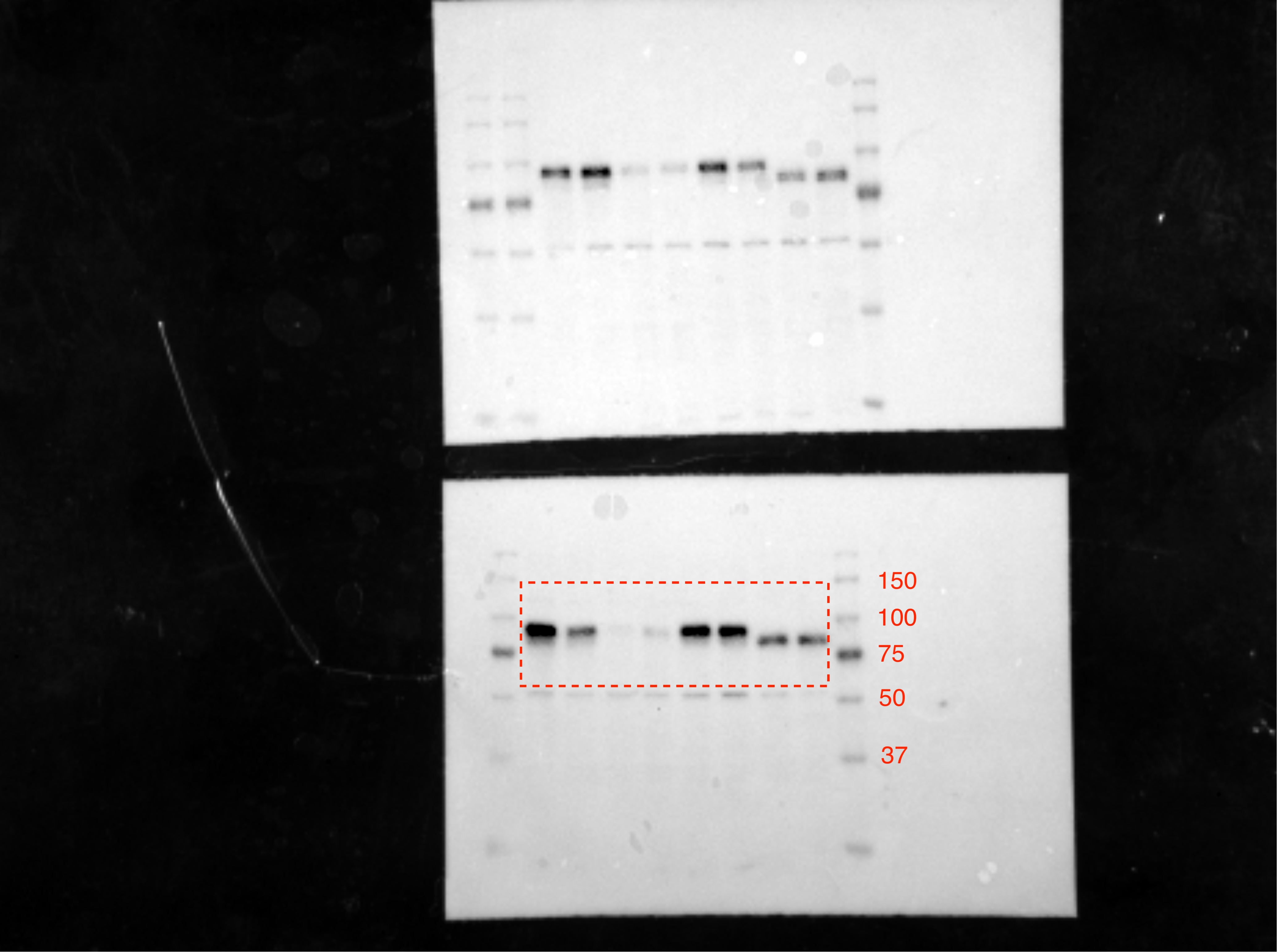

Supplement: Supplementary file 4 — Source data Fig. 2 [file 44318_2026_757_MOESM4_ESM.zip › Figure 2/Figure 2B/WB SEL1L Ab1 merged with marker.tif]

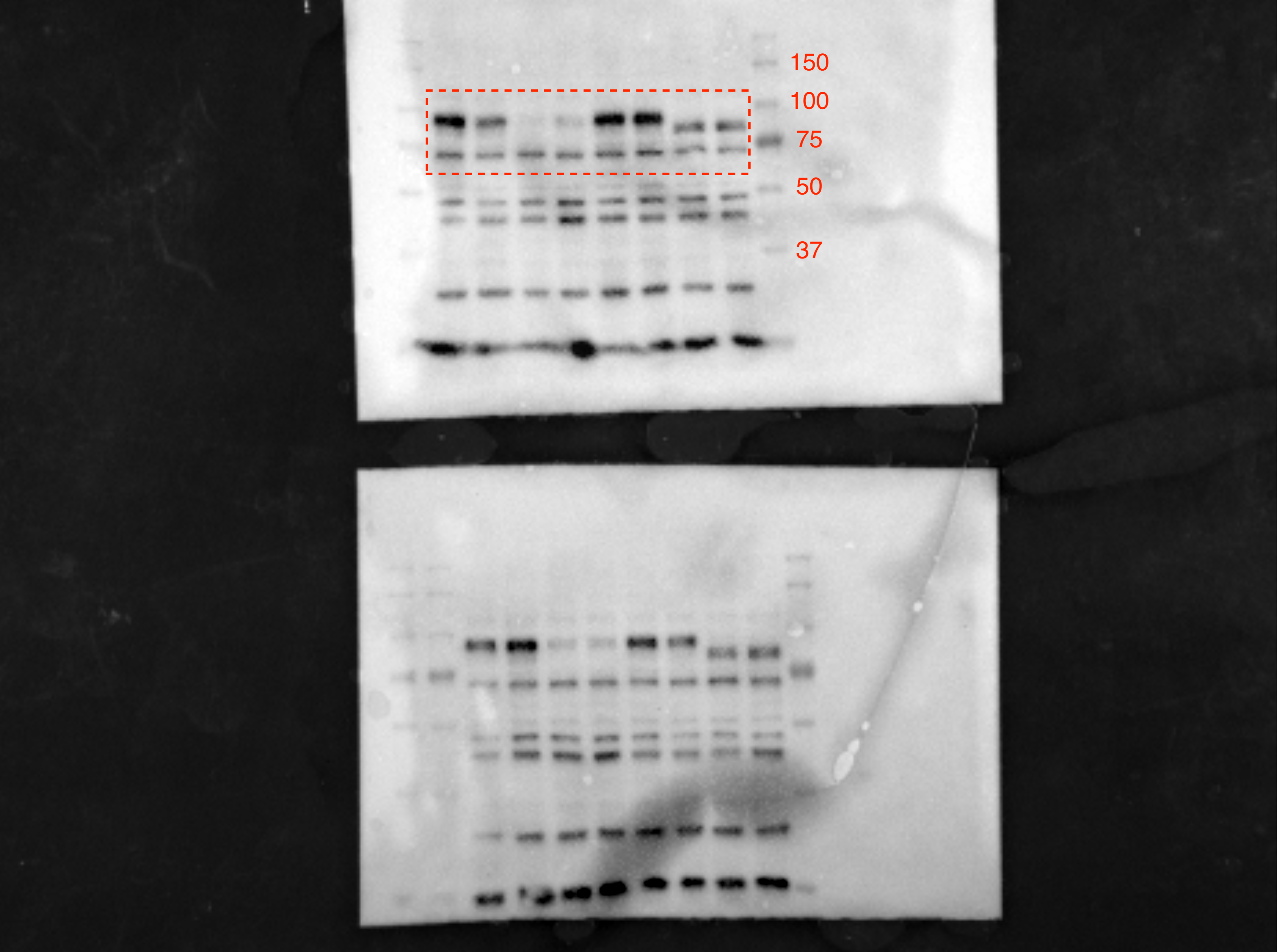

Supplement: Supplementary file 4 — Source data Fig. 2 [file 44318_2026_757_MOESM4_ESM.zip › Figure 2/Figure 2B/WB SEL1L Ab2 merged with marker.tif]

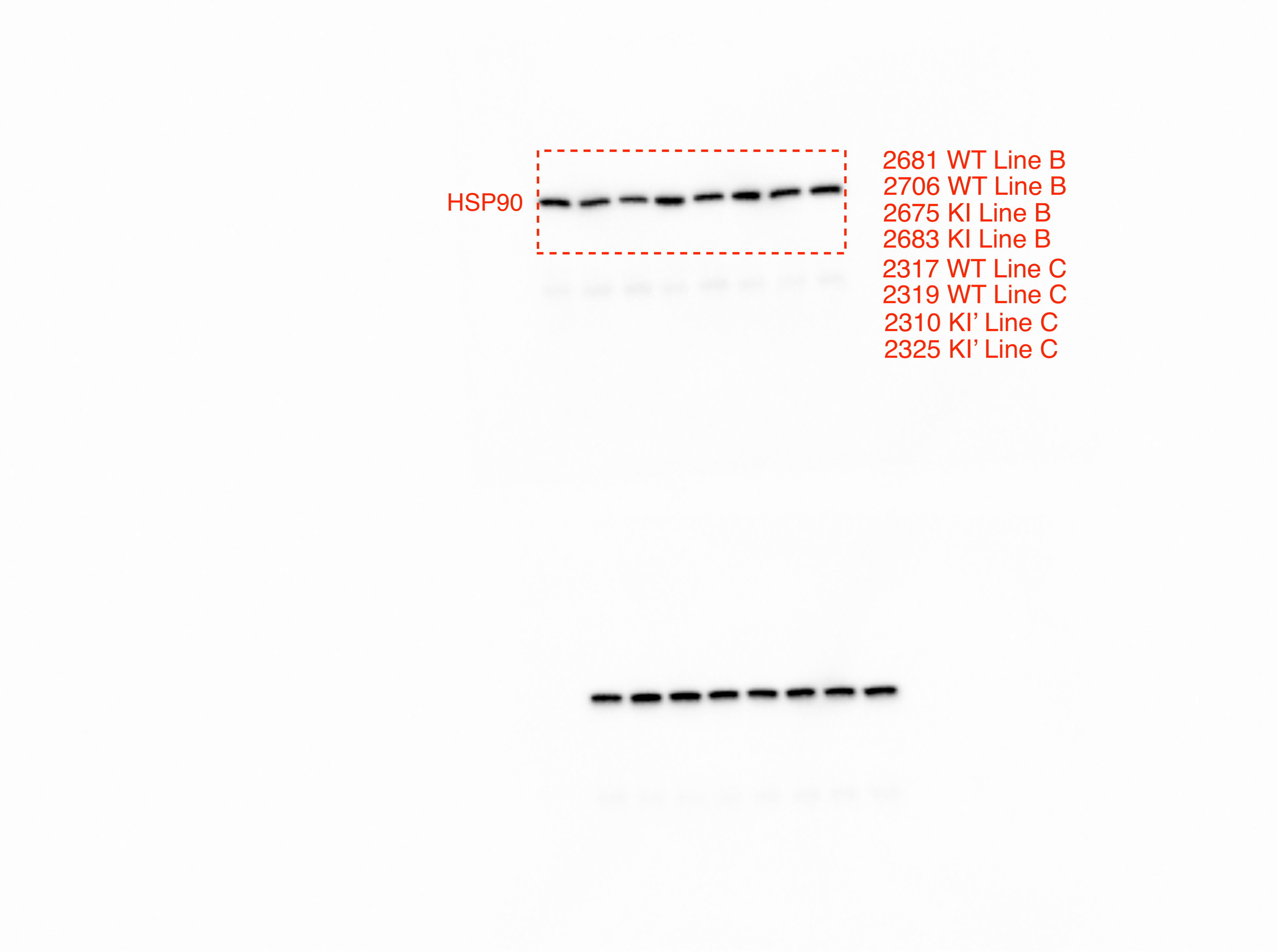

Supplement: Supplementary file 4 — Source data Fig. 2 [file 44318_2026_757_MOESM4_ESM.zip › Figure 2/Figure 2B/WB HSP90 no marker.tif]

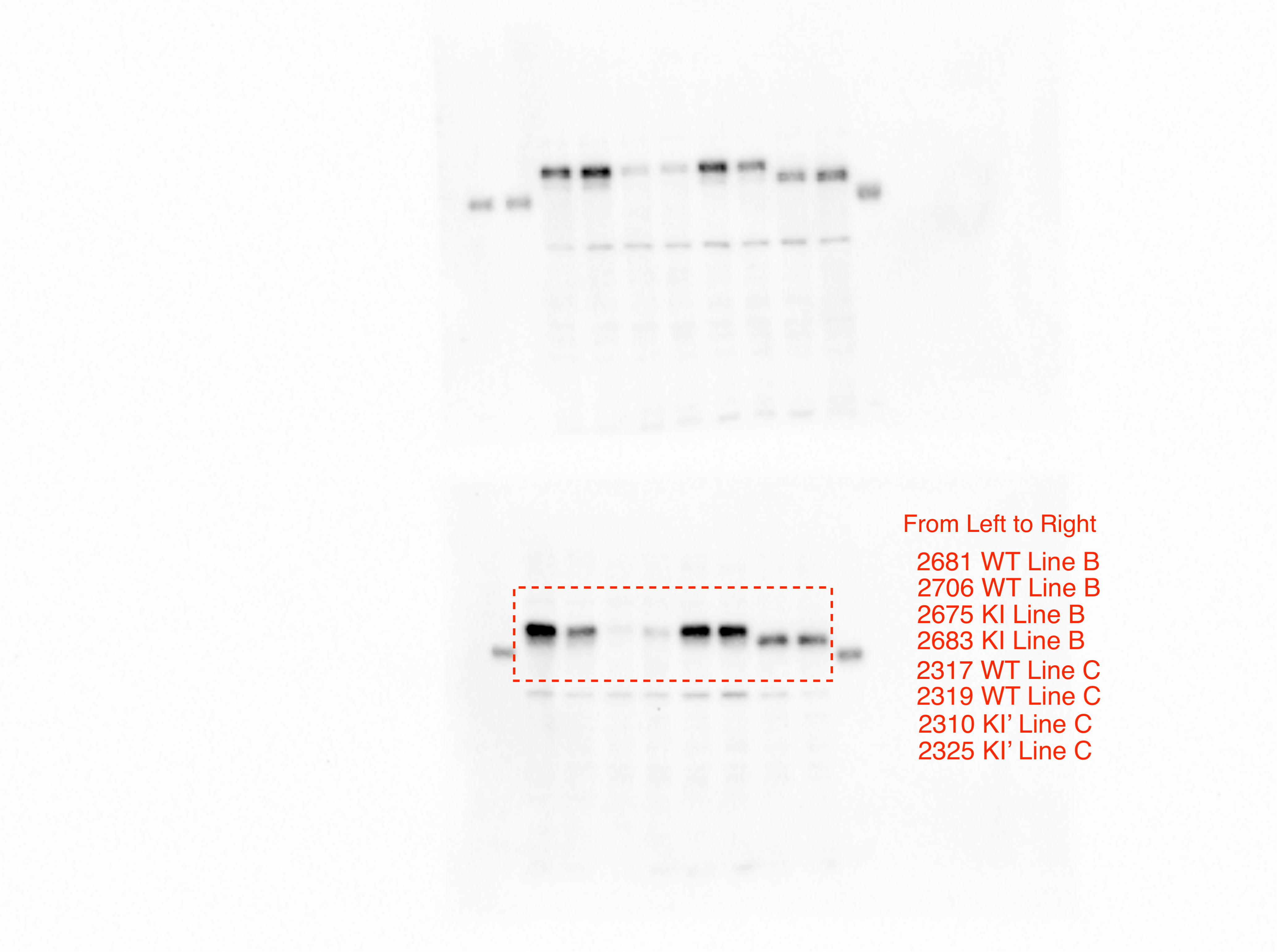

Supplement: Supplementary file 4 — Source data Fig. 2 [file 44318_2026_757_MOESM4_ESM.zip › Figure 2/Figure 2B/WB SEL1L Ab1 no marker.tif]

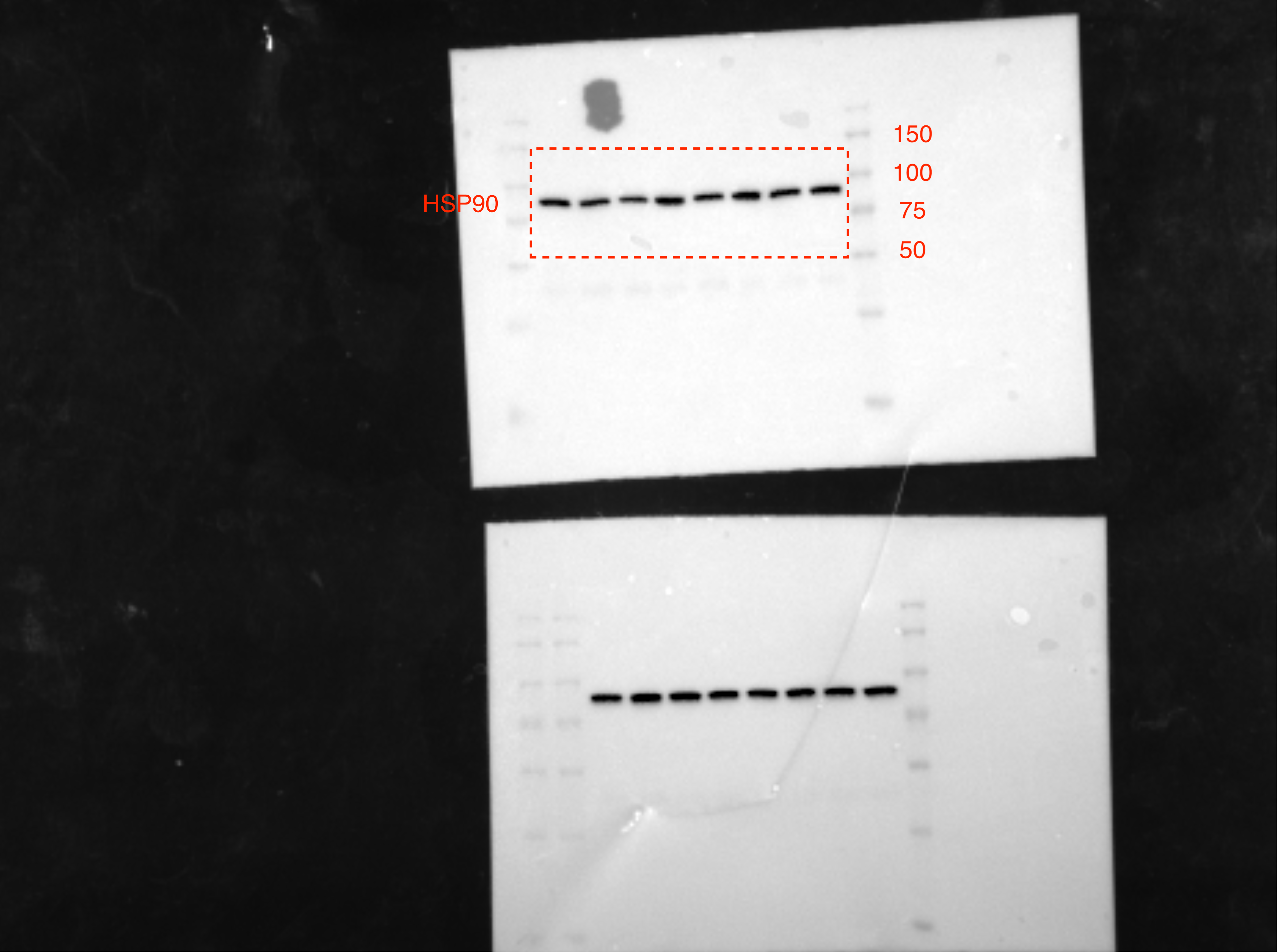

Supplement: Supplementary file 4 — Source data Fig. 2 [file 44318_2026_757_MOESM4_ESM.zip › Figure 2/Figure 2B/WB HSP90 merged with marker.tif]

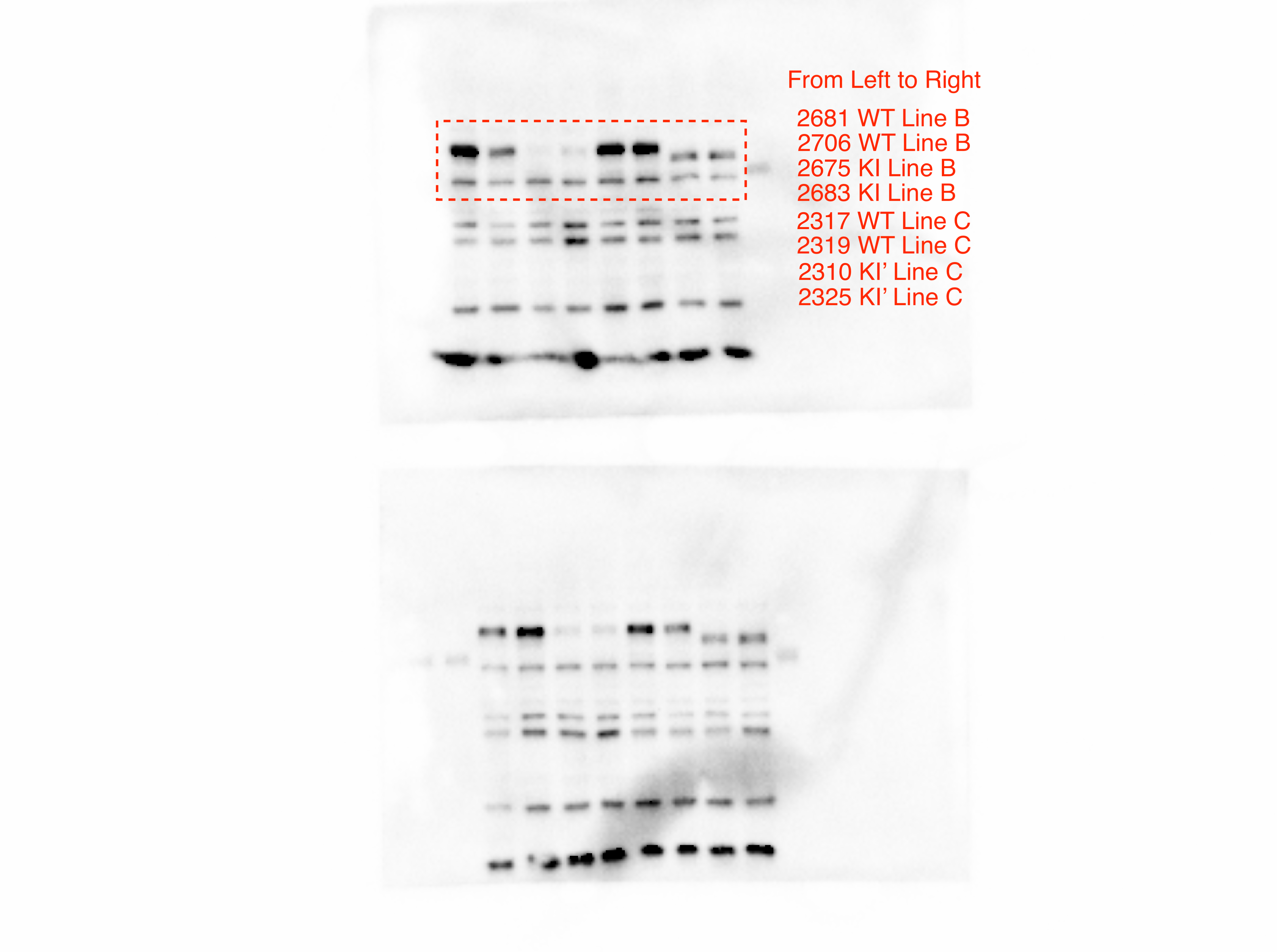

Supplement: Supplementary file 4 — Source data Fig. 2 [file 44318_2026_757_MOESM4_ESM.zip › Figure 2/Figure 2B/WB SEL1L Ab2 no marker.tif]

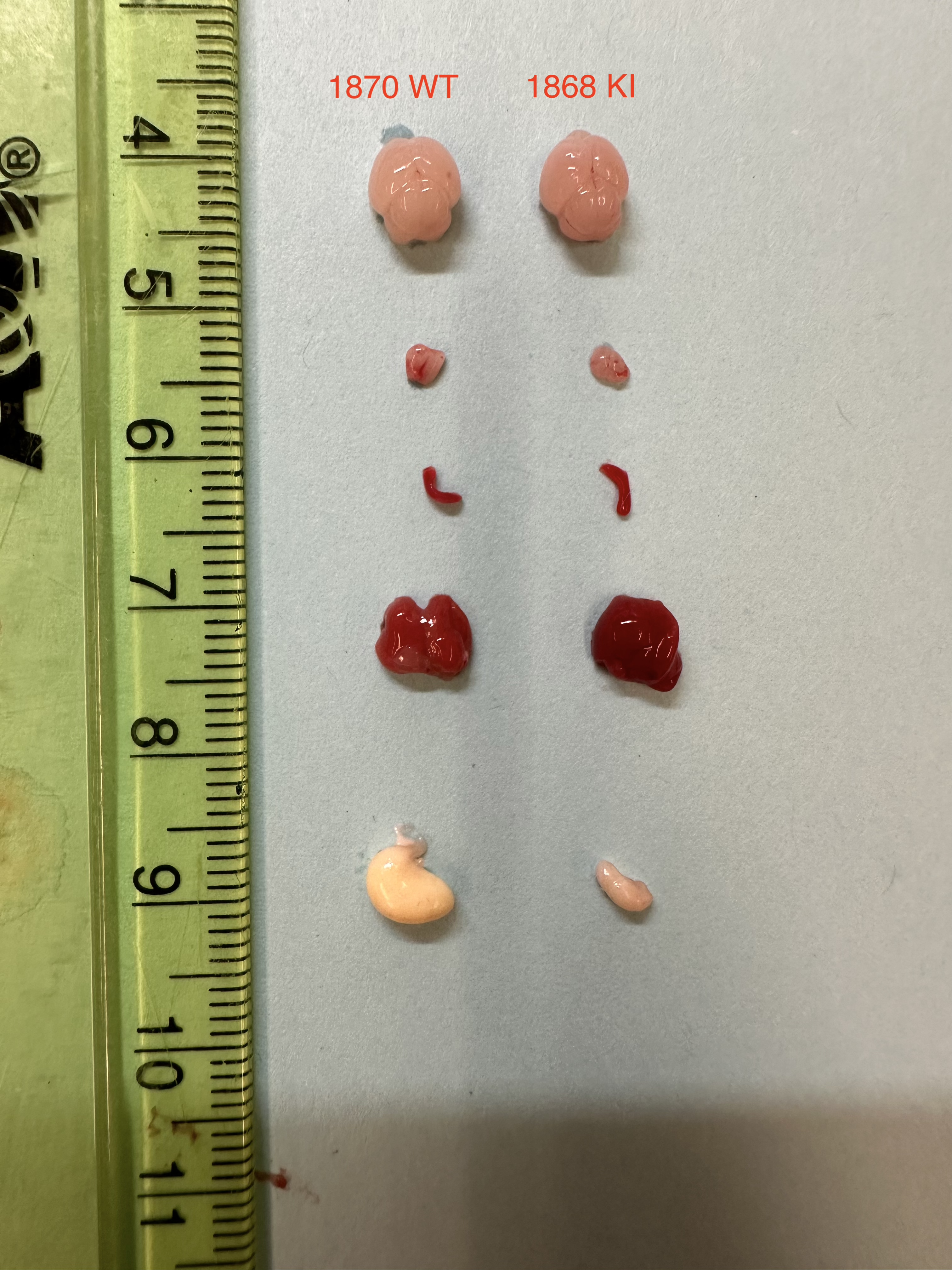

Supplement: Supplementary file 6 — Source data Fig. 4 [file 44318_2026_757_MOESM6_ESM.zip › Figure 4/Figure 4E/Line A Mouse Tissue photo.tiff]

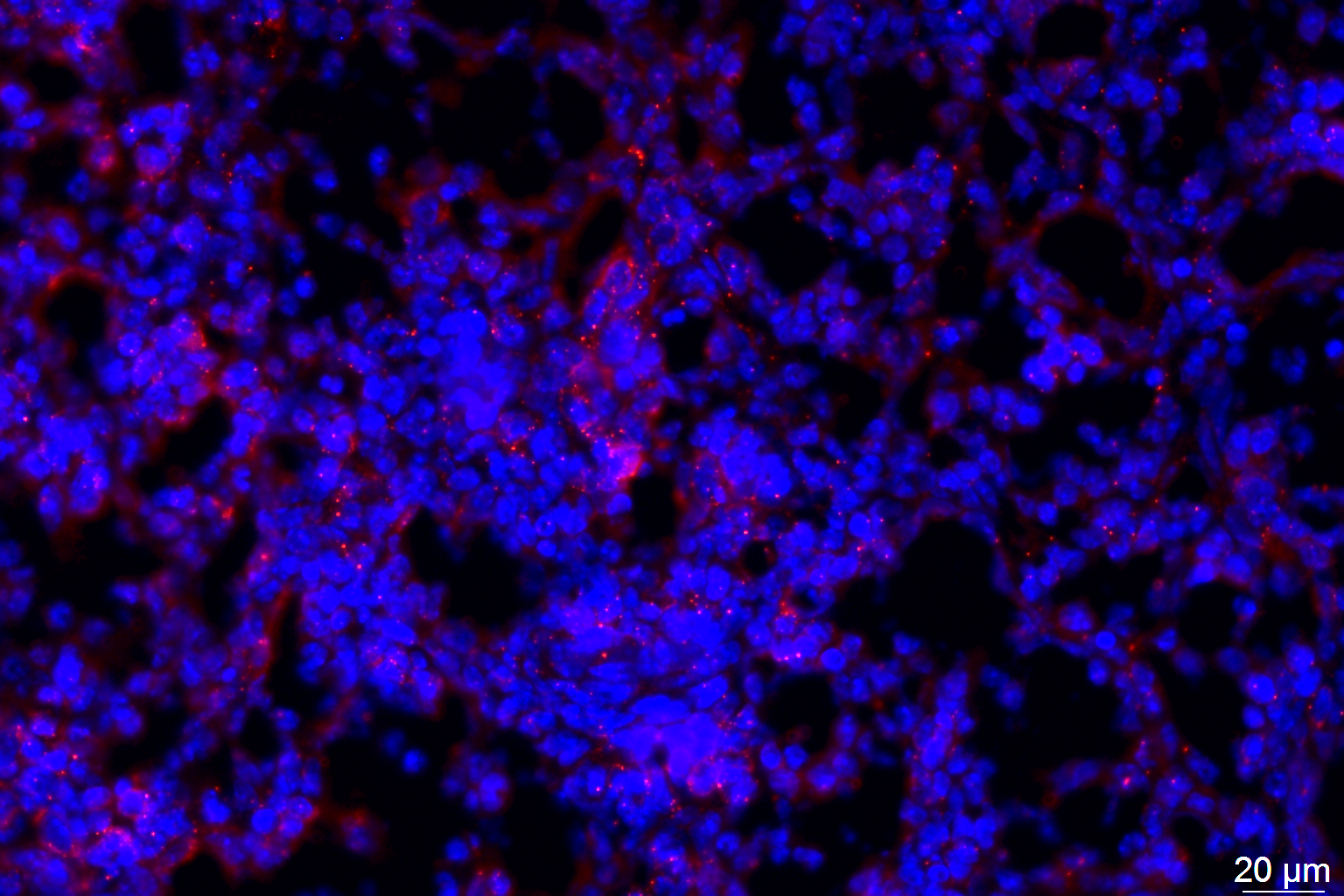

Supplement: Supplementary file 6 — Source data Fig. 4 [file 44318_2026_757_MOESM6_ESM.zip › Figure 4/Figure 4I/IF 2096 KI CD19 DAPI overlay.tiff]

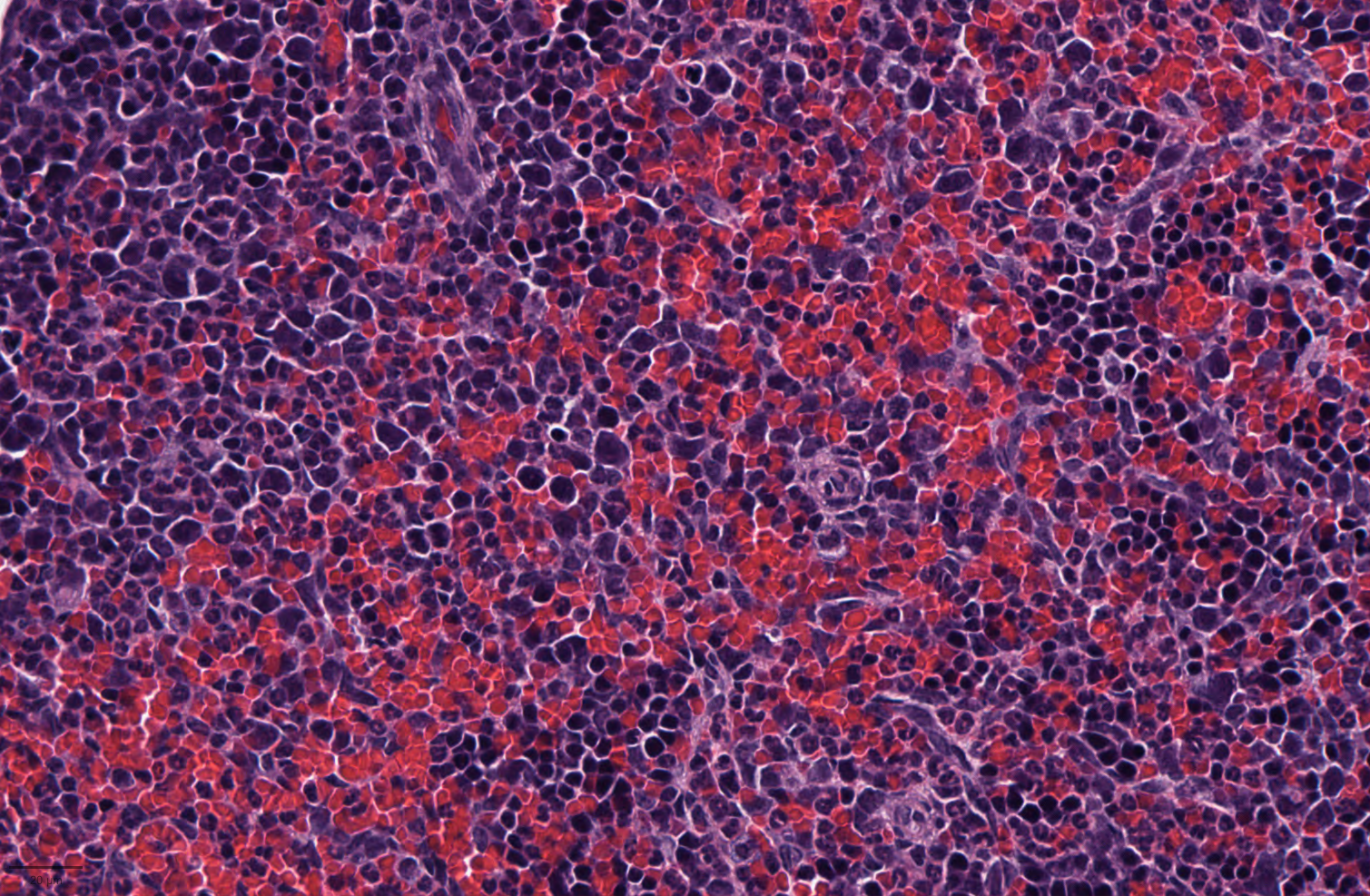

Supplement: Supplementary file 6 — Source data Fig. 4 [file 44318_2026_757_MOESM6_ESM.zip › Figure 4/Figure 4I/HE 1488 KI Spleen-40x.png]

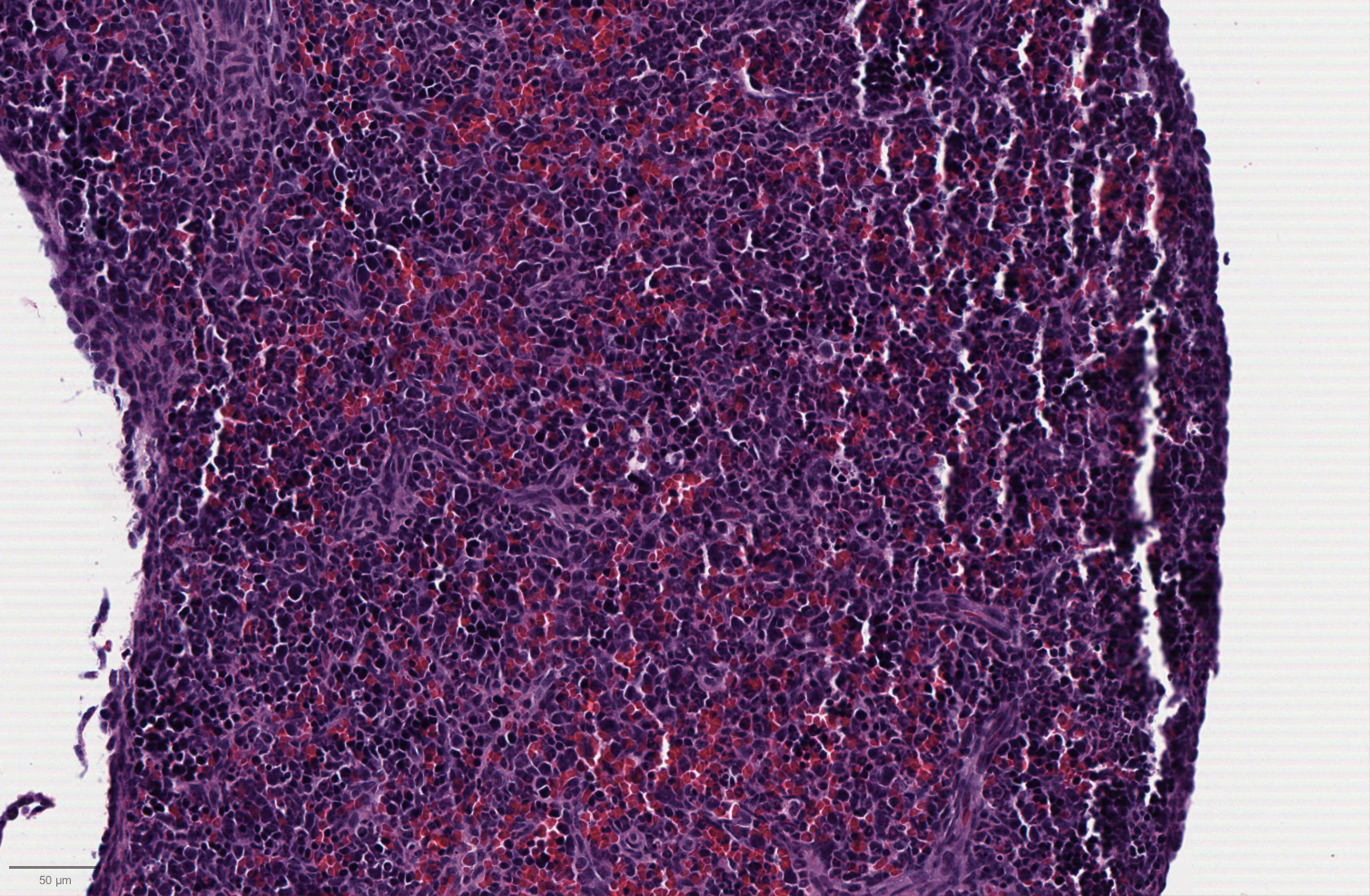

Supplement: Supplementary file 6 — Source data Fig. 4 [file 44318_2026_757_MOESM6_ESM.zip › Figure 4/Figure 4I/HE 1486 WT Spleen-20x.png]

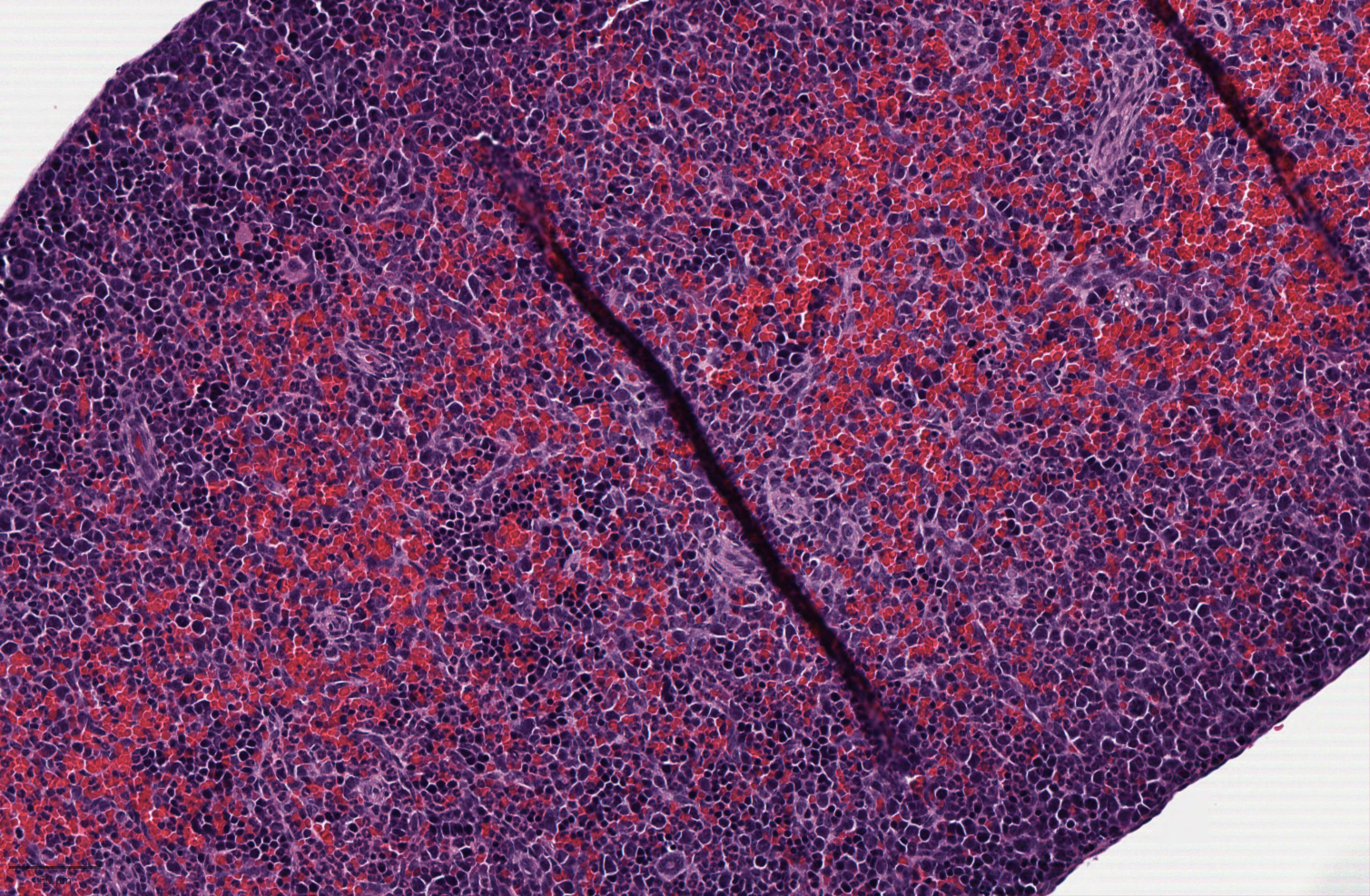

Supplement: Supplementary file 6 — Source data Fig. 4 [file 44318_2026_757_MOESM6_ESM.zip › Figure 4/Figure 4I/HE 1488 KI Spleen-20x.png]

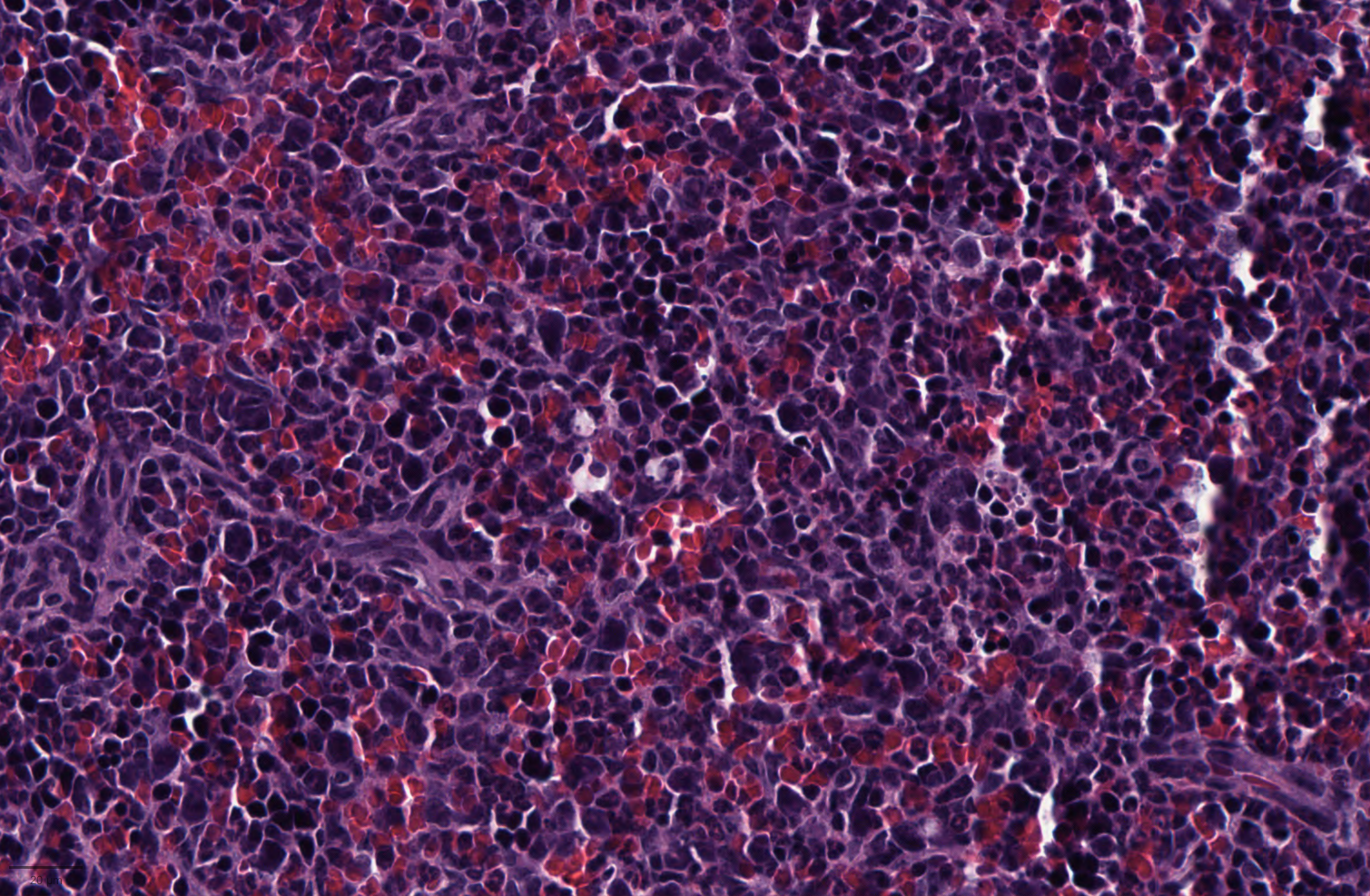

Supplement: Supplementary file 6 — Source data Fig. 4 [file 44318_2026_757_MOESM6_ESM.zip › Figure 4/Figure 4I/HE 1486 WT Spleen-40x.png]

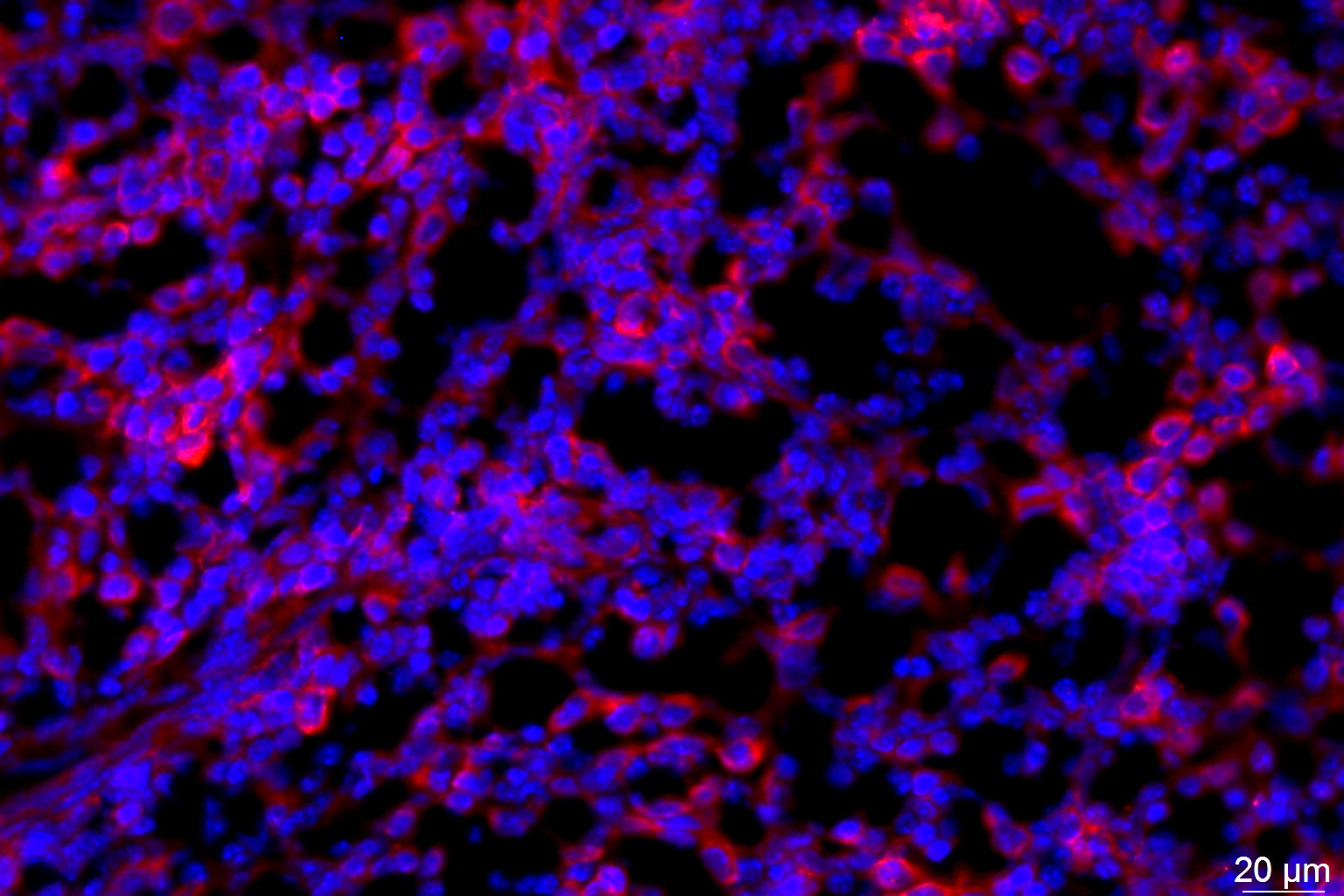

Supplement: Supplementary file 6 — Source data Fig. 4 [file 44318_2026_757_MOESM6_ESM.zip › Figure 4/Figure 4I/IF 2097 WT CD19 DAPI overlay.tiff]

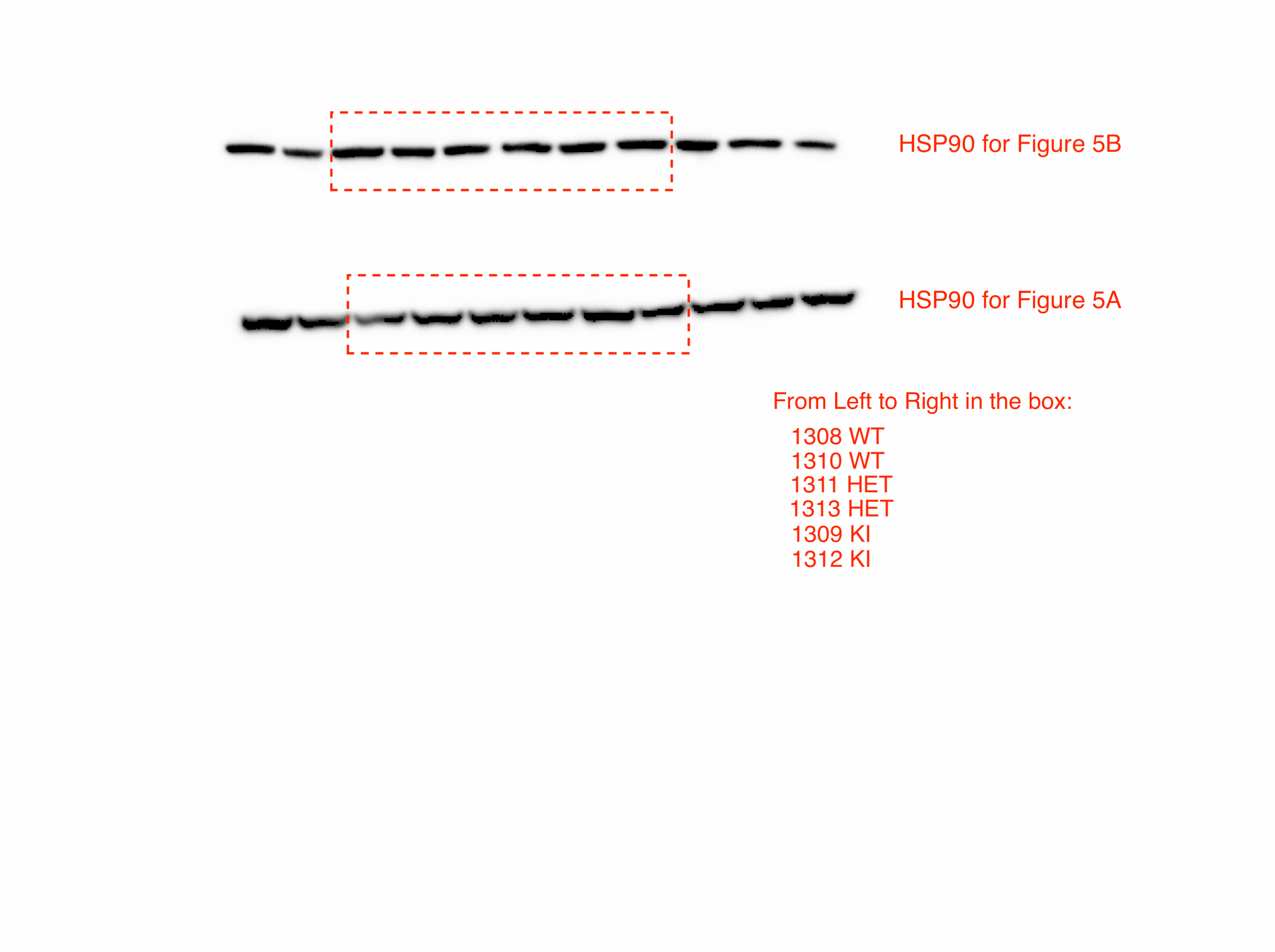

Supplement: Supplementary file 7 — Source data Fig. 5 [file 44318_2026_757_MOESM7_ESM.zip › Figure 5 without 5H/Figure 5A/WB Line B Brain HSP90 no marker.tif]

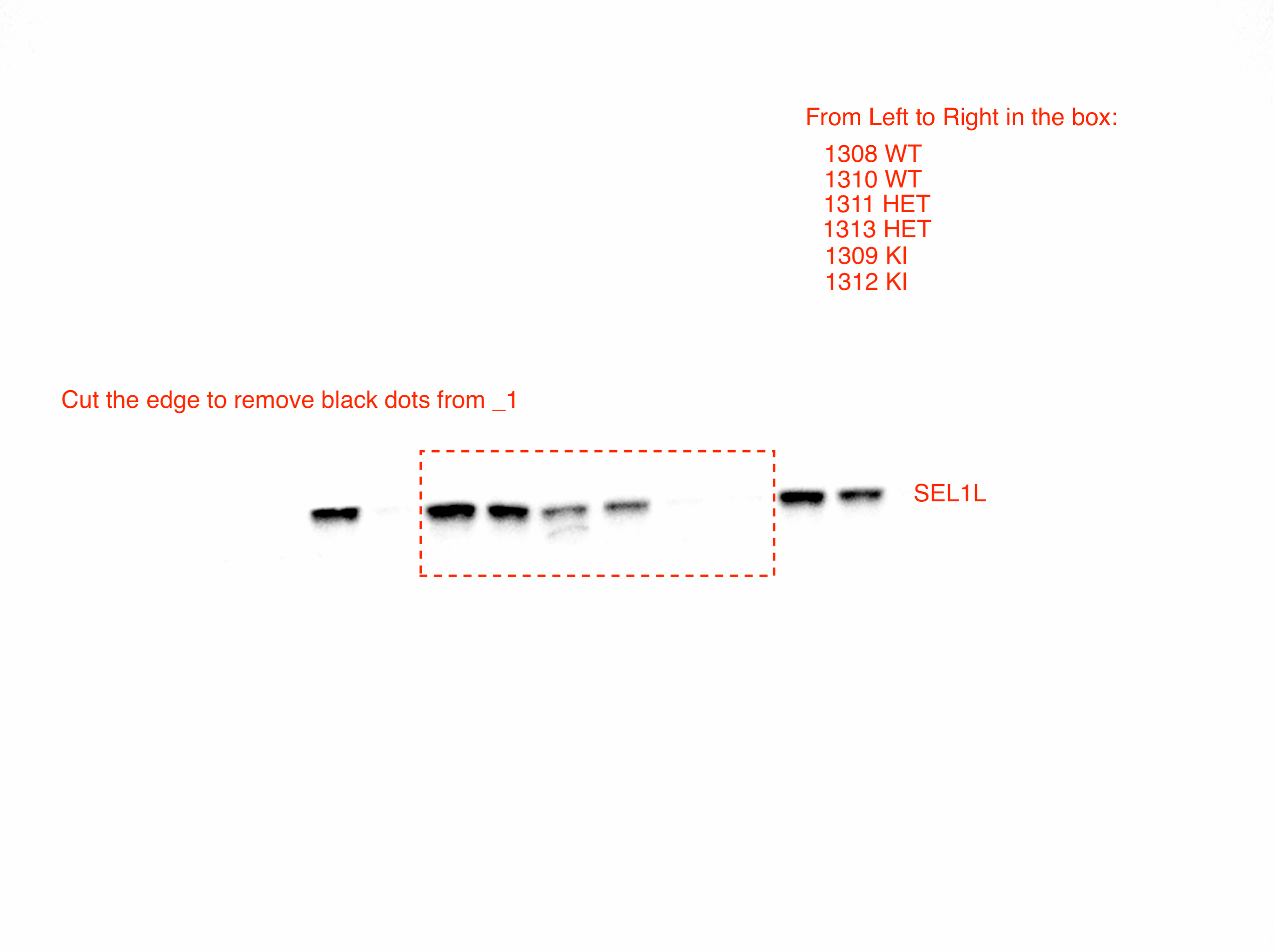

Supplement: Supplementary file 7 — Source data Fig. 5 [file 44318_2026_757_MOESM7_ESM.zip › Figure 5 without 5H/Figure 5A/WB Line B Brain SEL1L no marker_2.tif]

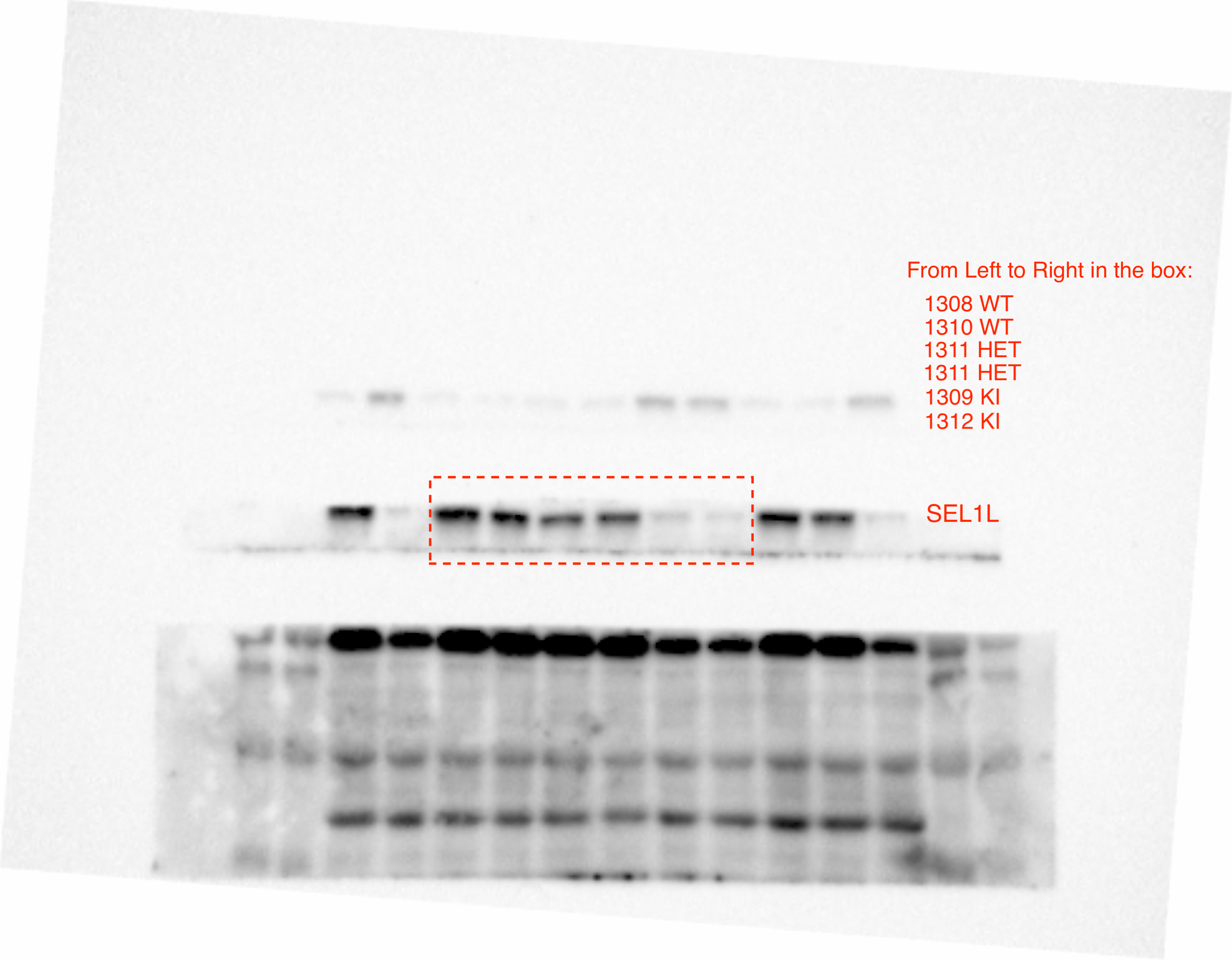

Supplement: Supplementary file 7 — Source data Fig. 5 [file 44318_2026_757_MOESM7_ESM.zip › Figure 5 without 5H/Figure 5A/WB Line B Brain SEL1L no marker_1.tif]

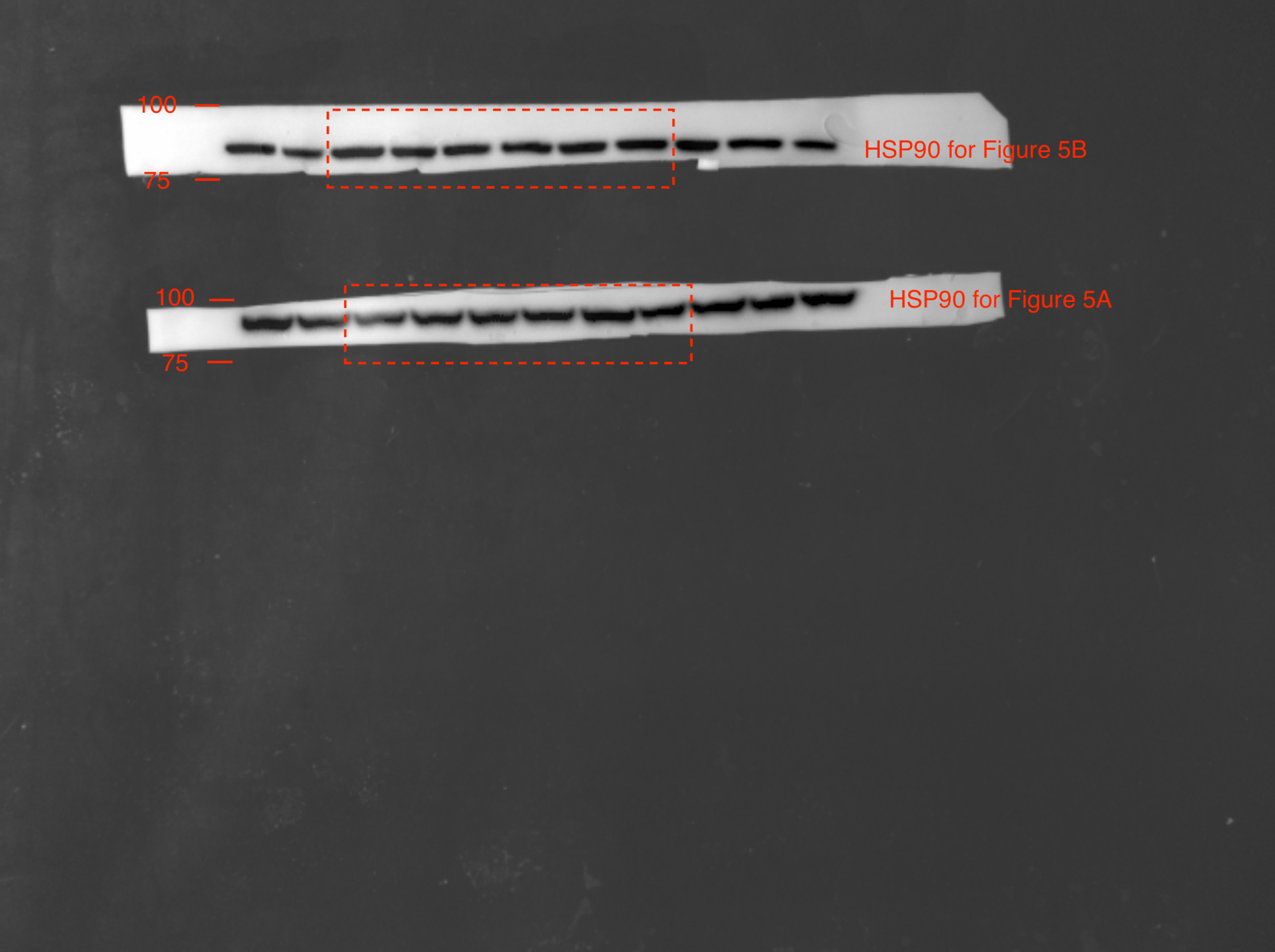

Supplement: Supplementary file 7 — Source data Fig. 5 [file 44318_2026_757_MOESM7_ESM.zip › Figure 5 without 5H/Figure 5A/WB Line B Brain HSP90 merged with marker.tif]

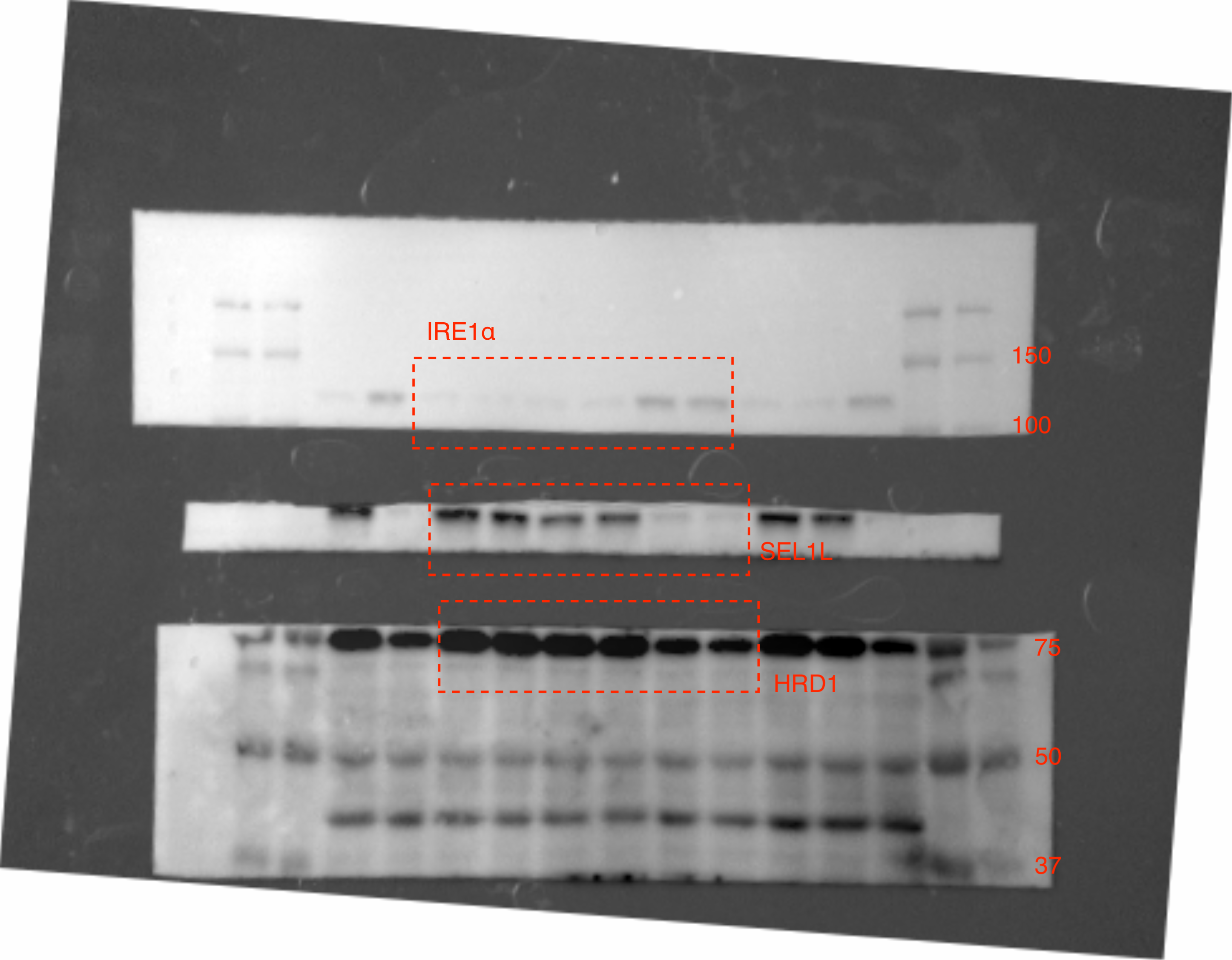

Supplement: Supplementary file 7 — Source data Fig. 5 [file 44318_2026_757_MOESM7_ESM.zip › Figure 5 without 5H/Figure 5A/WB Line B Brain SEL1L merged with marker.tif]

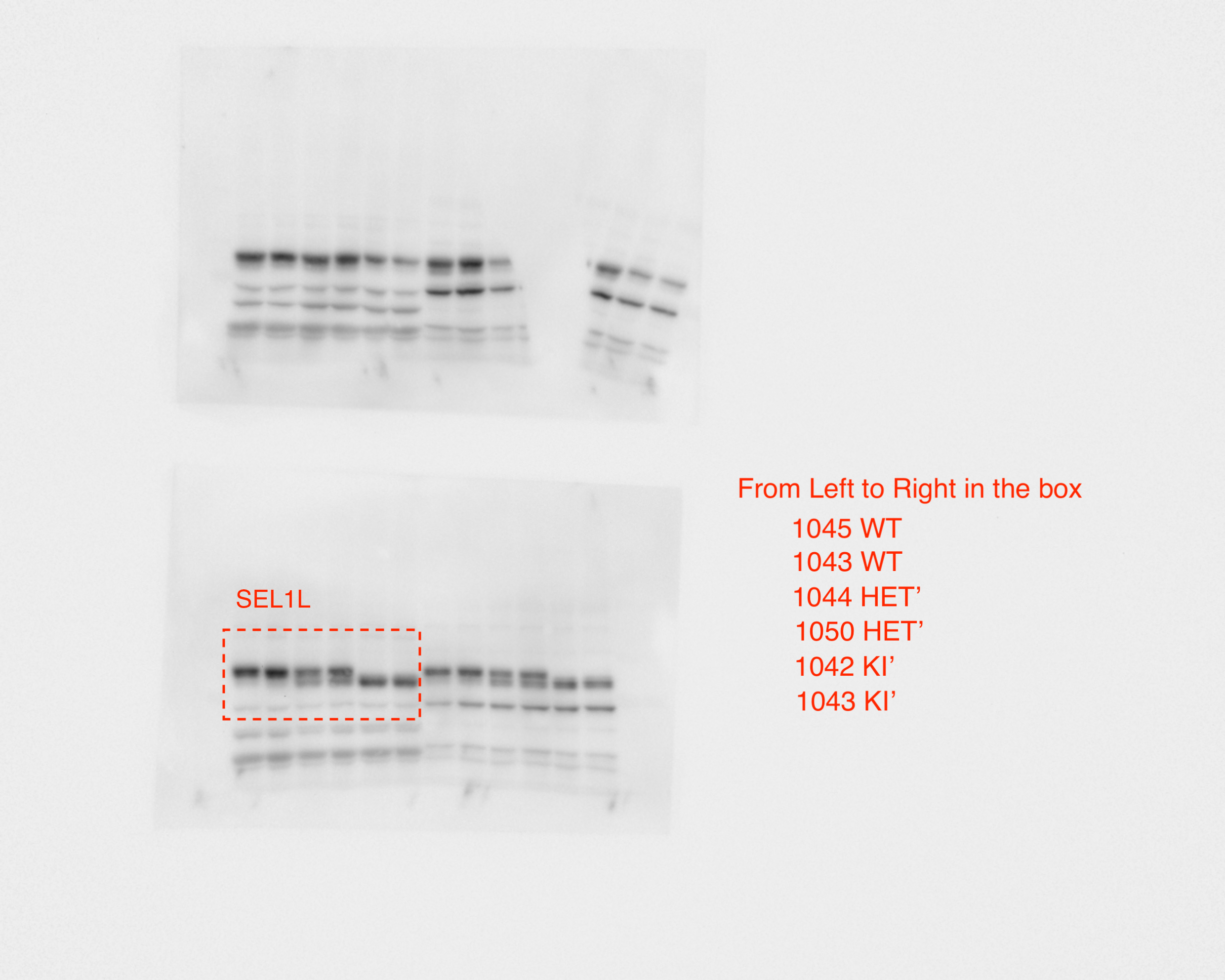

Supplement: Supplementary file 7 — Source data Fig. 5 [file 44318_2026_757_MOESM7_ESM.zip › Figure 5 without 5H/Figure 5A/WB Line C Cortex SEL1L no marker.tif]

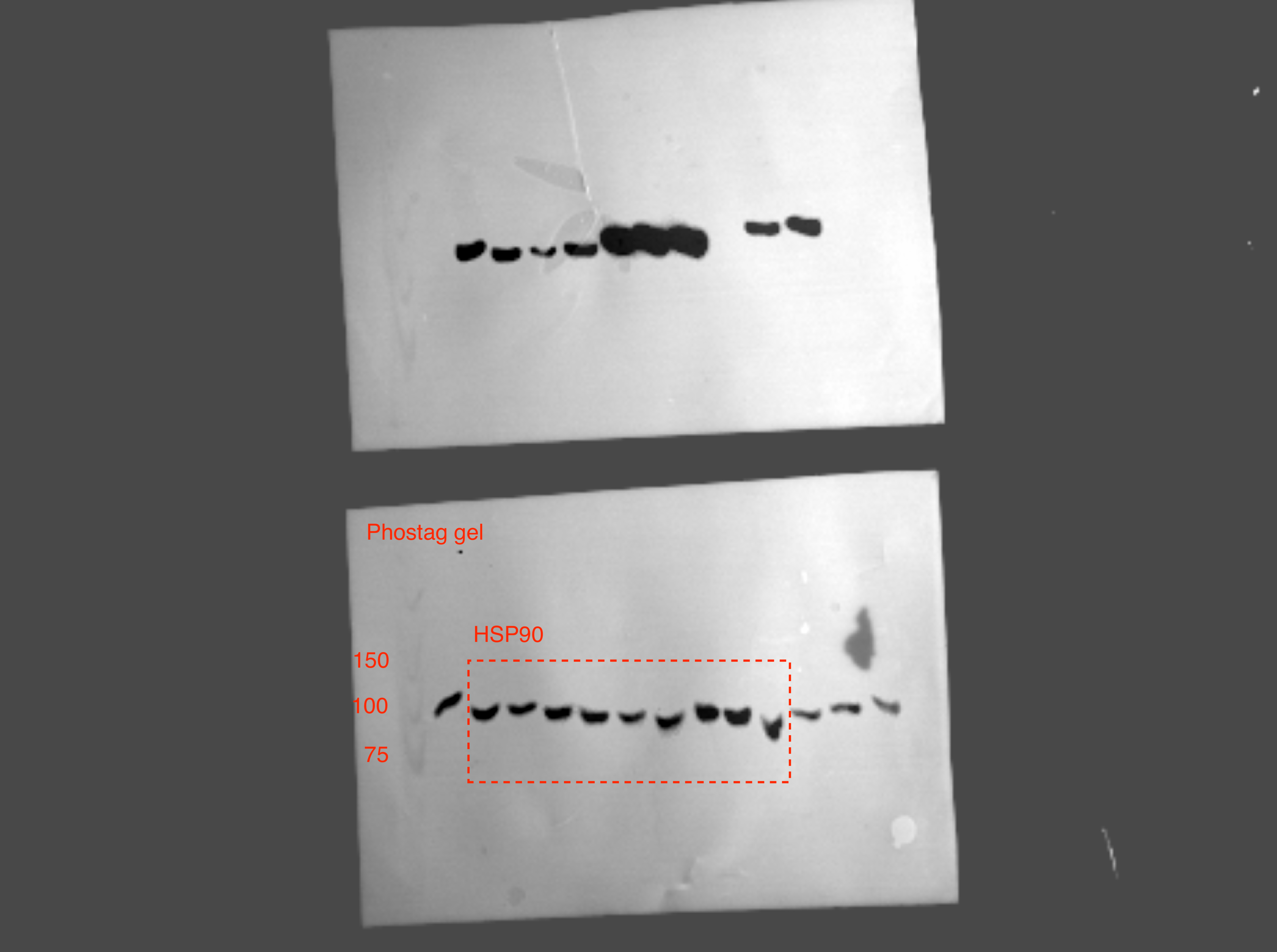

Supplement: Supplementary file 7 — Source data Fig. 5 [file 44318_2026_757_MOESM7_ESM.zip › Figure 5 without 5H/Figure 5F/WB phostag HSP90 merged with marker.tif]

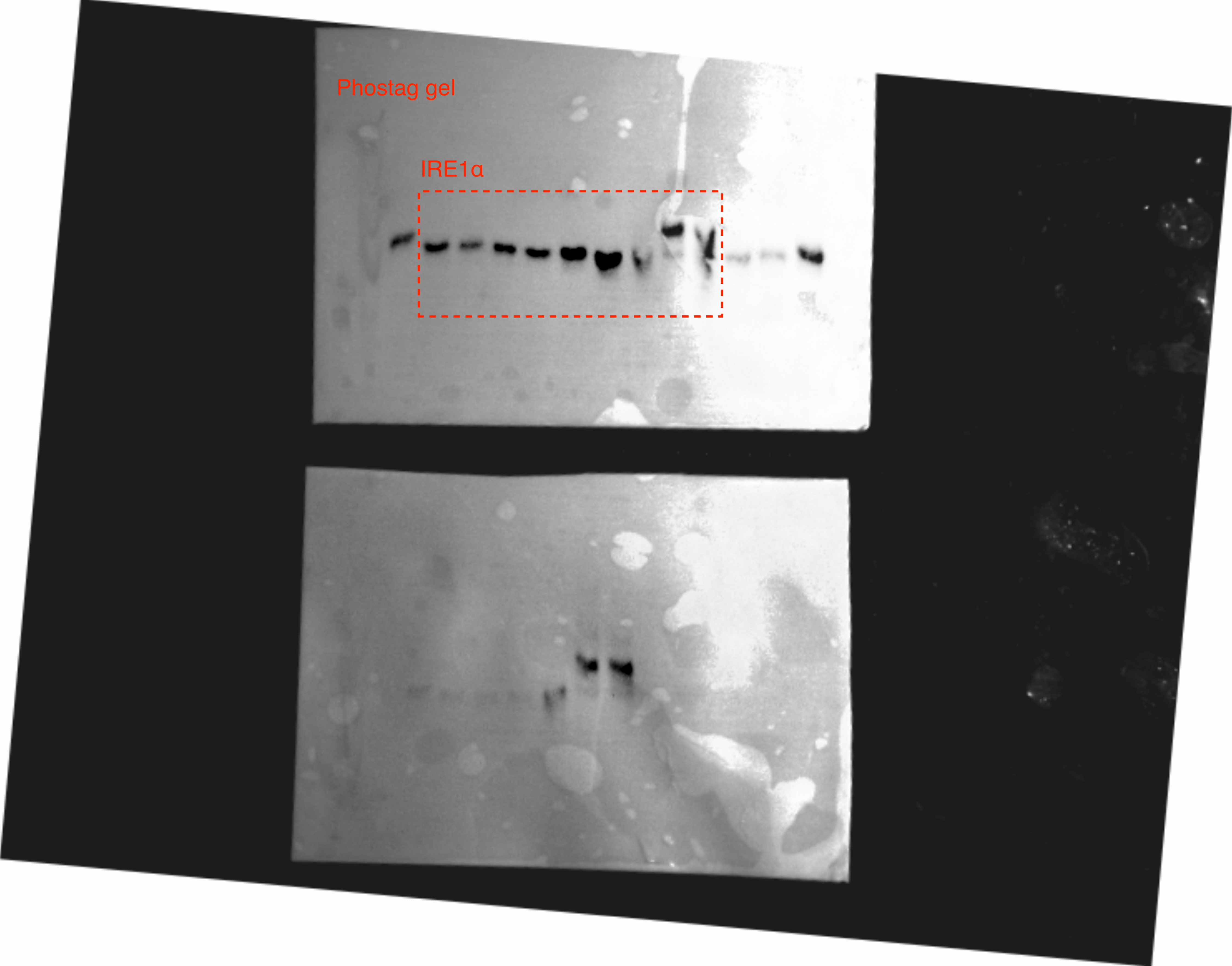

Supplement: Supplementary file 7 — Source data Fig. 5 [file 44318_2026_757_MOESM7_ESM.zip › Figure 5 without 5H/Figure 5F/WB phostag IRE1╬▒ merged with marker.tif]

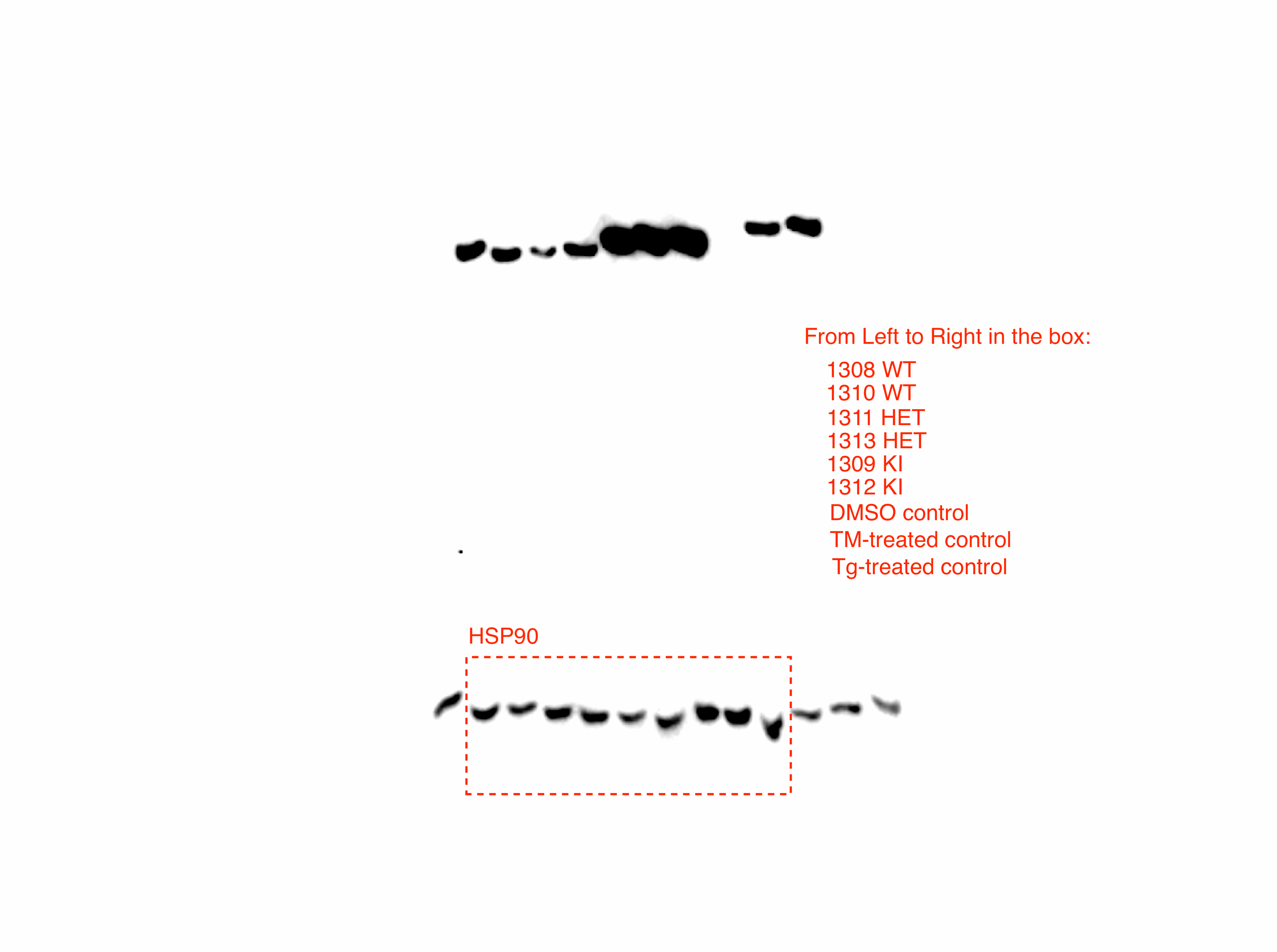

Supplement: Supplementary file 7 — Source data Fig. 5 [file 44318_2026_757_MOESM7_ESM.zip › Figure 5 without 5H/Figure 5F/WB phostag HSP90 no marker.tif]

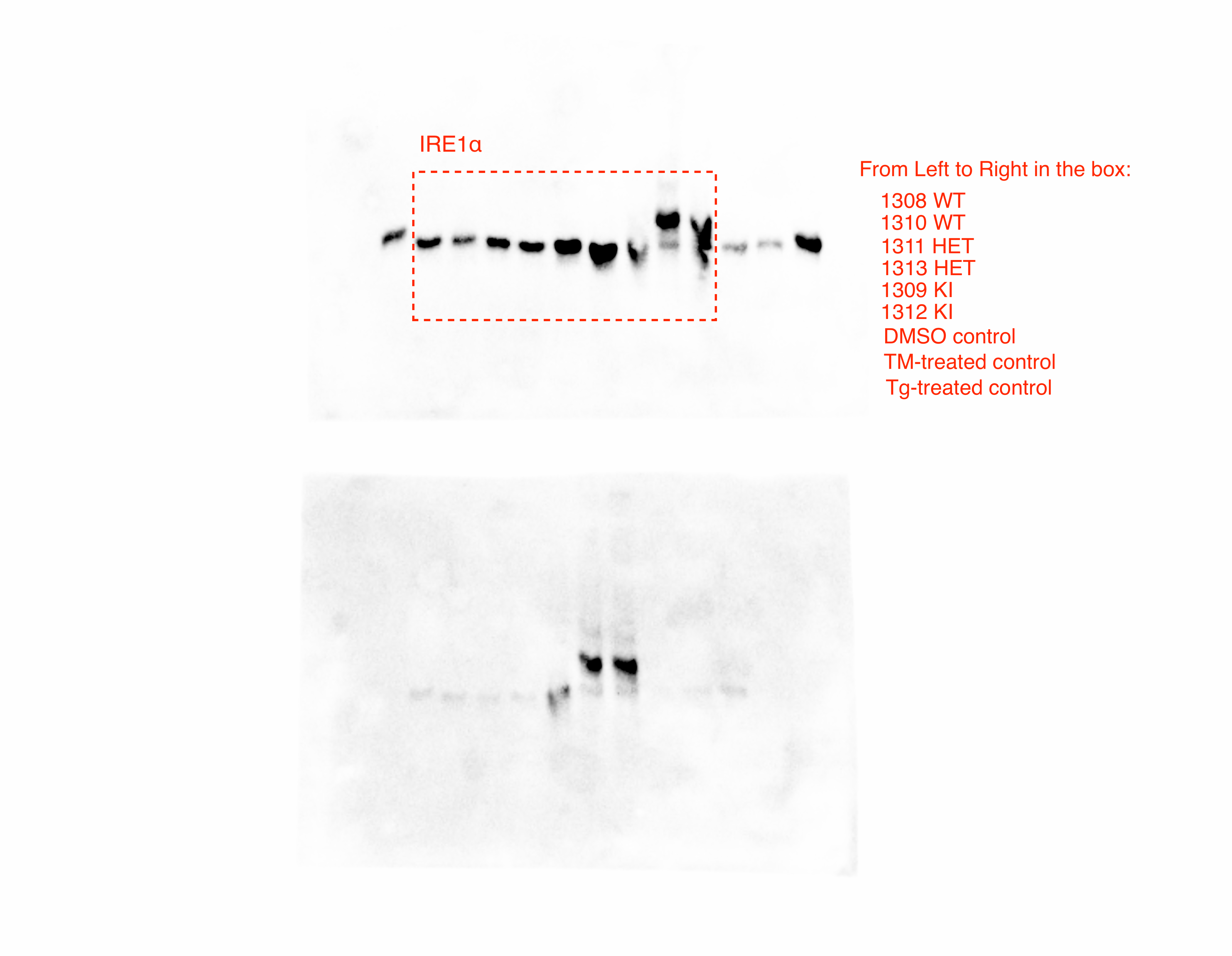

Supplement: Supplementary file 7 — Source data Fig. 5 [file 44318_2026_757_MOESM7_ESM.zip › Figure 5 without 5H/Figure 5F/WB phostag IRE1╬▒ no marker.tif]

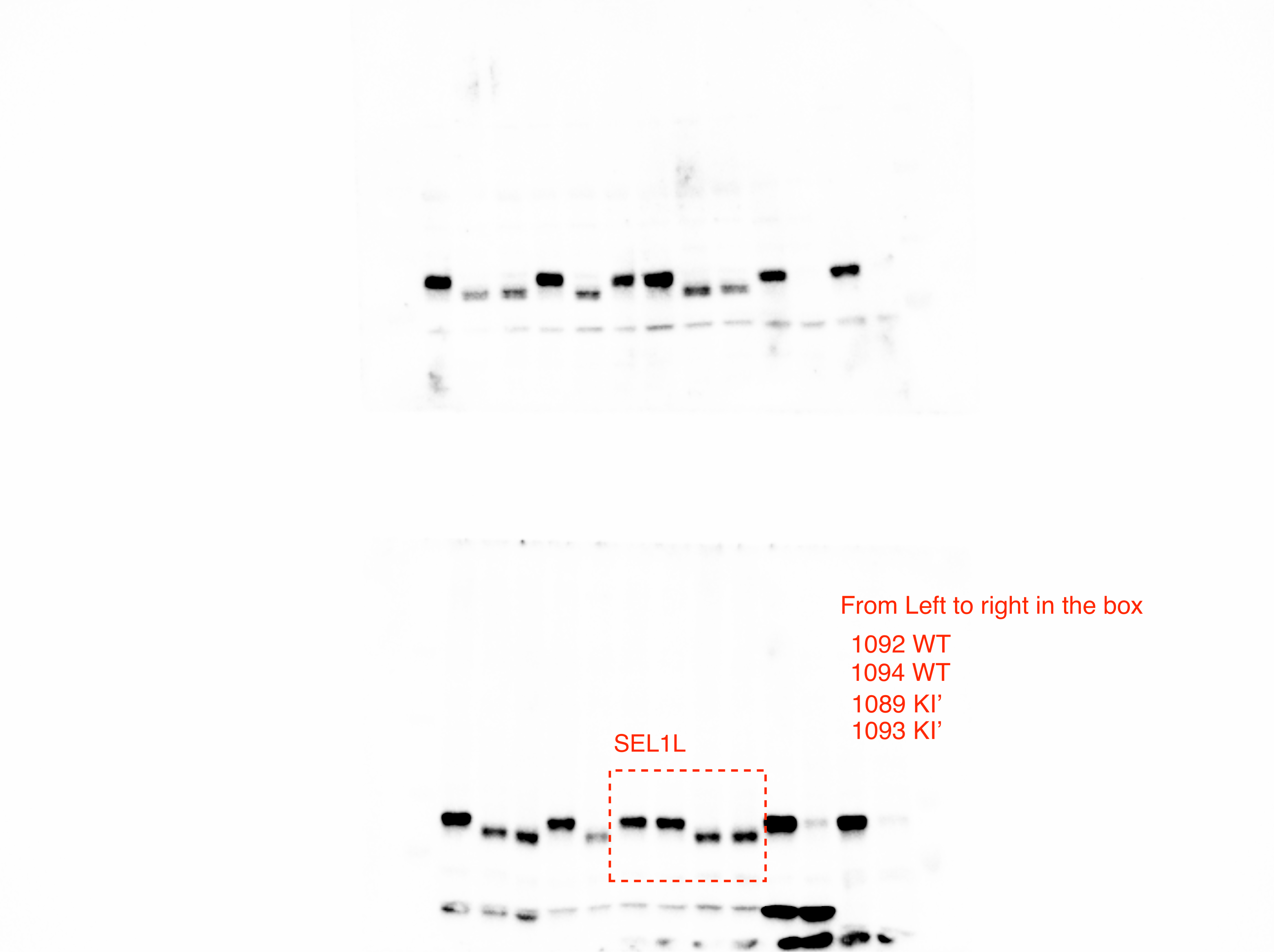

Supplement: Supplementary file 7 — Source data Fig. 5 [file 44318_2026_757_MOESM7_ESM.zip › Figure 5 without 5H/Figure 5D/Line C SEL1L no marker.tif]

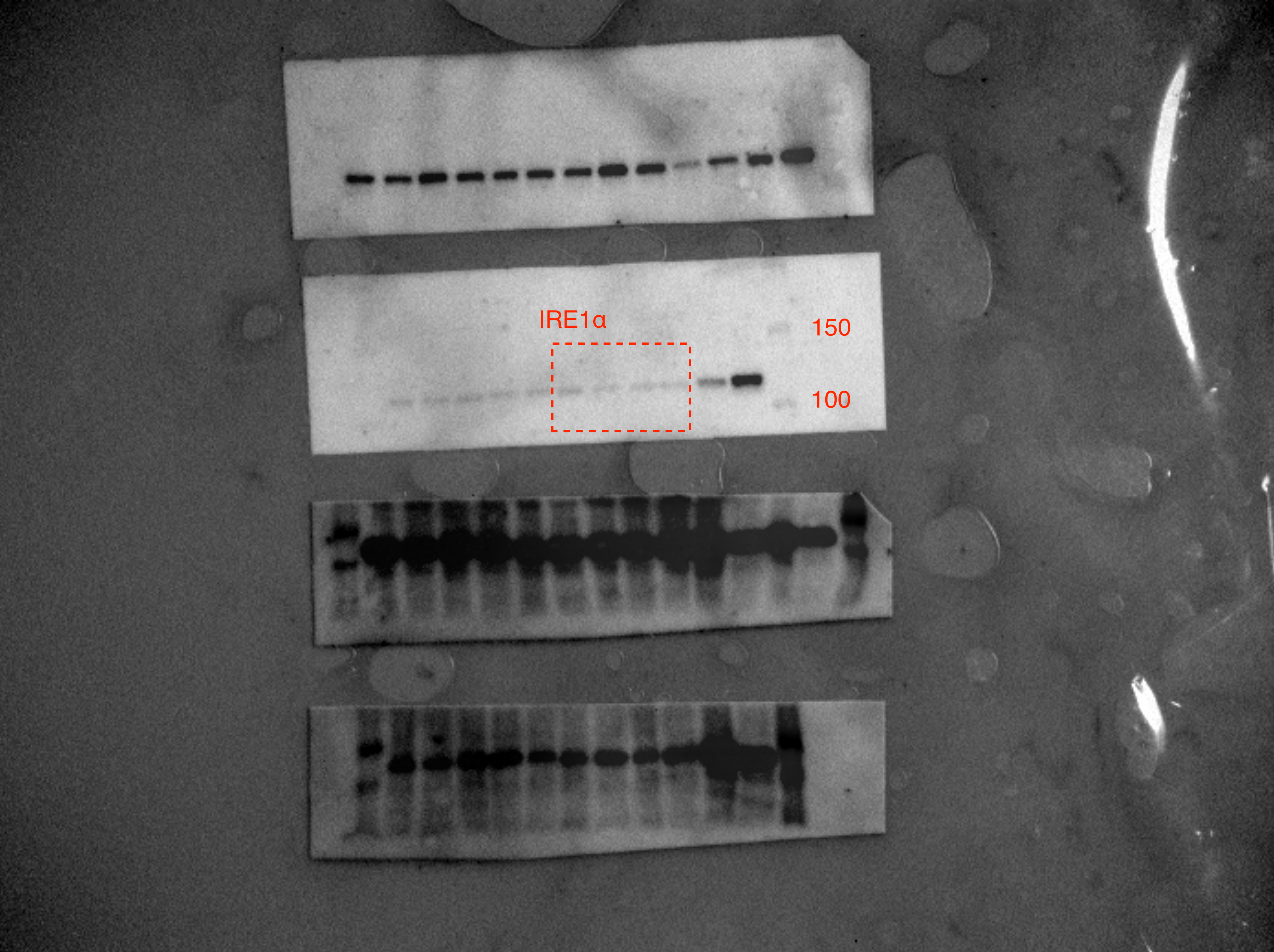

Supplement: Supplementary file 7 — Source data Fig. 5 [file 44318_2026_757_MOESM7_ESM.zip › Figure 5 without 5H/Figure 5D/Line C IRE1a merged with marker.tif]

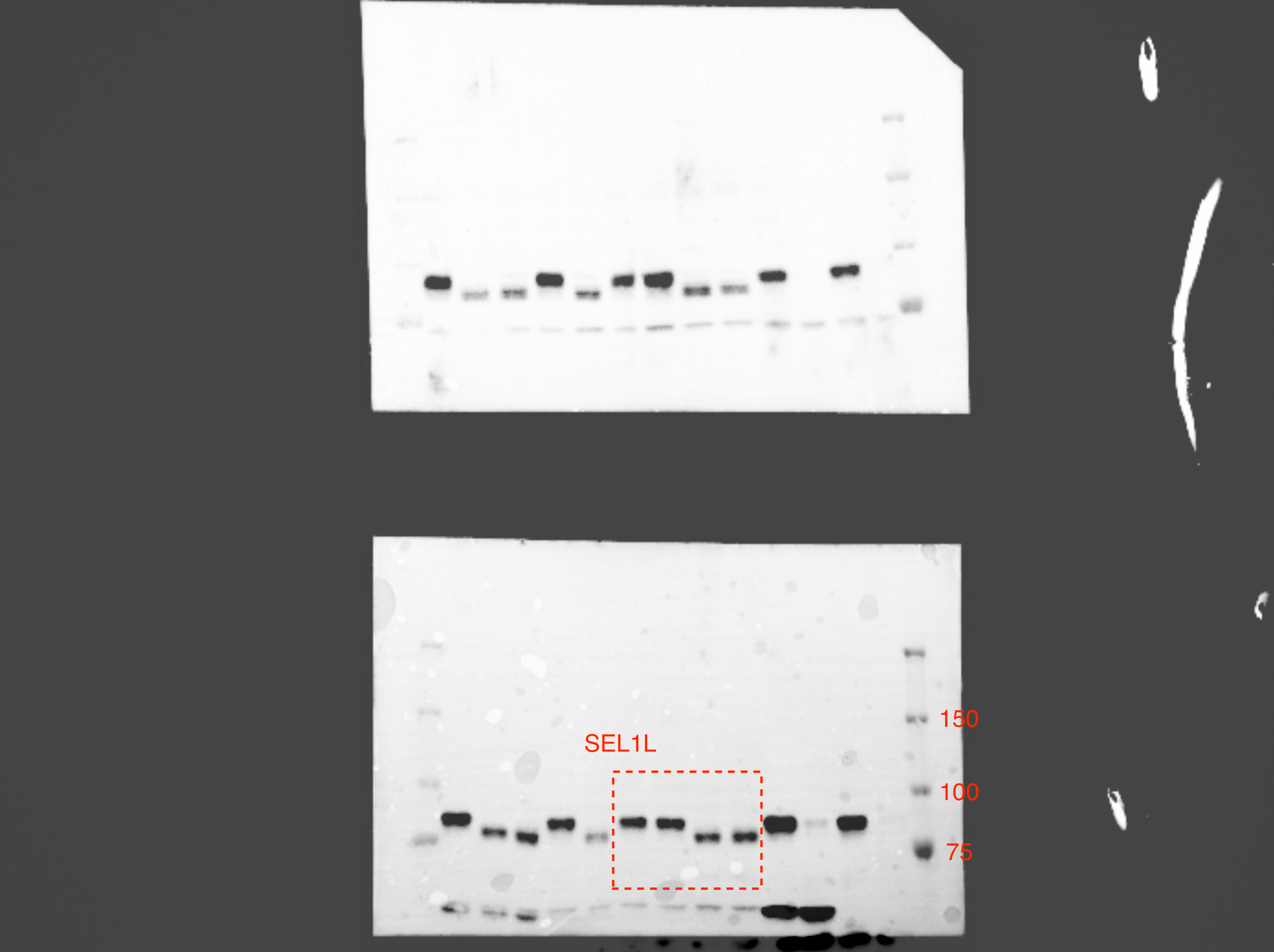

Supplement: Supplementary file 7 — Source data Fig. 5 [file 44318_2026_757_MOESM7_ESM.zip › Figure 5 without 5H/Figure 5D/Line C SEL1L merged with marker.tif]

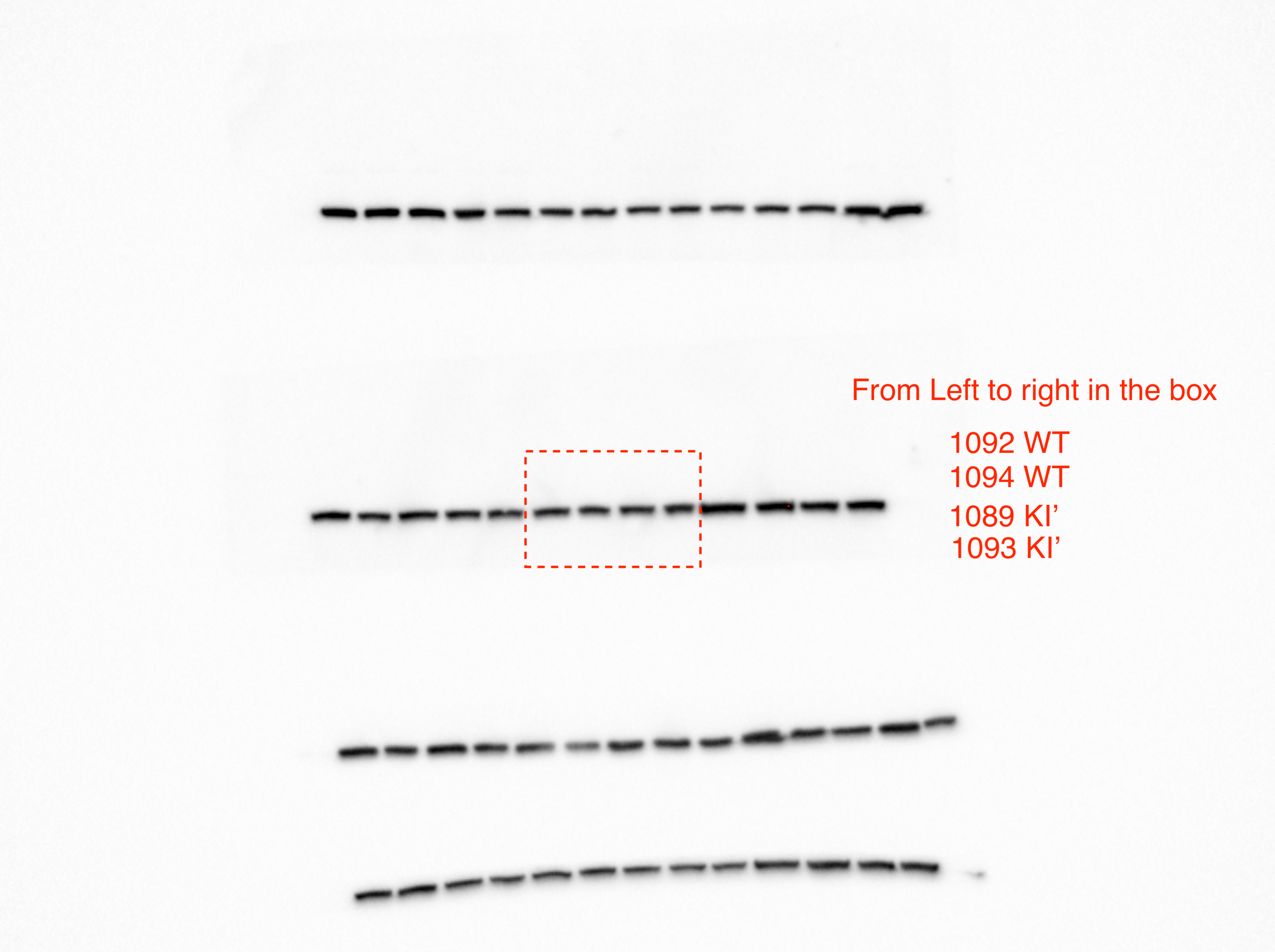

Supplement: Supplementary file 7 — Source data Fig. 5 [file 44318_2026_757_MOESM7_ESM.zip › Figure 5 without 5H/Figure 5D/Line C HSP90 no marker.tif]

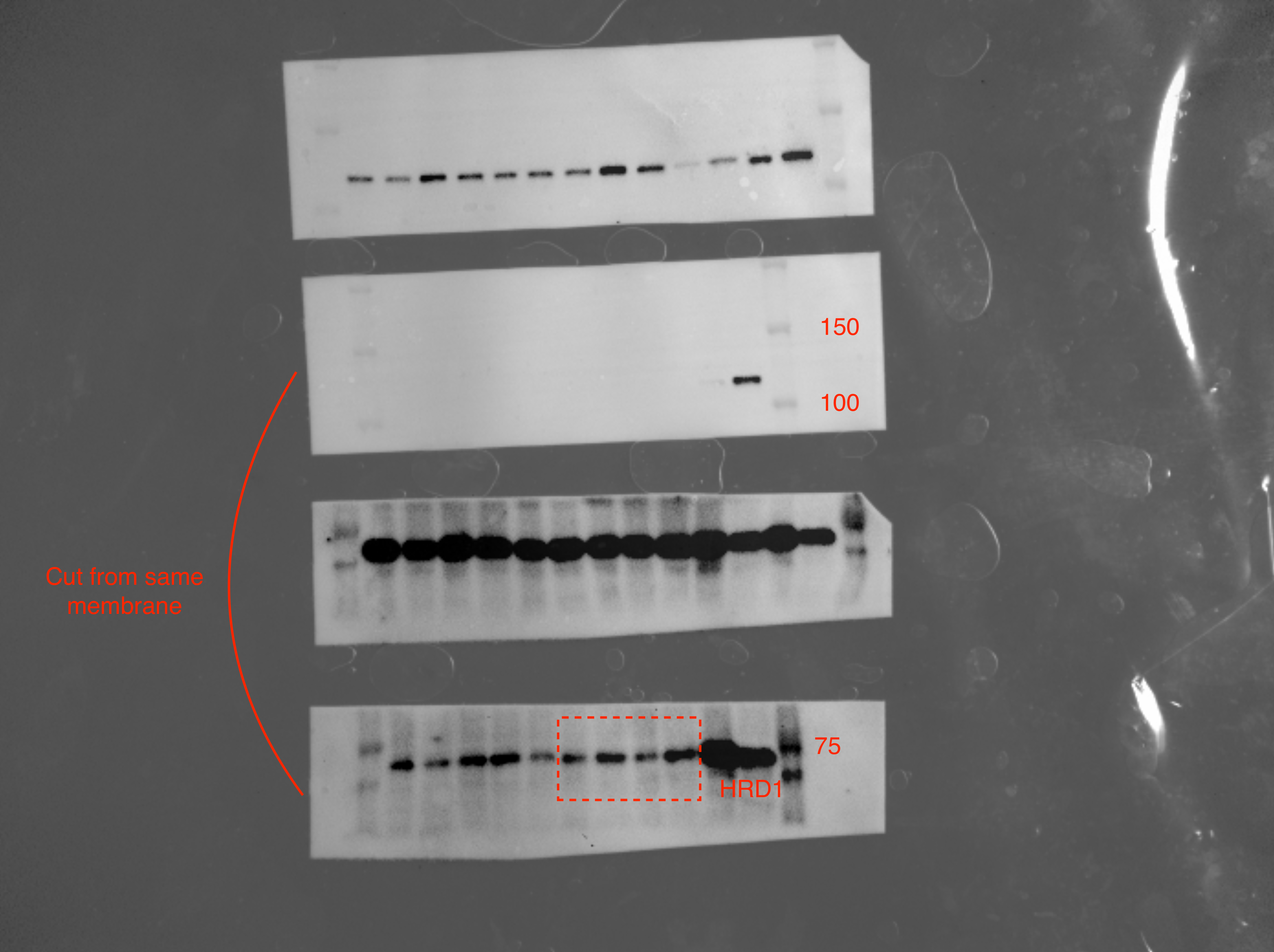

Supplement: Supplementary file 7 — Source data Fig. 5 [file 44318_2026_757_MOESM7_ESM.zip › Figure 5 without 5H/Figure 5D/Line C HRD1 merged with marker.tif]

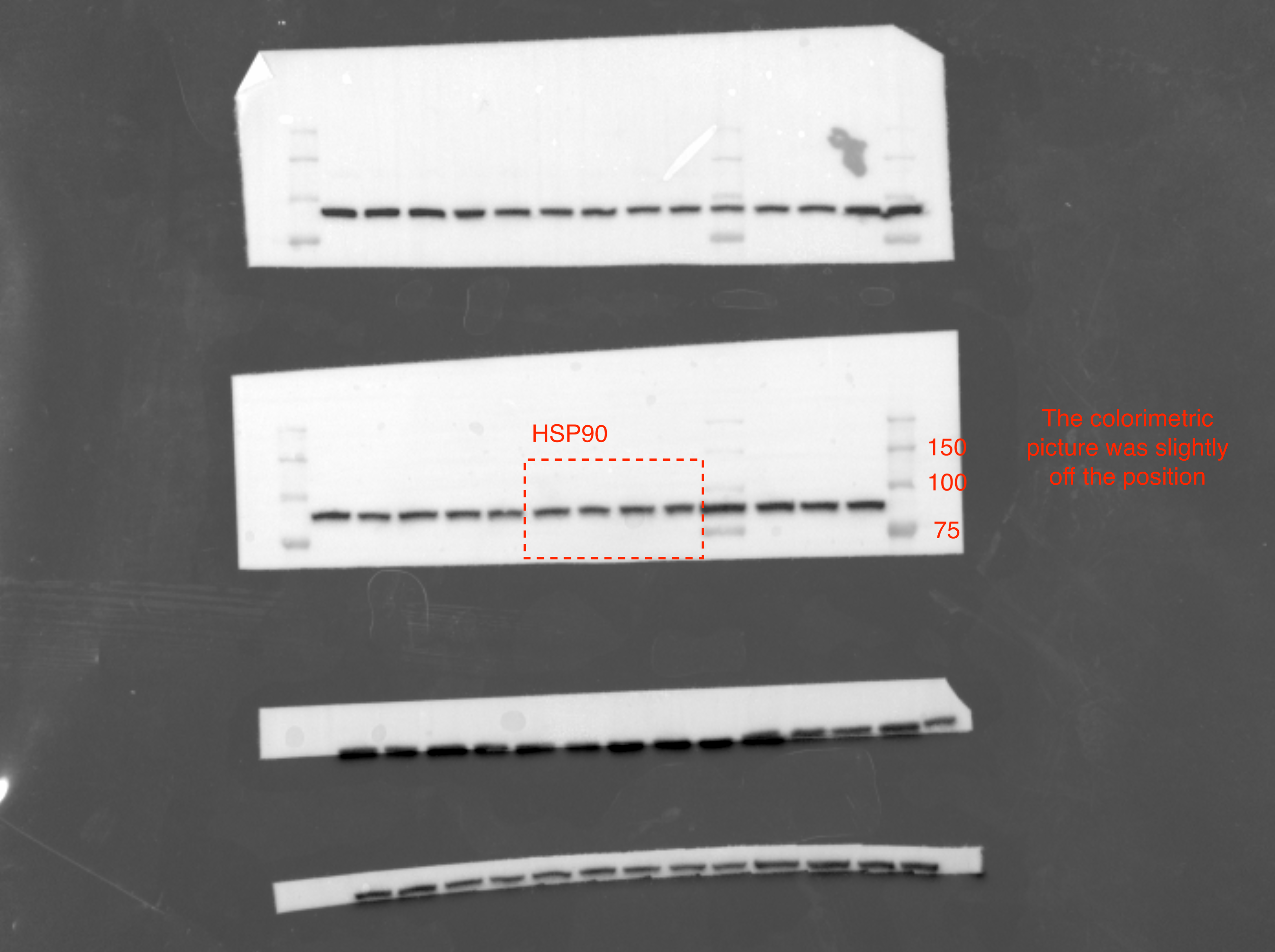

Supplement: Supplementary file 7 — Source data Fig. 5 [file 44318_2026_757_MOESM7_ESM.zip › Figure 5 without 5H/Figure 5D/Line C HSP90 merged with marker.tif]

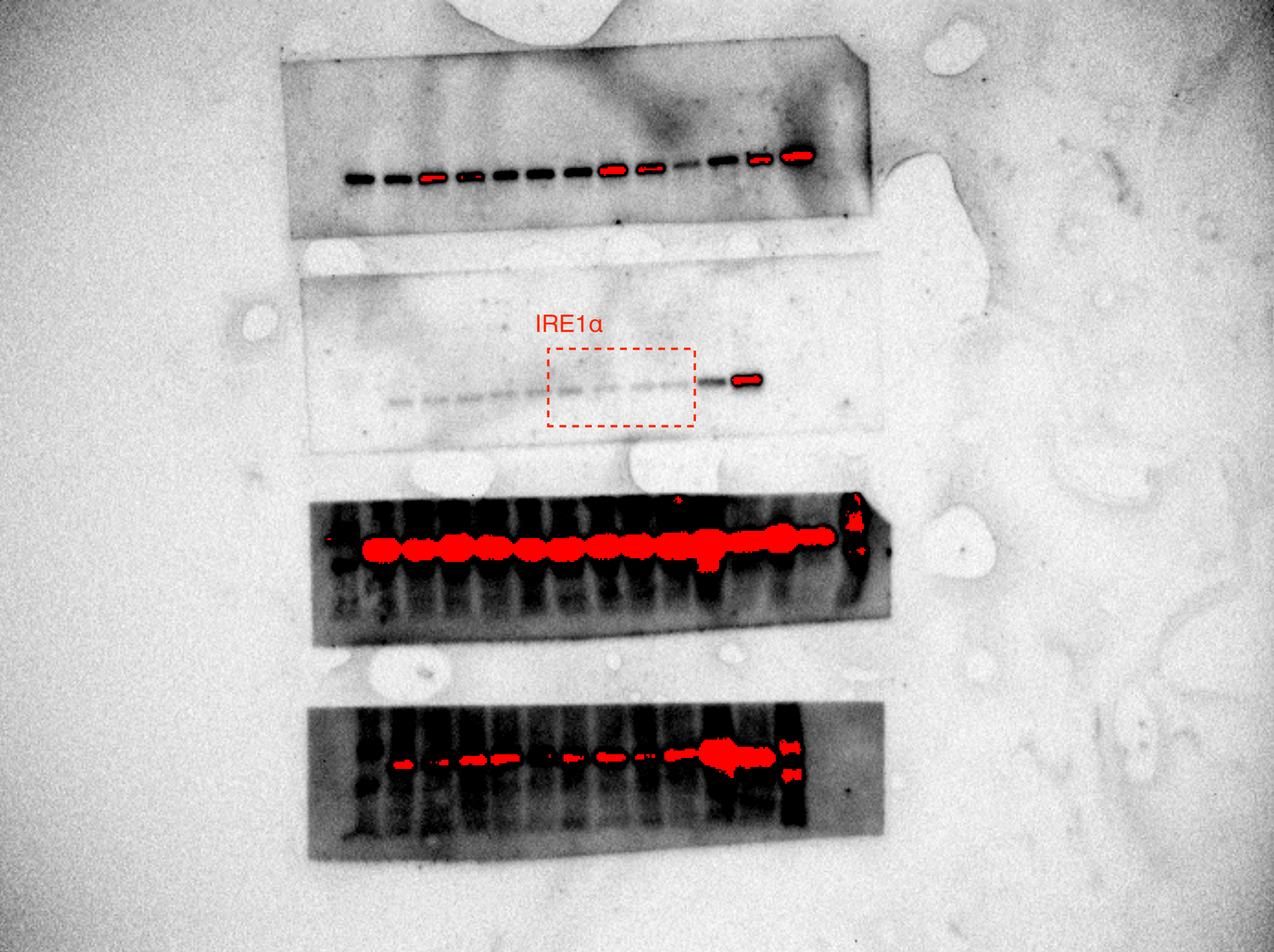

Supplement: Supplementary file 7 — Source data Fig. 5 [file 44318_2026_757_MOESM7_ESM.zip › Figure 5 without 5H/Figure 5D/Line C IRE1a no marker.tif]

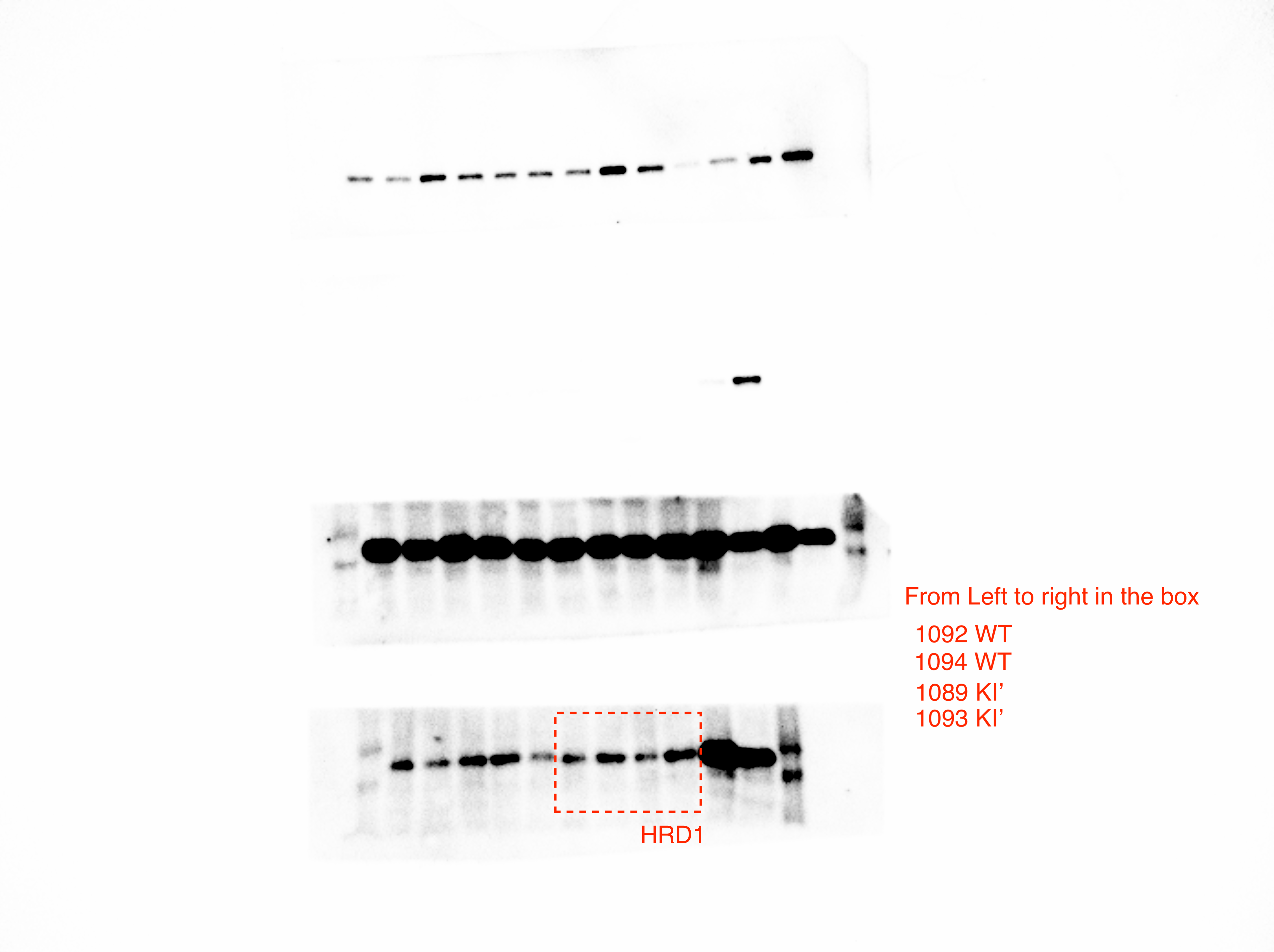

Supplement: Supplementary file 7 — Source data Fig. 5 [file 44318_2026_757_MOESM7_ESM.zip › Figure 5 without 5H/Figure 5D/Line C HRD1 no marker.tif]

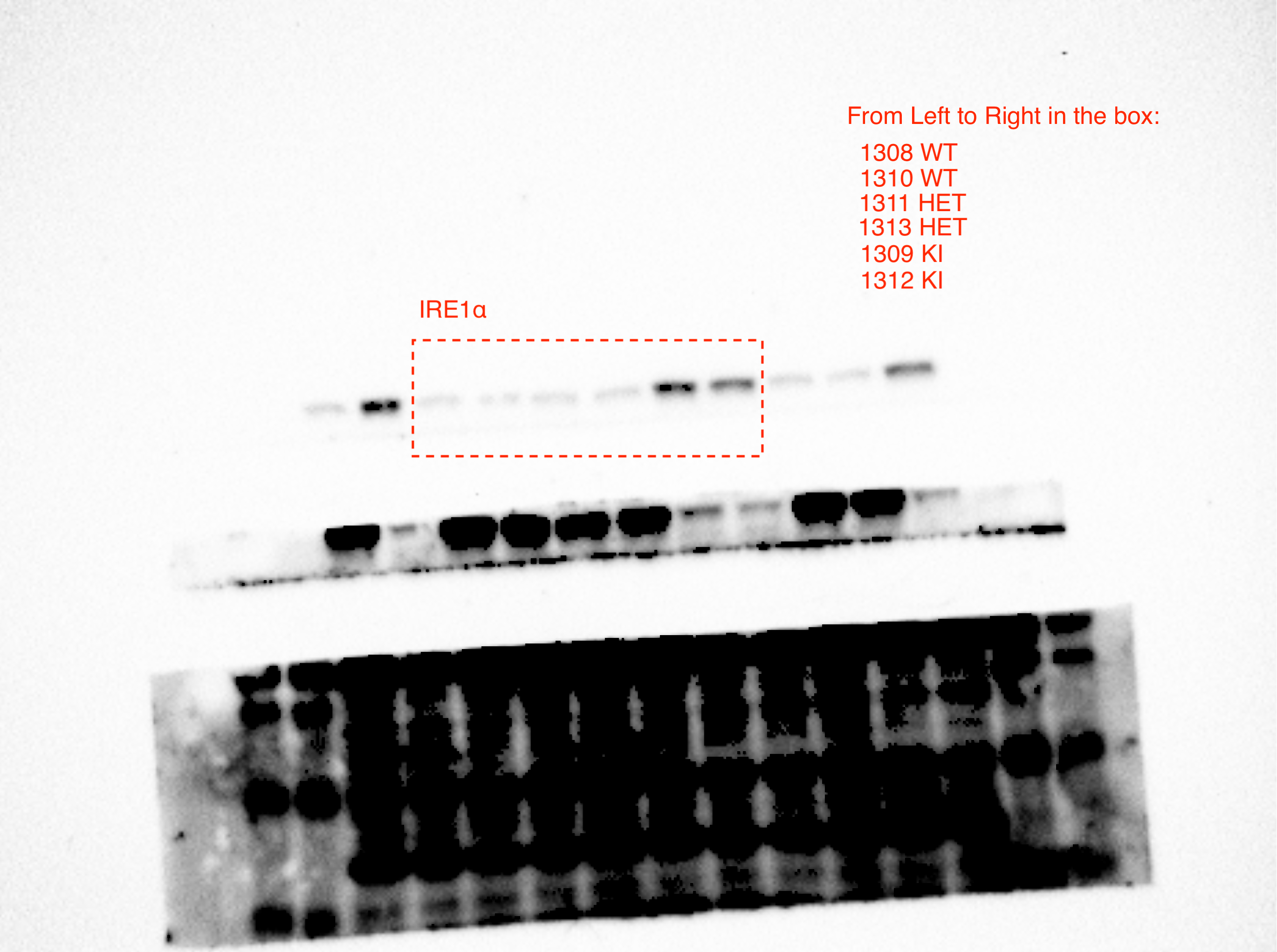

Supplement: Supplementary file 7 — Source data Fig. 5 [file 44318_2026_757_MOESM7_ESM.zip › Figure 5 without 5H/Figure 5B/WB Line B Brain IRE1a no marker.tif]

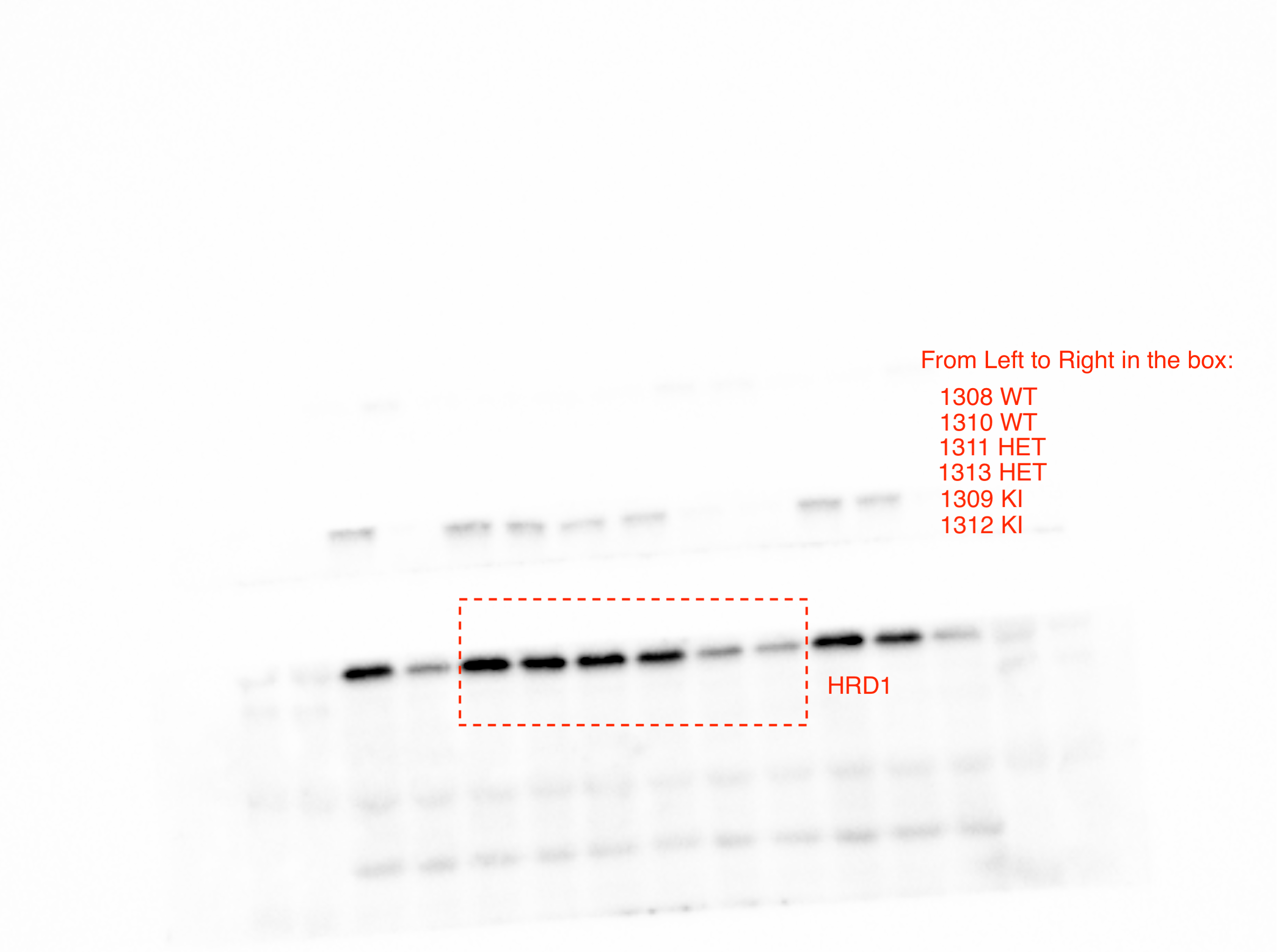

Supplement: Supplementary file 7 — Source data Fig. 5 [file 44318_2026_757_MOESM7_ESM.zip › Figure 5 without 5H/Figure 5B/WB Line B Brain HRD1 no marker.tif]

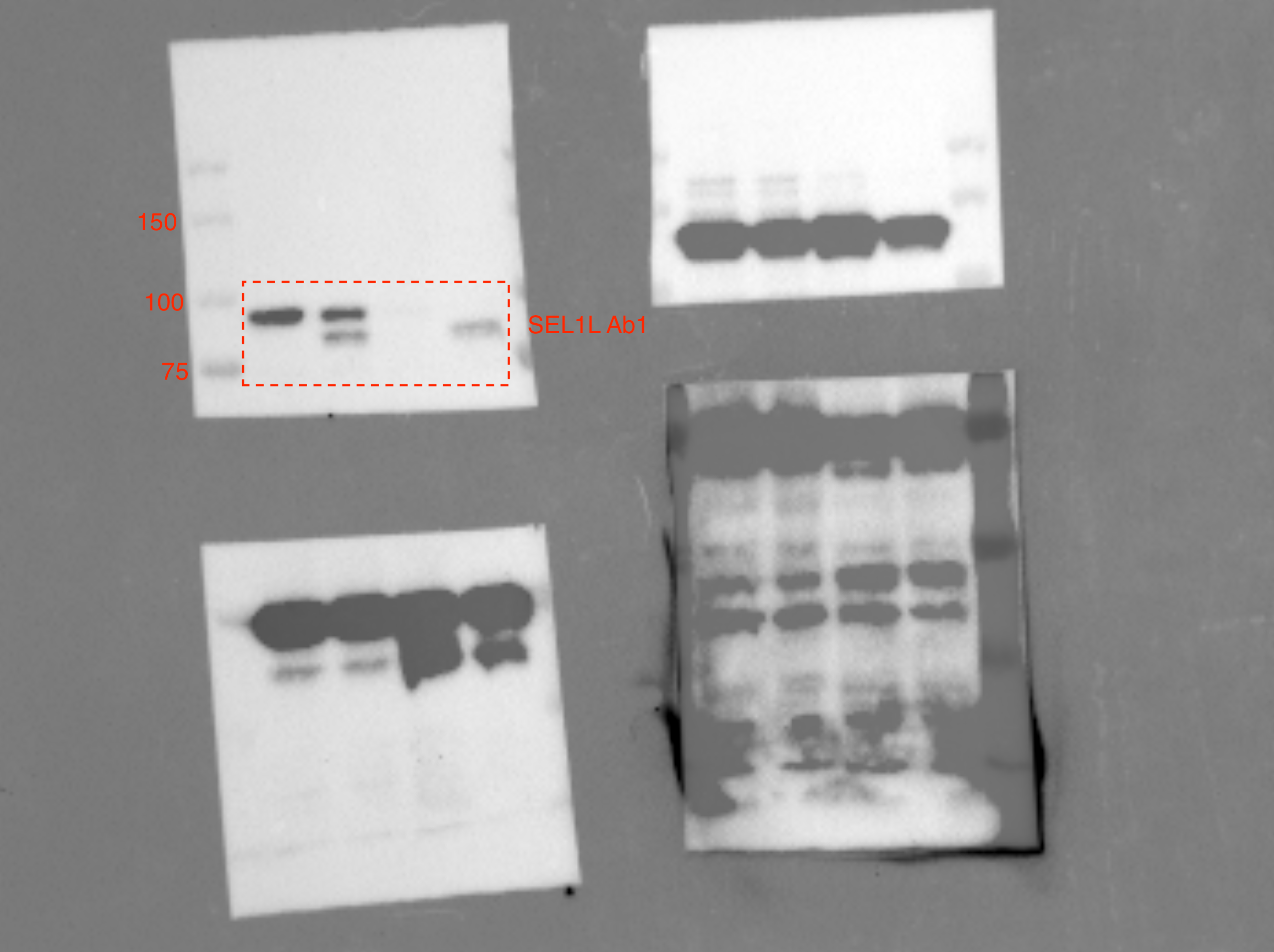

Supplement: Supplementary file 12 — Source data Fig. 7 [file 44318_2026_757_MOESM12_ESM.zip › Figure 7/Figure 7D/WB SEL1L Ab1 merged with marker.tif]

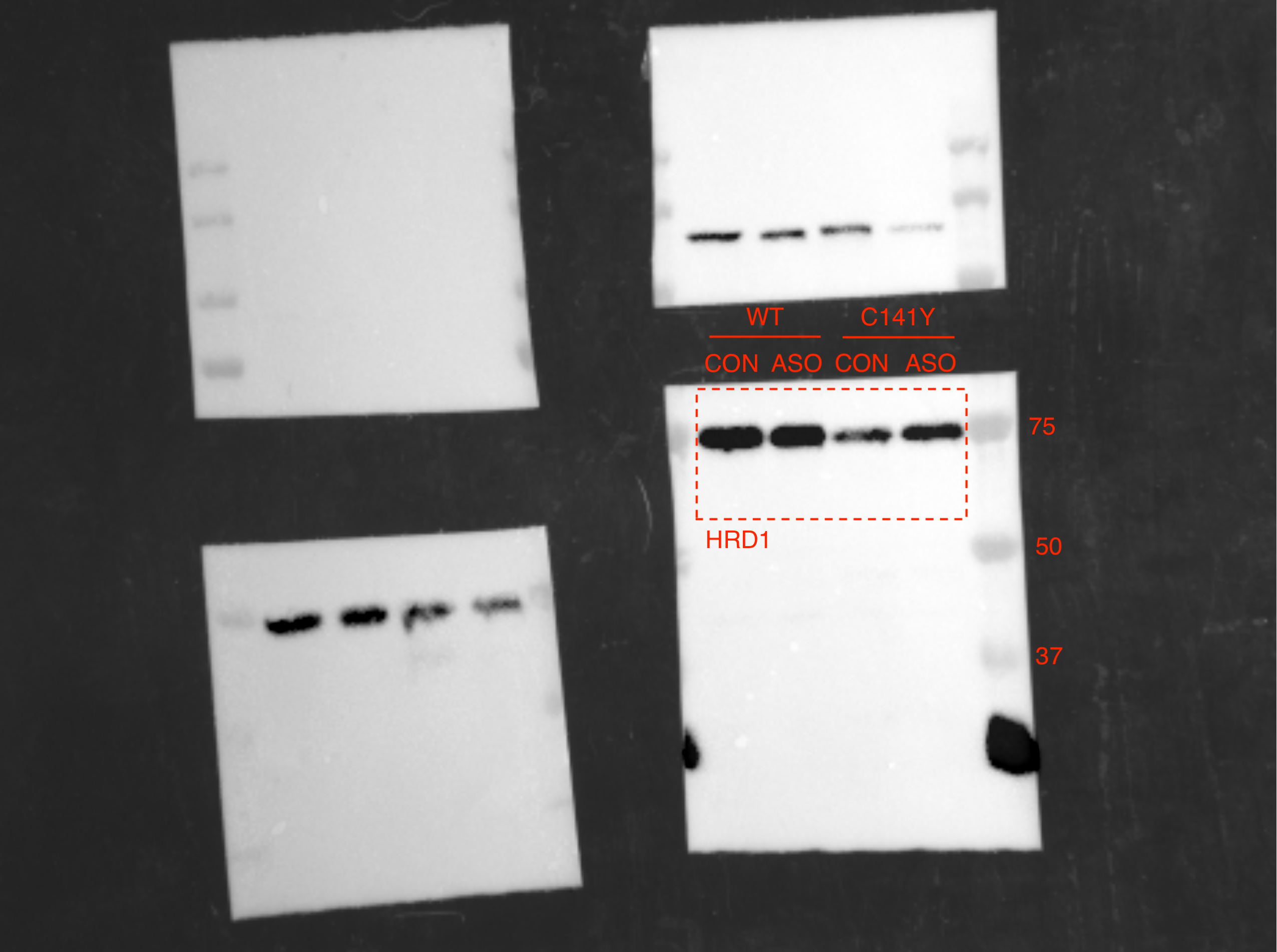

Supplement: Supplementary file 12 — Source data Fig. 7 [file 44318_2026_757_MOESM12_ESM.zip › Figure 7/Figure 7D/WB HRD1 merged with marker.tif]

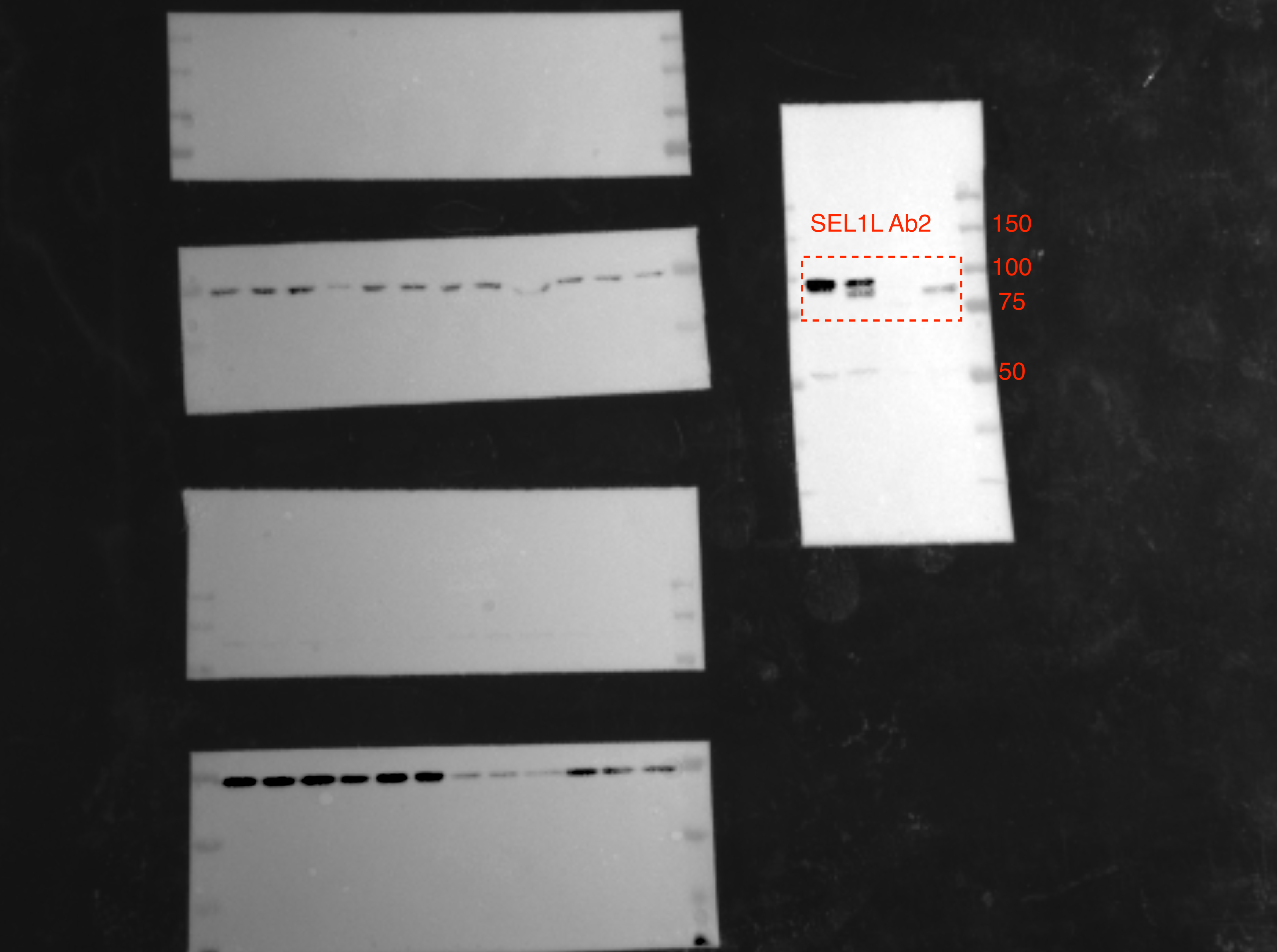

Supplement: Supplementary file 12 — Source data Fig. 7 [file 44318_2026_757_MOESM12_ESM.zip › Figure 7/Figure 7D/WB SEL1L Ab2 merged with marker.tif]

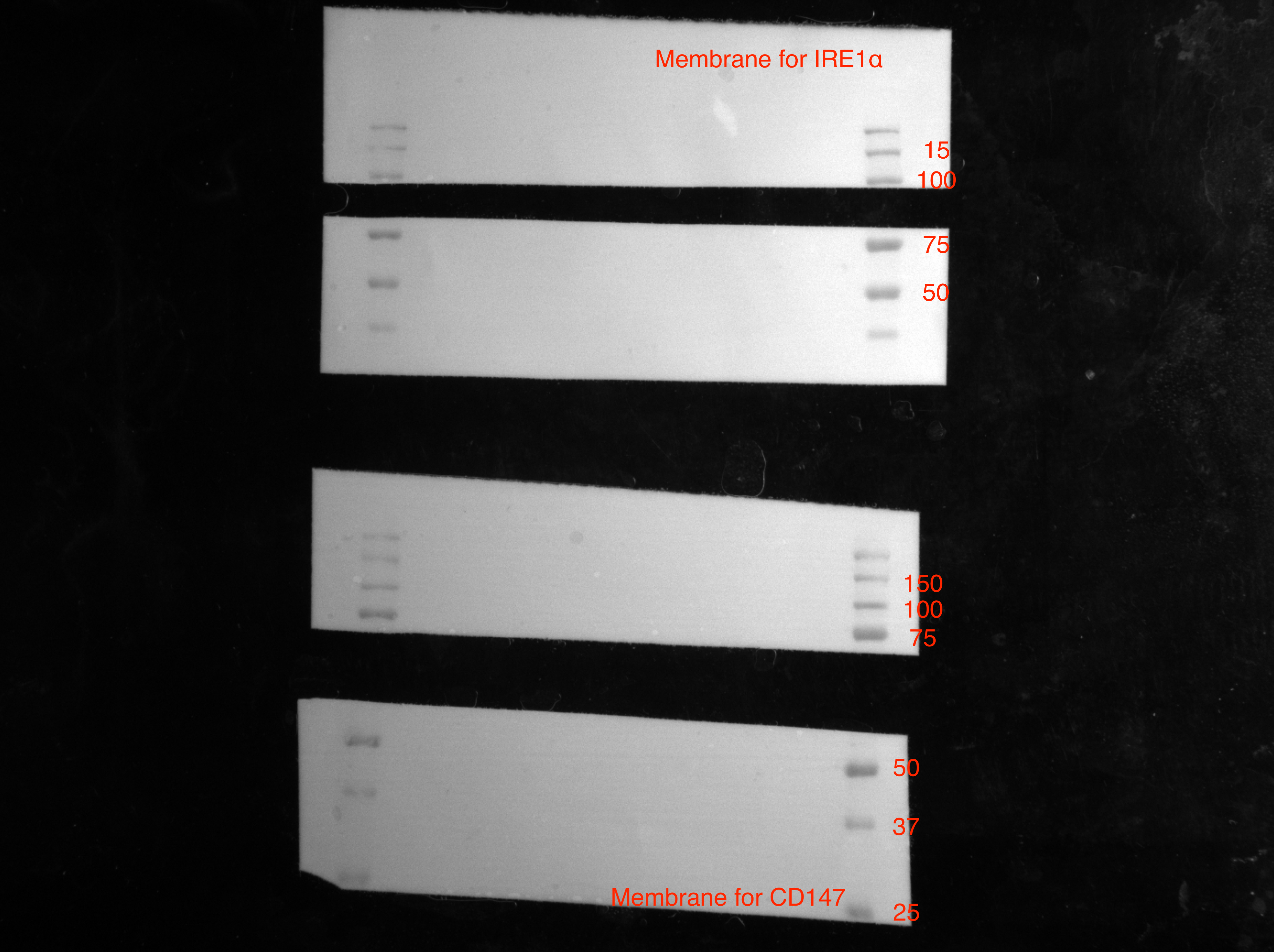

Supplement: Supplementary file 12 — Source data Fig. 7 [file 44318_2026_757_MOESM12_ESM.zip › Figure 7/Figure 7D/WB IRE1a CD147 marker_no merge.tif]

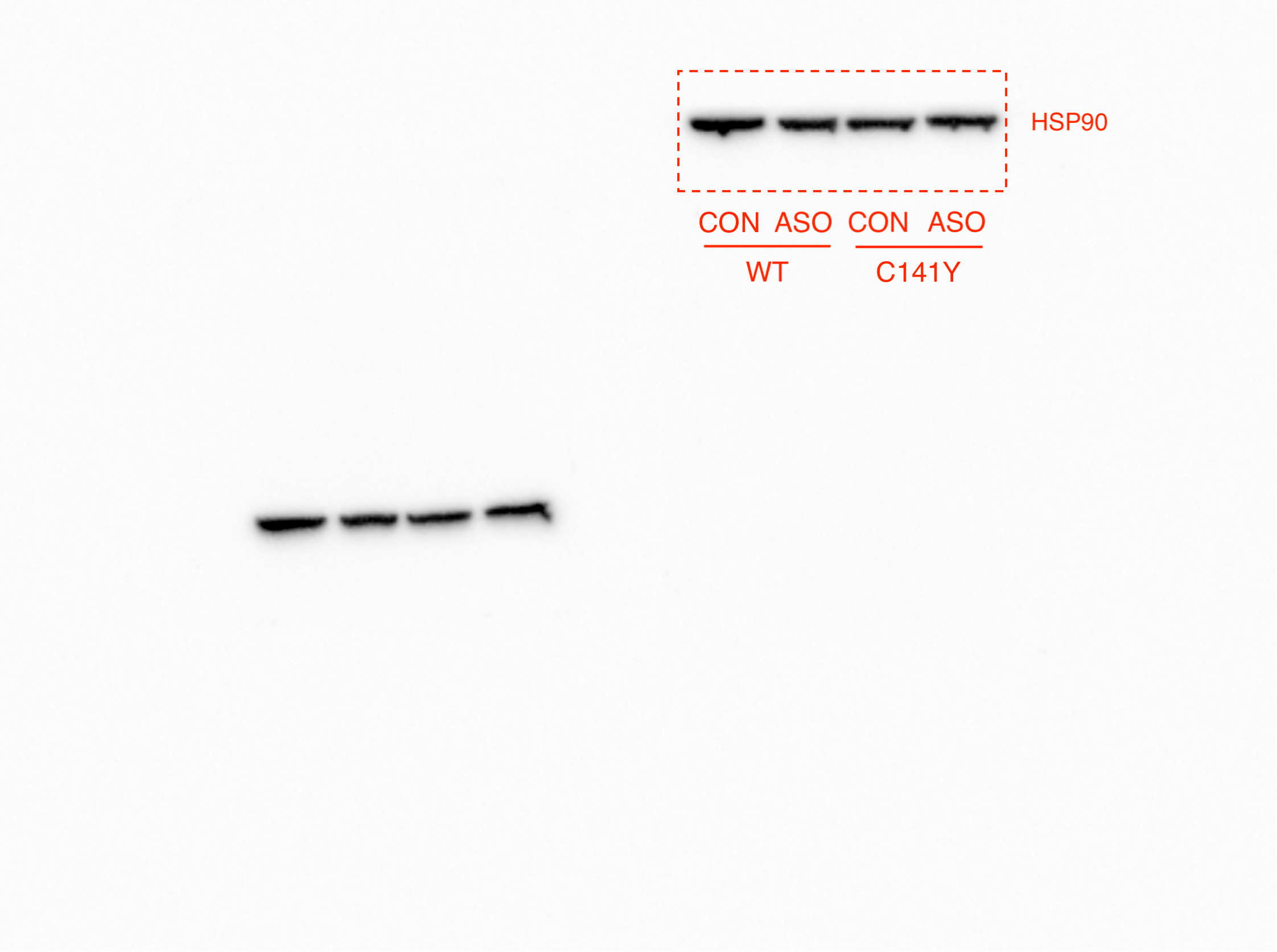

Supplement: Supplementary file 12 — Source data Fig. 7 [file 44318_2026_757_MOESM12_ESM.zip › Figure 7/Figure 7D/WB HSP90 no marker.tif]

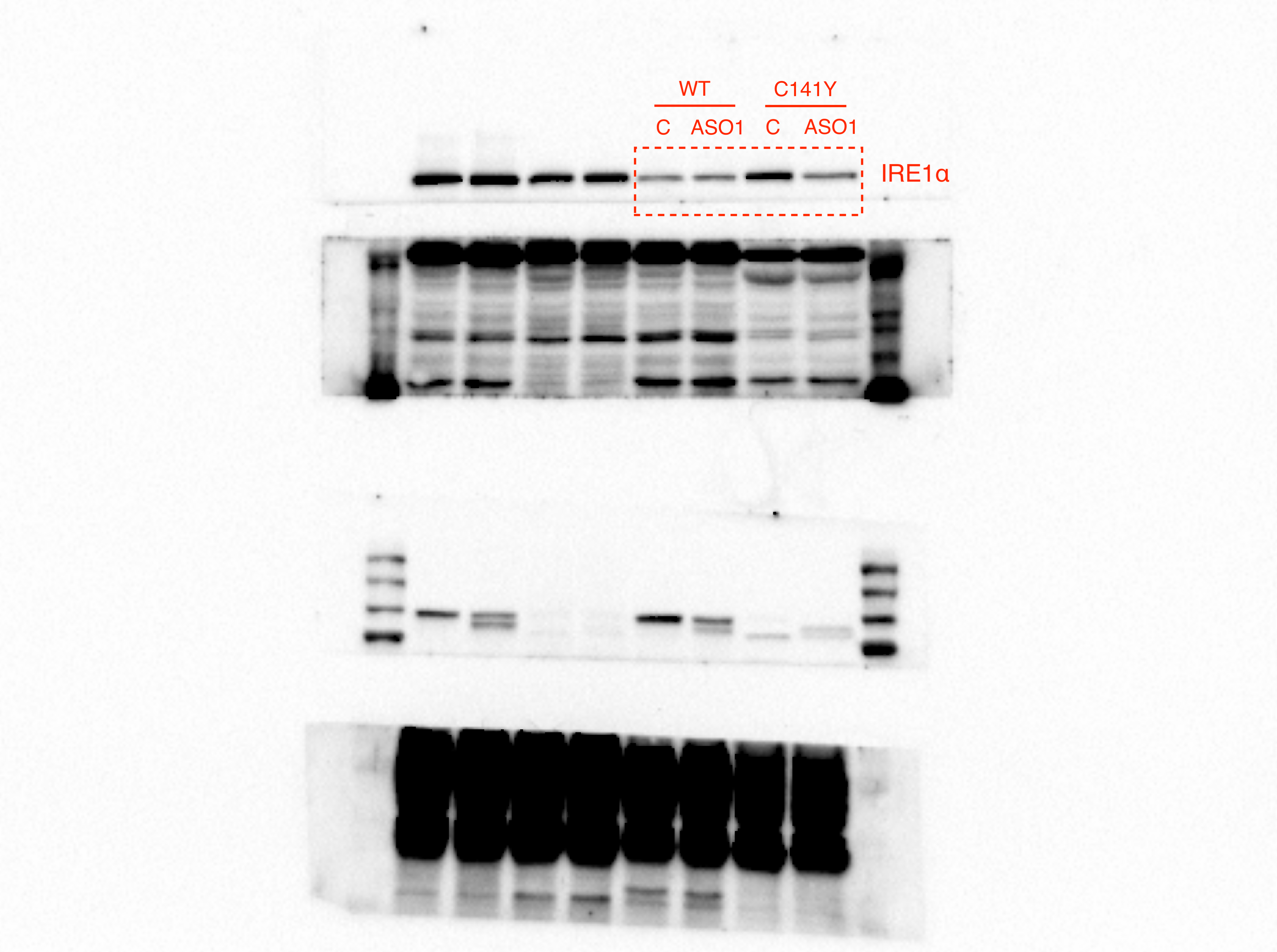

Supplement: Supplementary file 12 — Source data Fig. 7 [file 44318_2026_757_MOESM12_ESM.zip › Figure 7/Figure 7D/WB IRE1a no marker.tif]

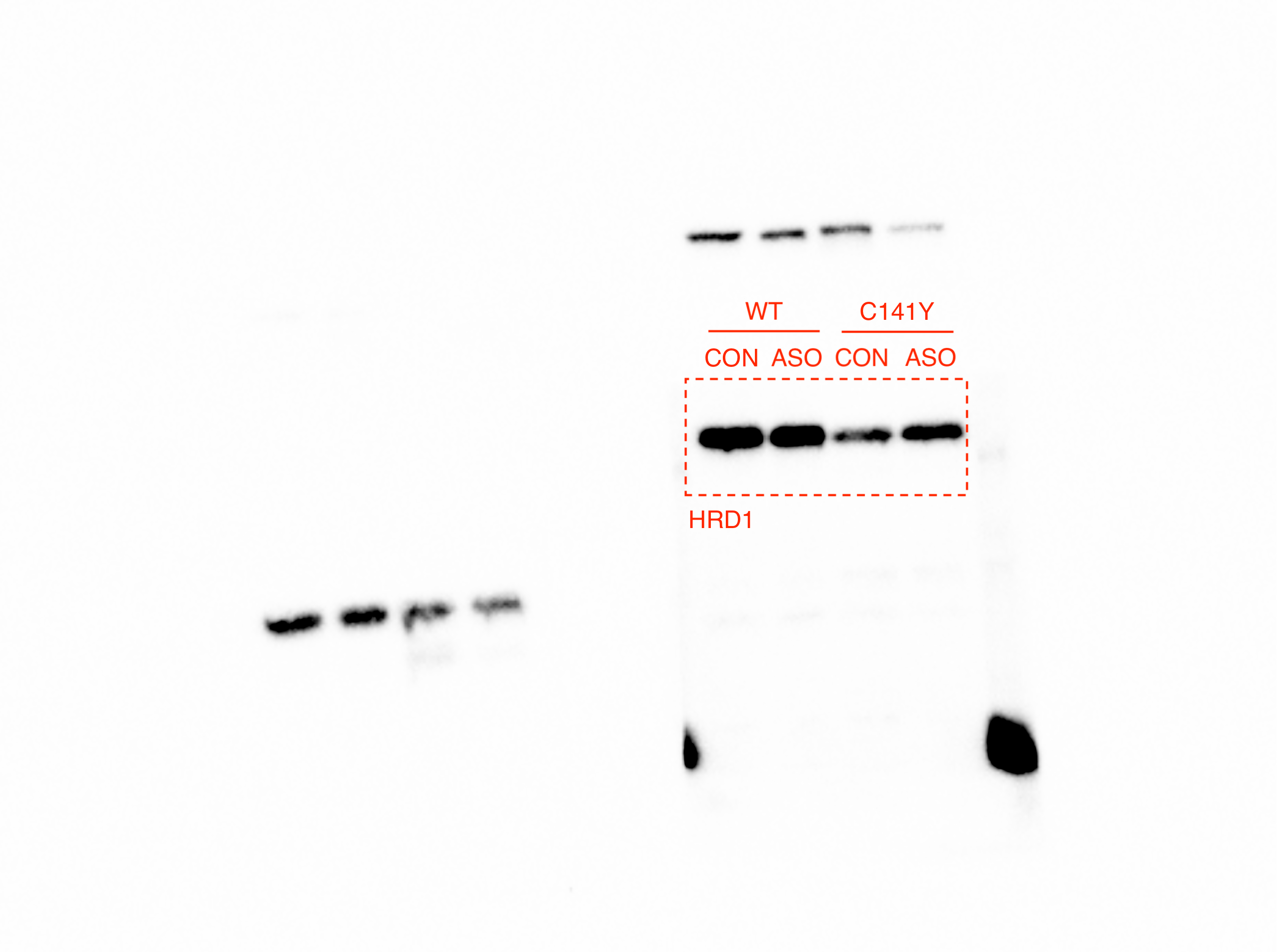

Supplement: Supplementary file 12 — Source data Fig. 7 [file 44318_2026_757_MOESM12_ESM.zip › Figure 7/Figure 7D/WB HRD1 no marker.tif]

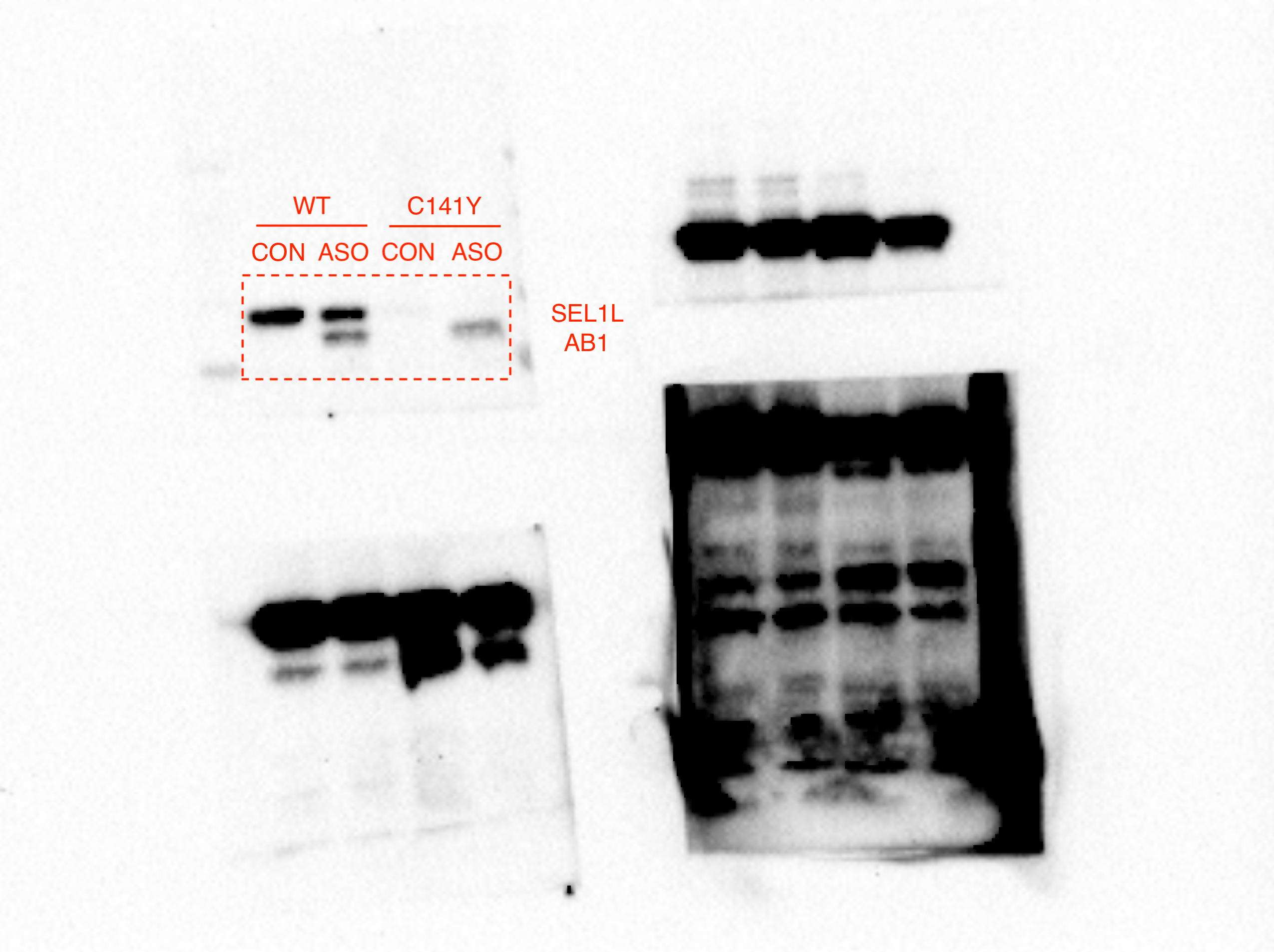

Supplement: Supplementary file 12 — Source data Fig. 7 [file 44318_2026_757_MOESM12_ESM.zip › Figure 7/Figure 7D/WB SEL1L Ab1 no marker.tif]

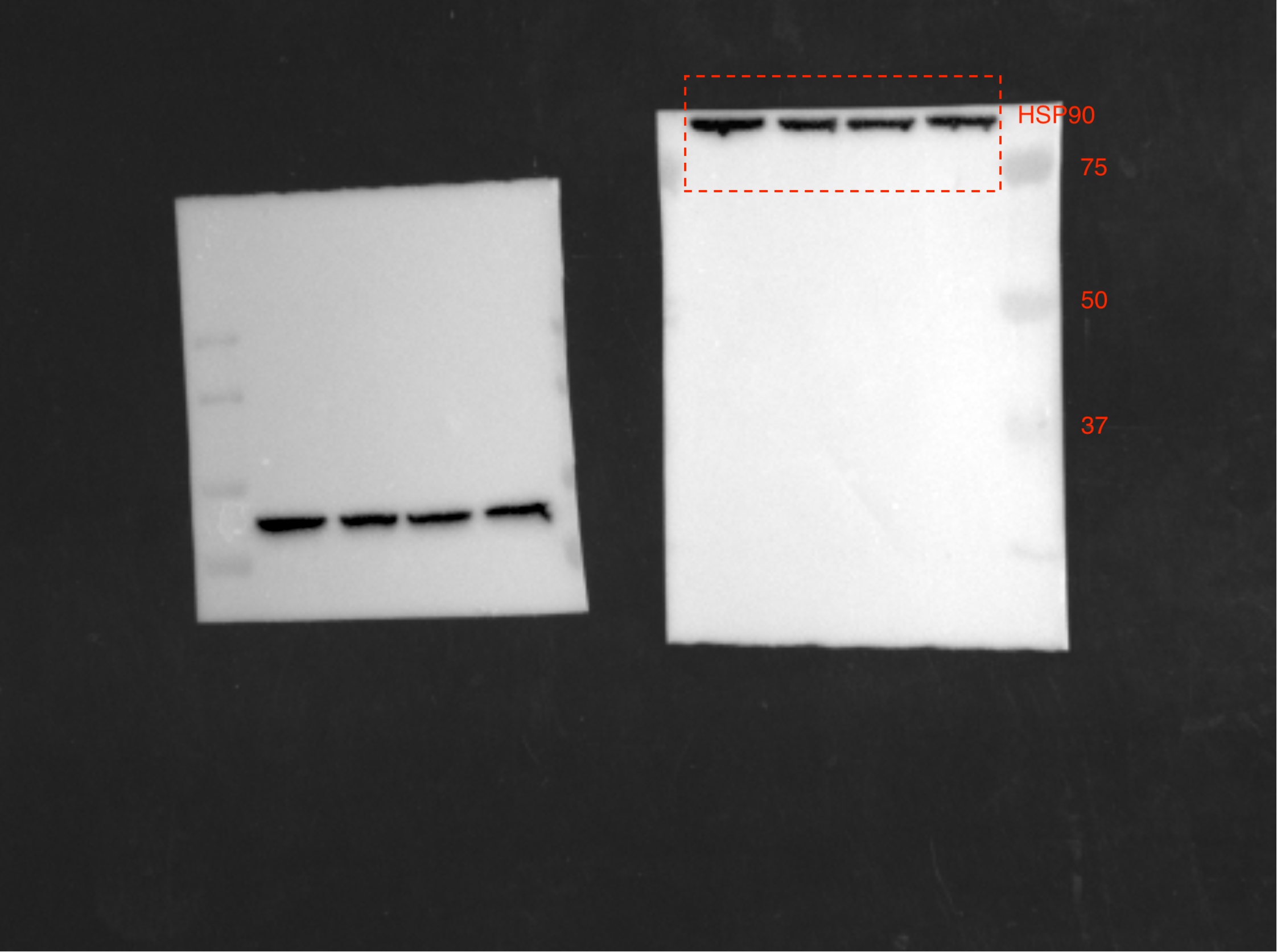

Supplement: Supplementary file 12 — Source data Fig. 7 [file 44318_2026_757_MOESM12_ESM.zip › Figure 7/Figure 7D/WB HSP90 merged with marker.tif]

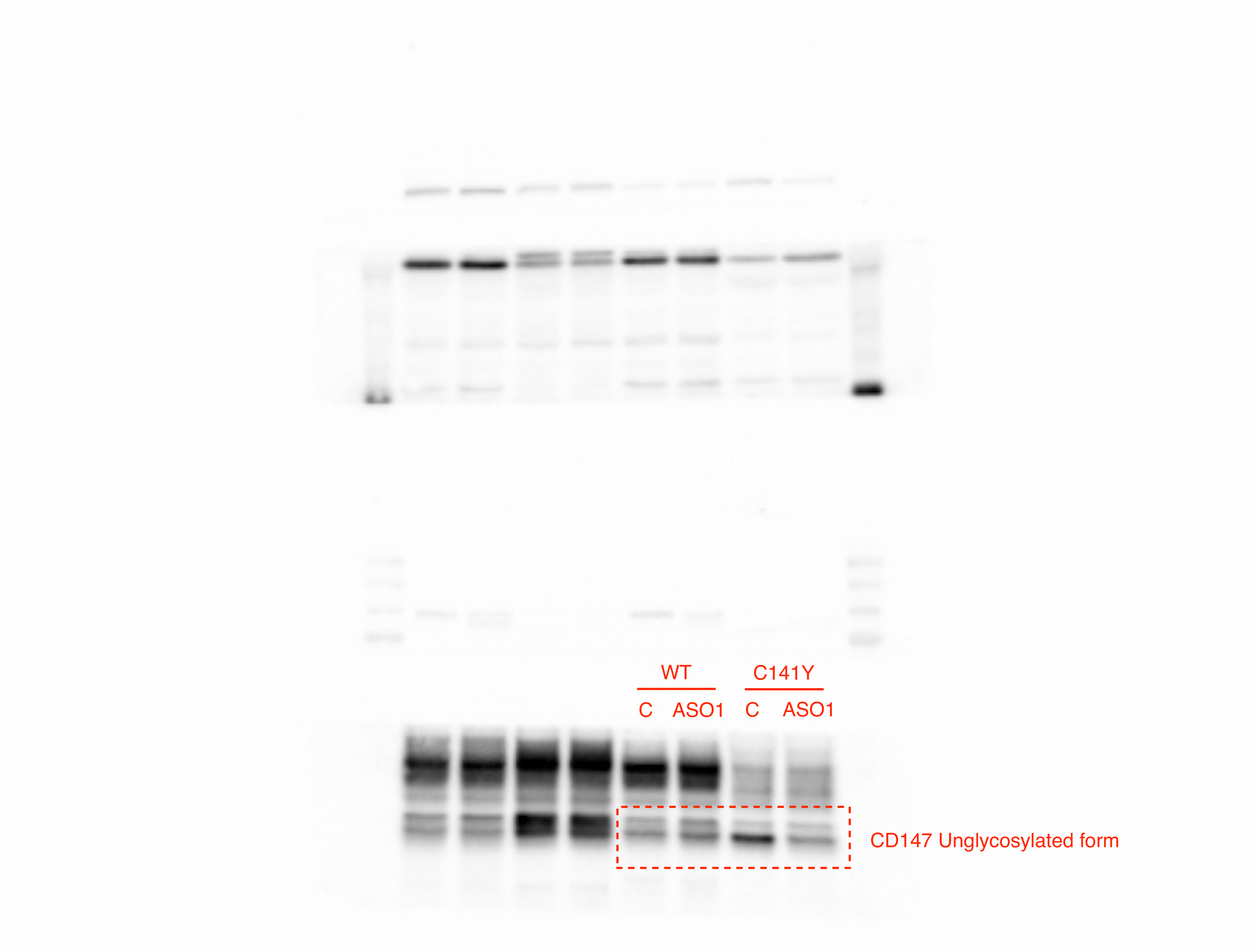

Supplement: Supplementary file 12 — Source data Fig. 7 [file 44318_2026_757_MOESM12_ESM.zip › Figure 7/Figure 7D/WB CD147 no marker.tif]

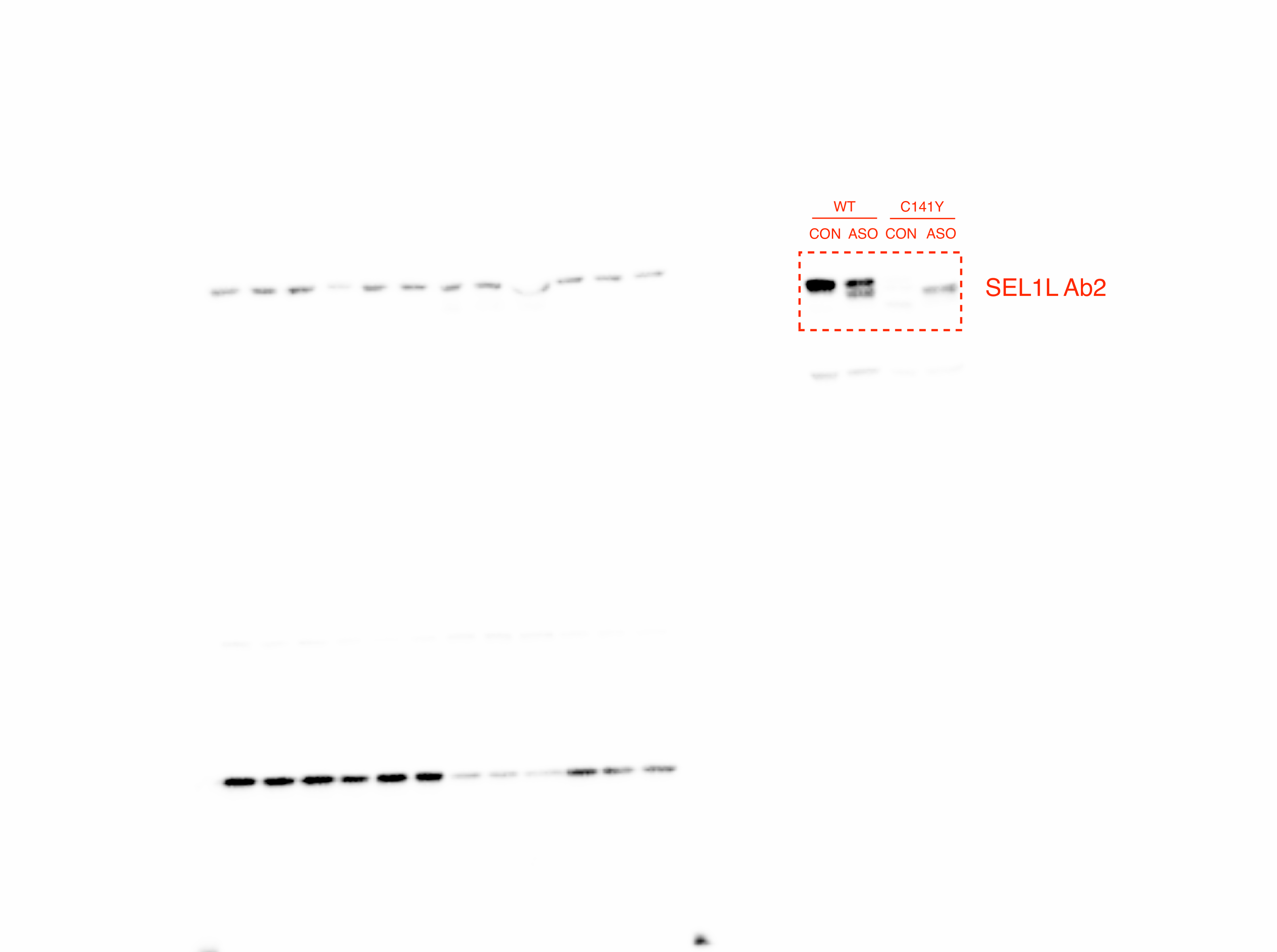

Supplement: Supplementary file 12 — Source data Fig. 7 [file 44318_2026_757_MOESM12_ESM.zip › Figure 7/Figure 7D/WB SEL1L Ab2 no marker.tif]

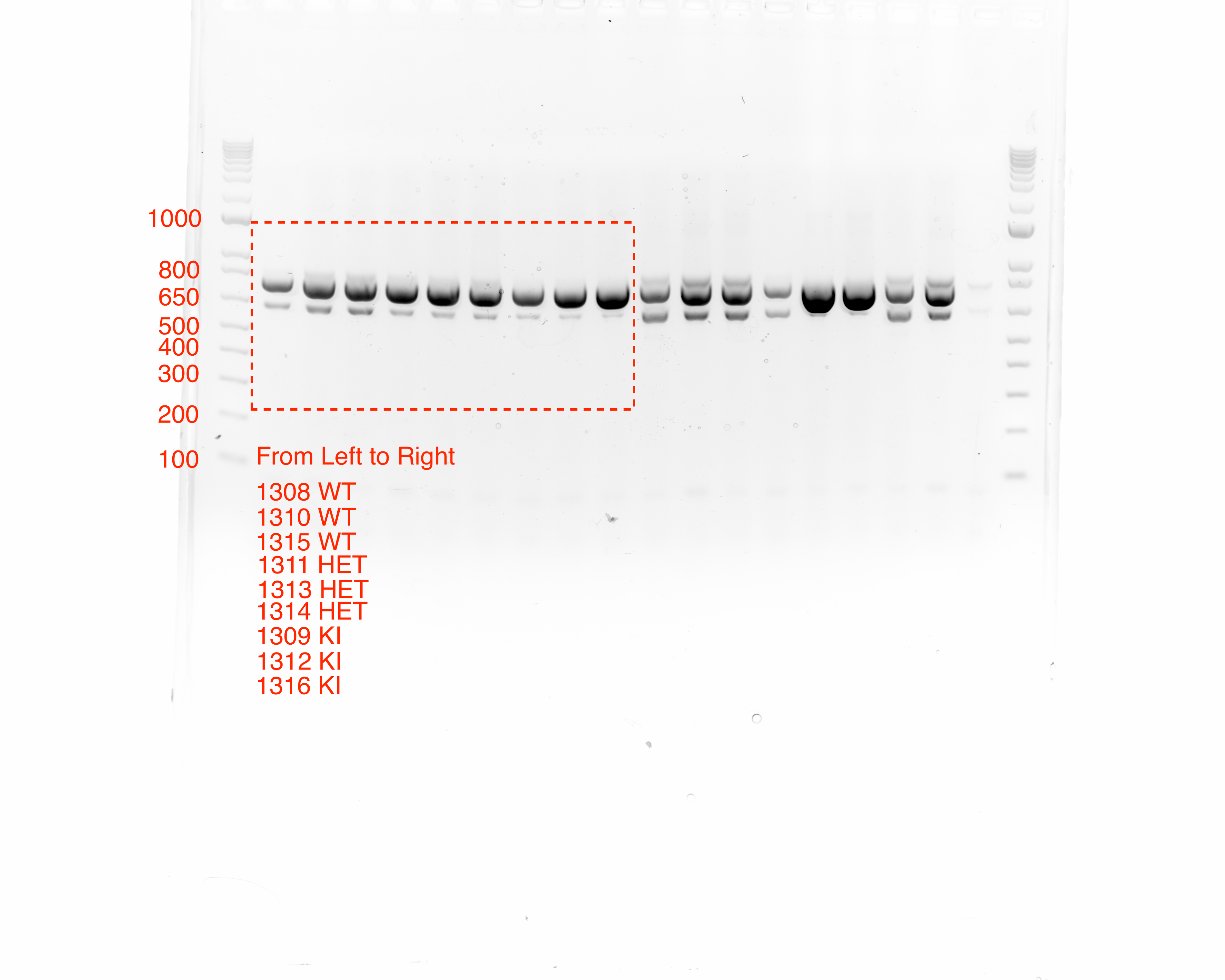

Supplement: Supplementary file 14 — Figure EV2 Source Data [file 44318_2026_757_MOESM14_ESM.zip › Figure EV2/Figure EV2D/Agarose Sel1L F3R3.tif]

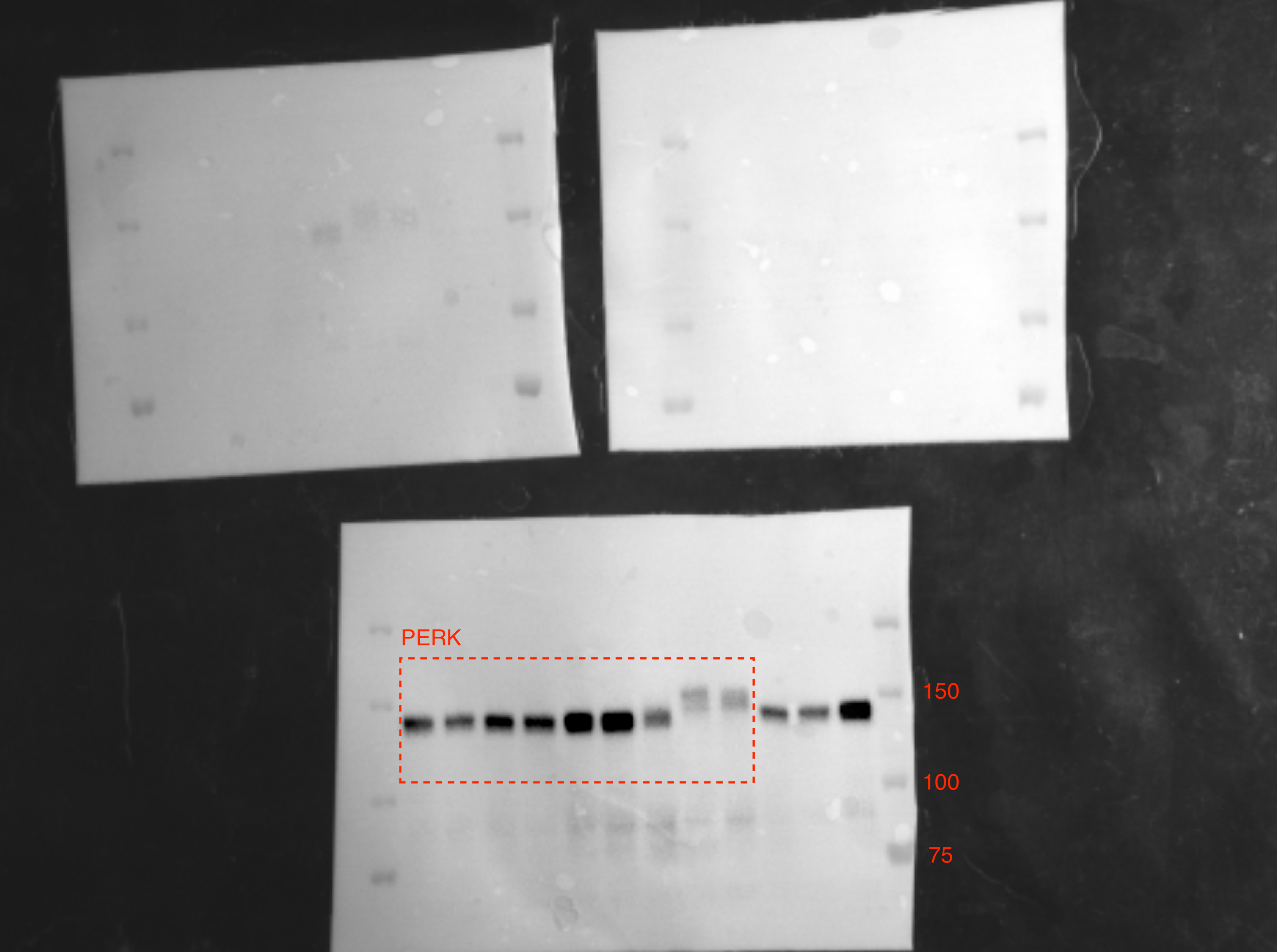

Supplement: Supplementary file 16 — Figure EV4 Source Data [file 44318_2026_757_MOESM16_ESM.zip › Figure EV4/Figure EV4C/WB PERK phosphorylation merged with marker.tif]

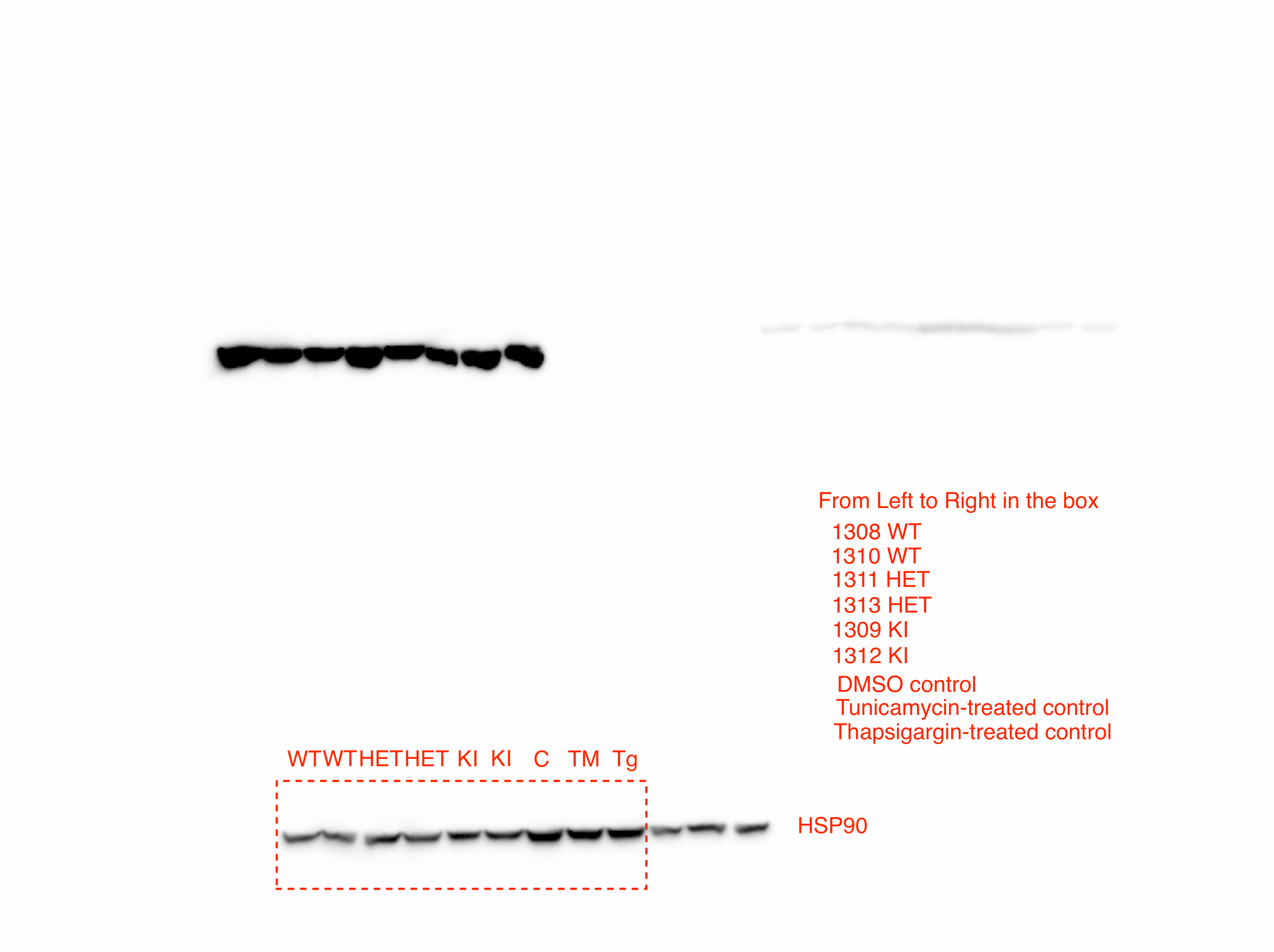

Supplement: Supplementary file 16 — Figure EV4 Source Data [file 44318_2026_757_MOESM16_ESM.zip › Figure EV4/Figure EV4C/WB HSP90 no marker.tif]

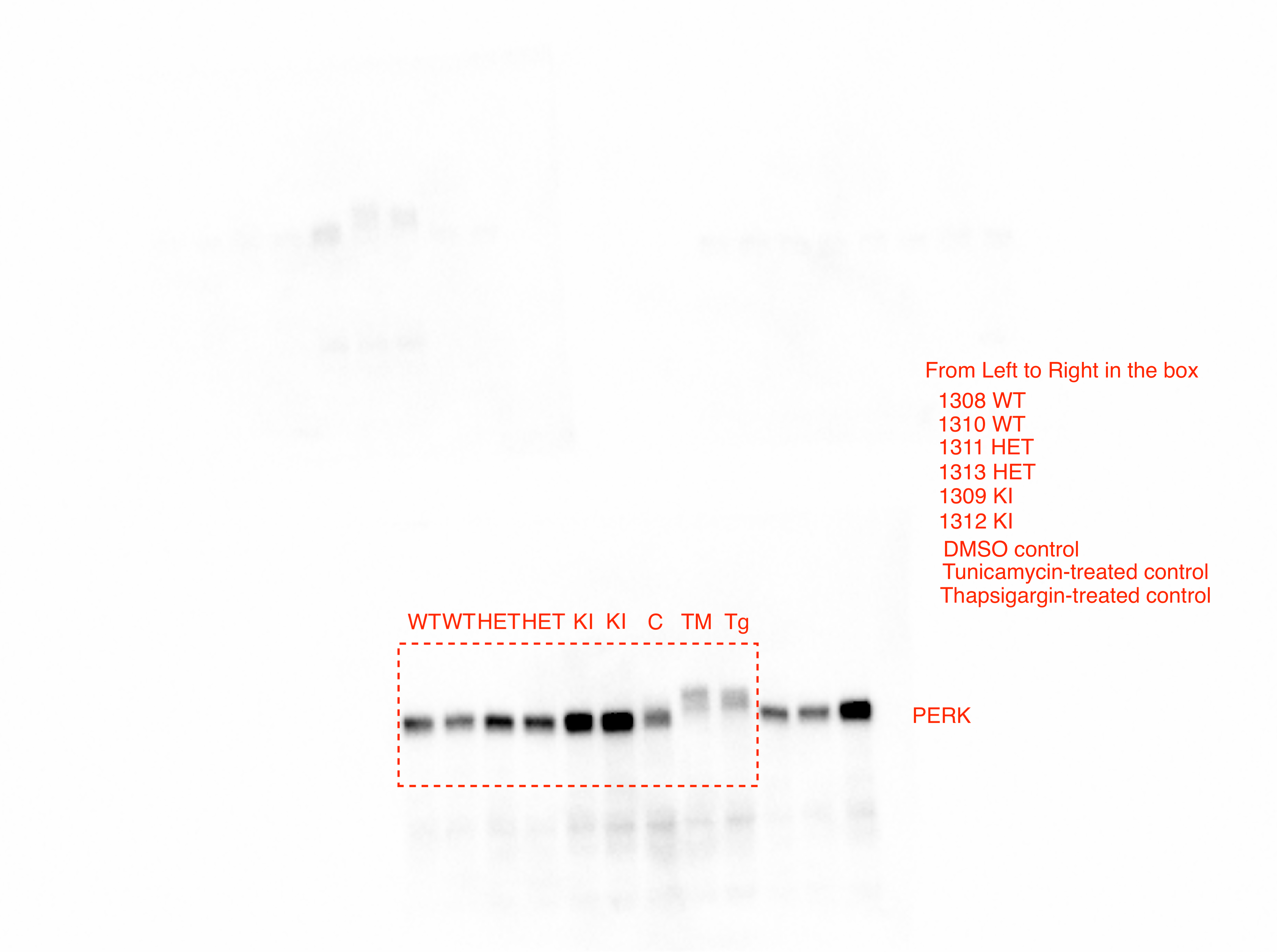

Supplement: Supplementary file 16 — Figure EV4 Source Data [file 44318_2026_757_MOESM16_ESM.zip › Figure EV4/Figure EV4C/WB PERK phosphorylation no marker.tif]

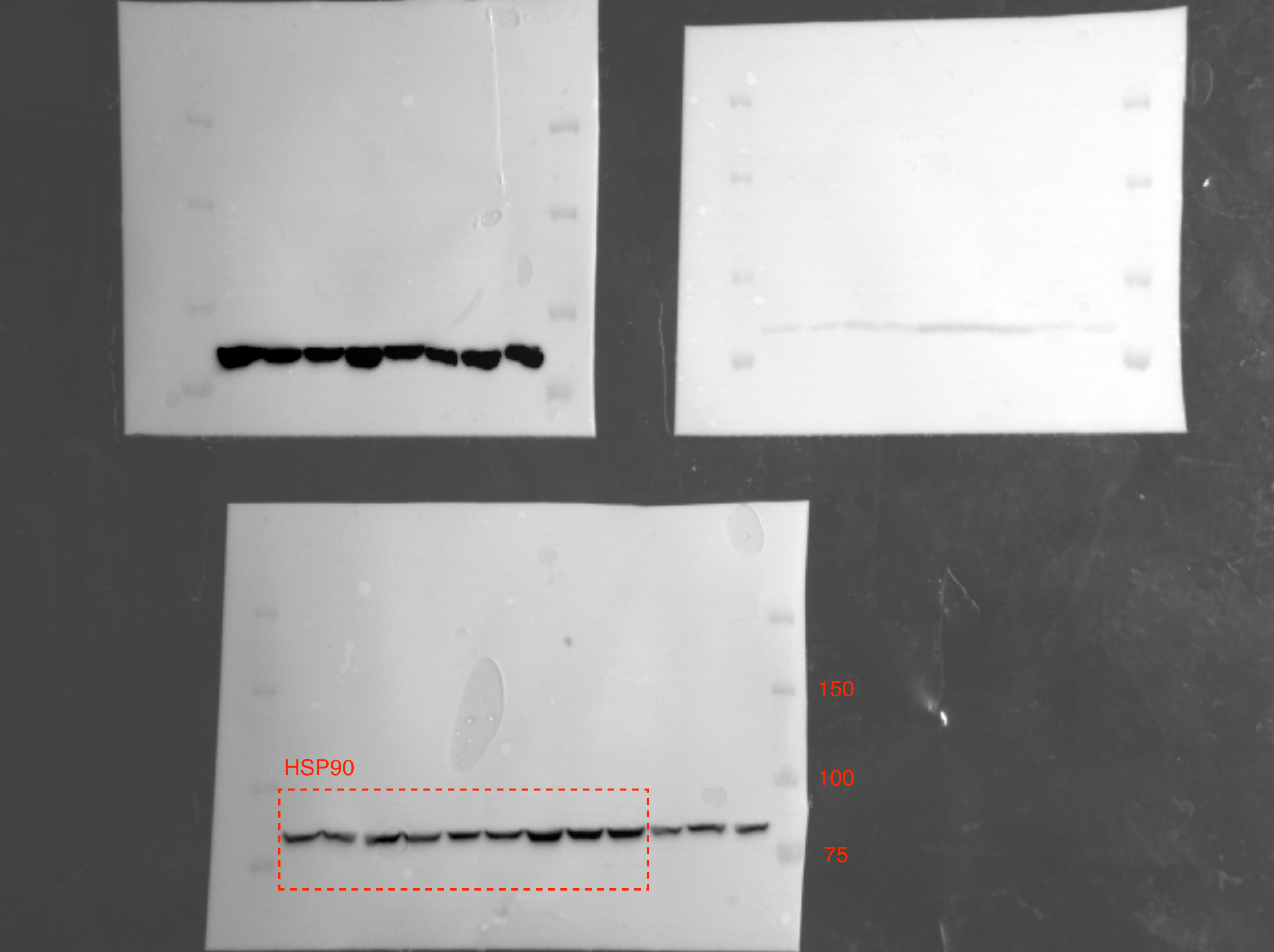

Supplement: Supplementary file 16 — Figure EV4 Source Data [file 44318_2026_757_MOESM16_ESM.zip › Figure EV4/Figure EV4C/WB HSP90 merged with marker.tif]

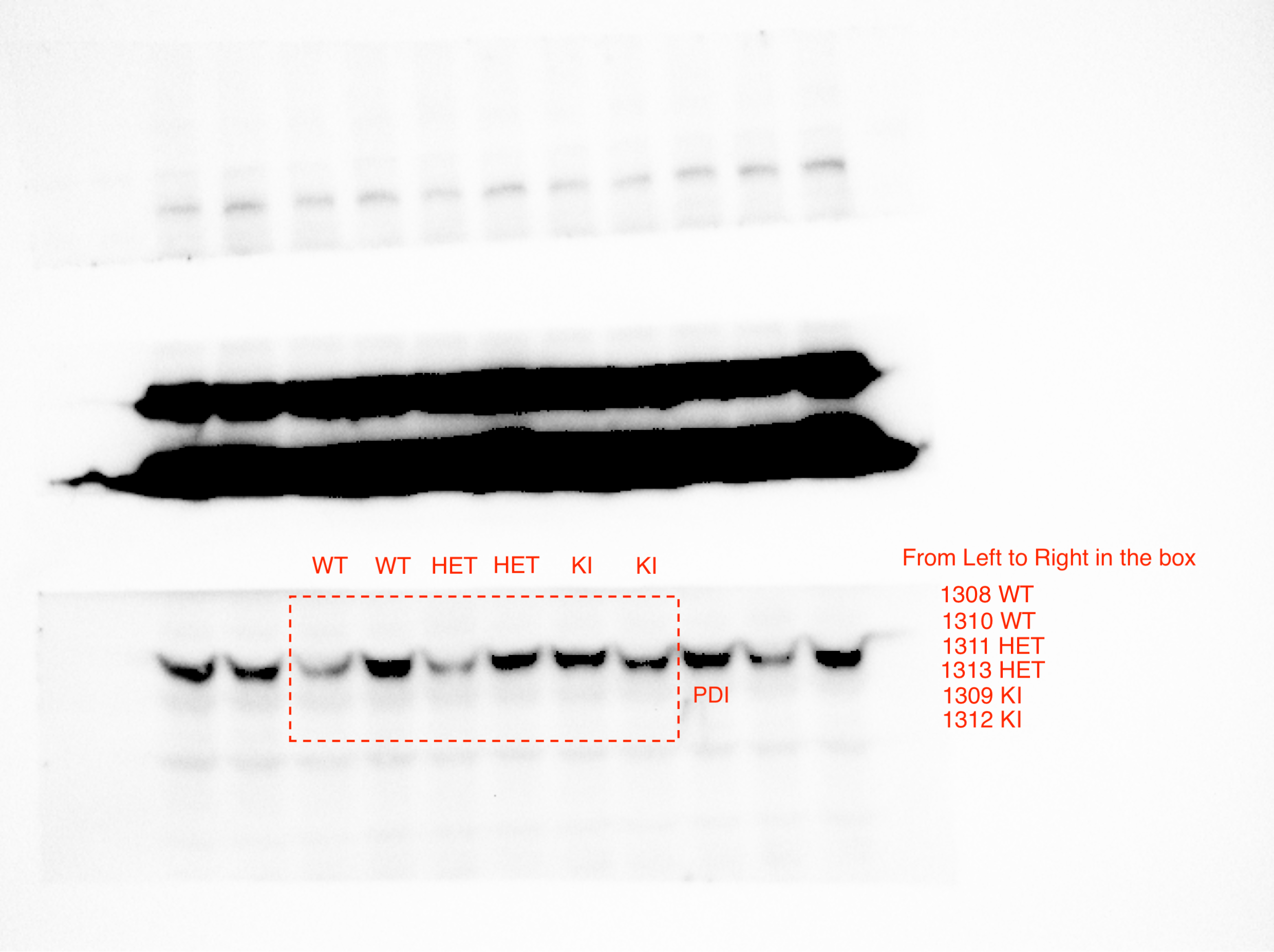

Supplement: Supplementary file 16 — Figure EV4 Source Data [file 44318_2026_757_MOESM16_ESM.zip › Figure EV4/Figure EV4B/WB PDI no marker.tif]

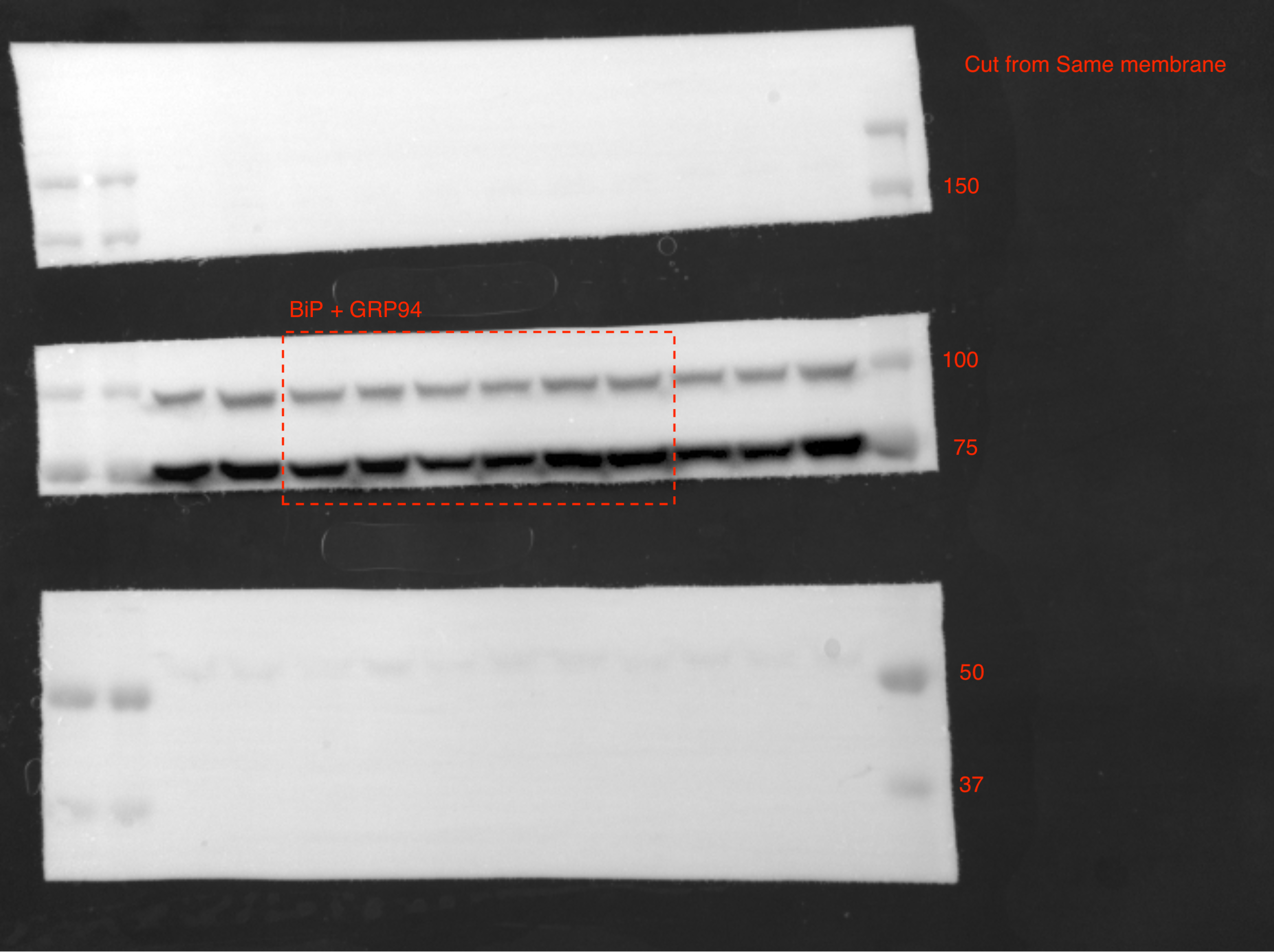

Supplement: Supplementary file 16 — Figure EV4 Source Data [file 44318_2026_757_MOESM16_ESM.zip › Figure EV4/Figure EV4B/WB BiP merged with marker.tif]

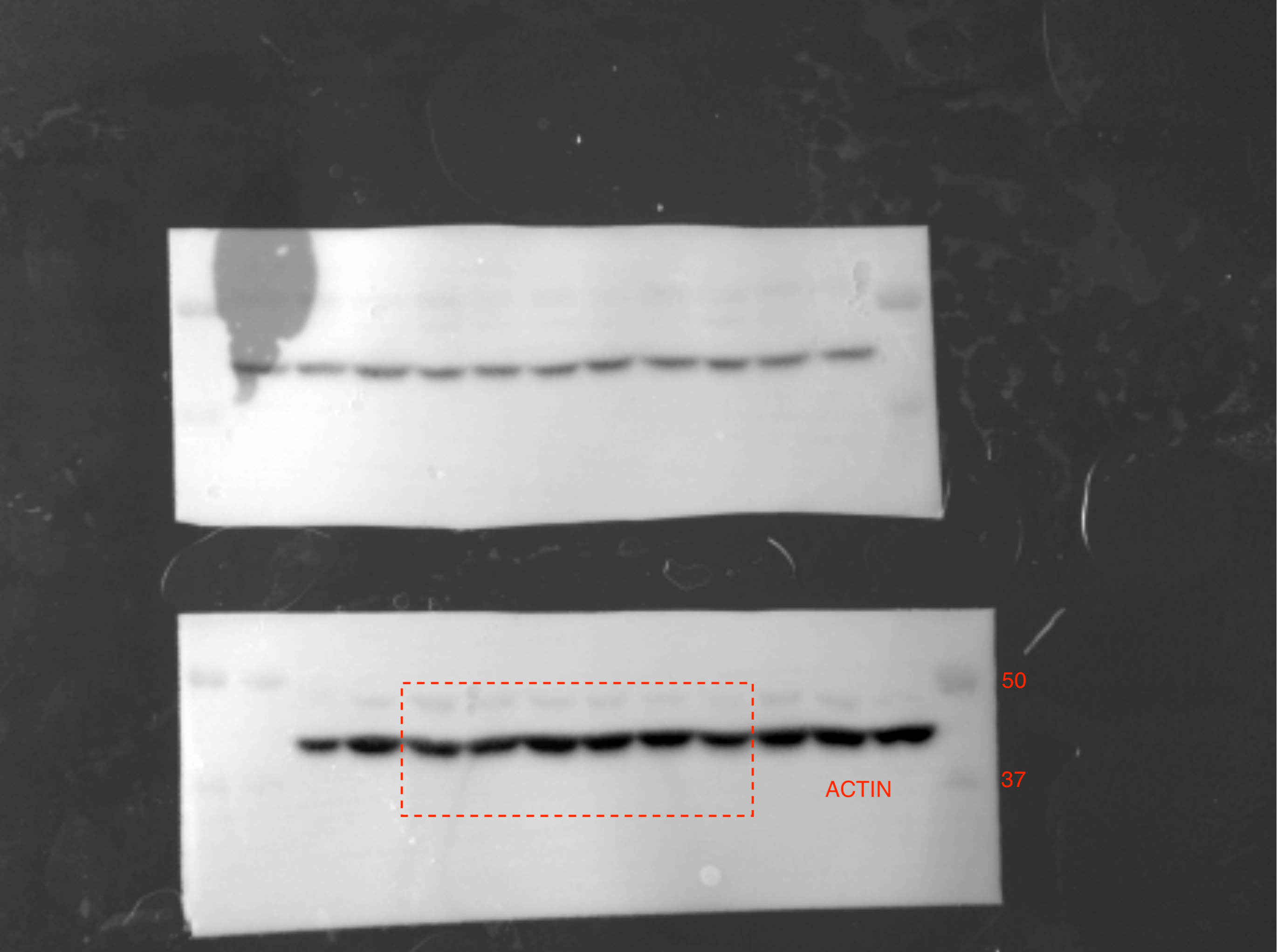

Supplement: Supplementary file 16 — Figure EV4 Source Data [file 44318_2026_757_MOESM16_ESM.zip › Figure EV4/Figure EV4B/WB ACTIN merged with marker.tif]

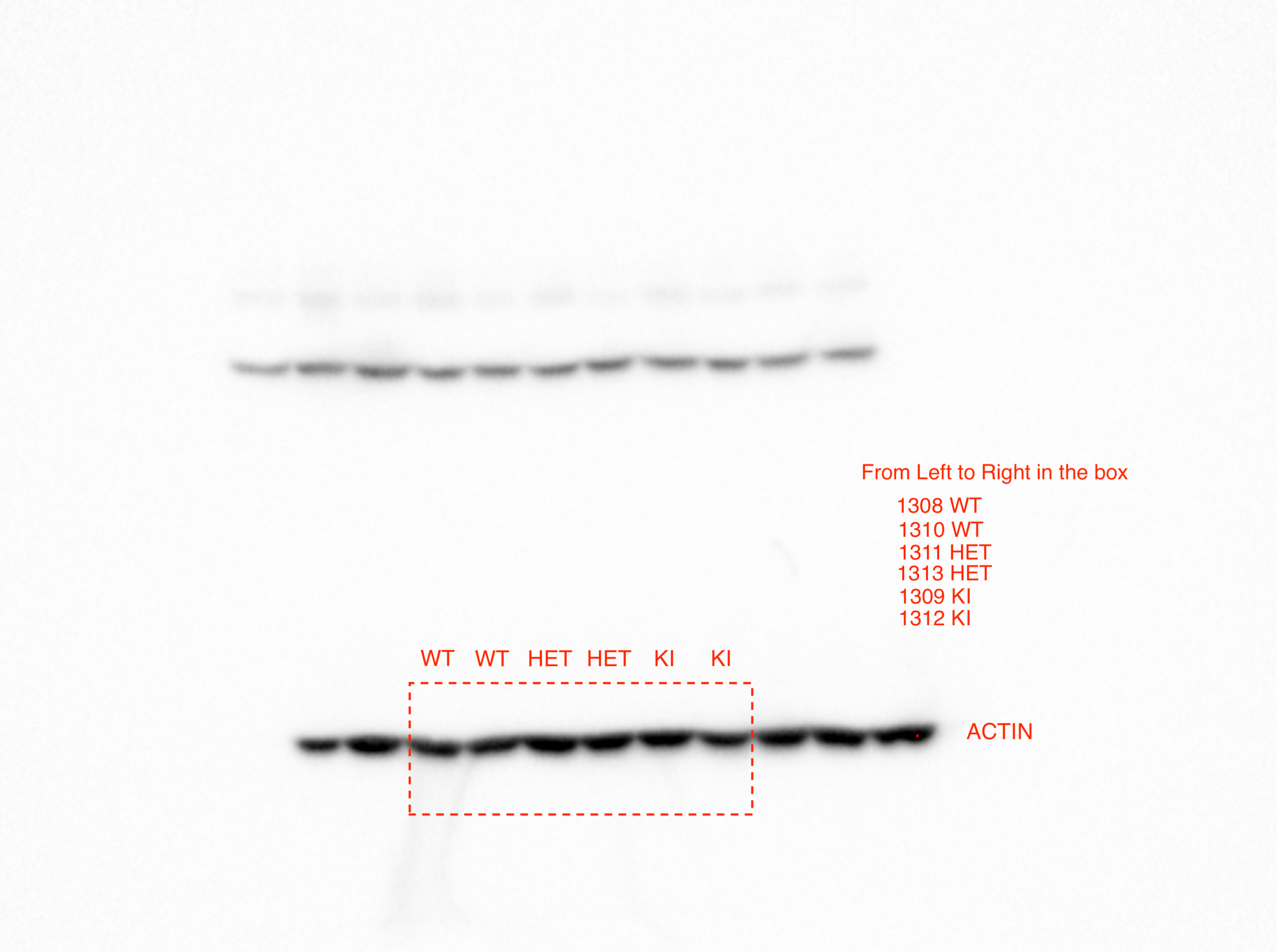

Supplement: Supplementary file 16 — Figure EV4 Source Data [file 44318_2026_757_MOESM16_ESM.zip › Figure EV4/Figure EV4B/WB ACTIN no marker.tif]

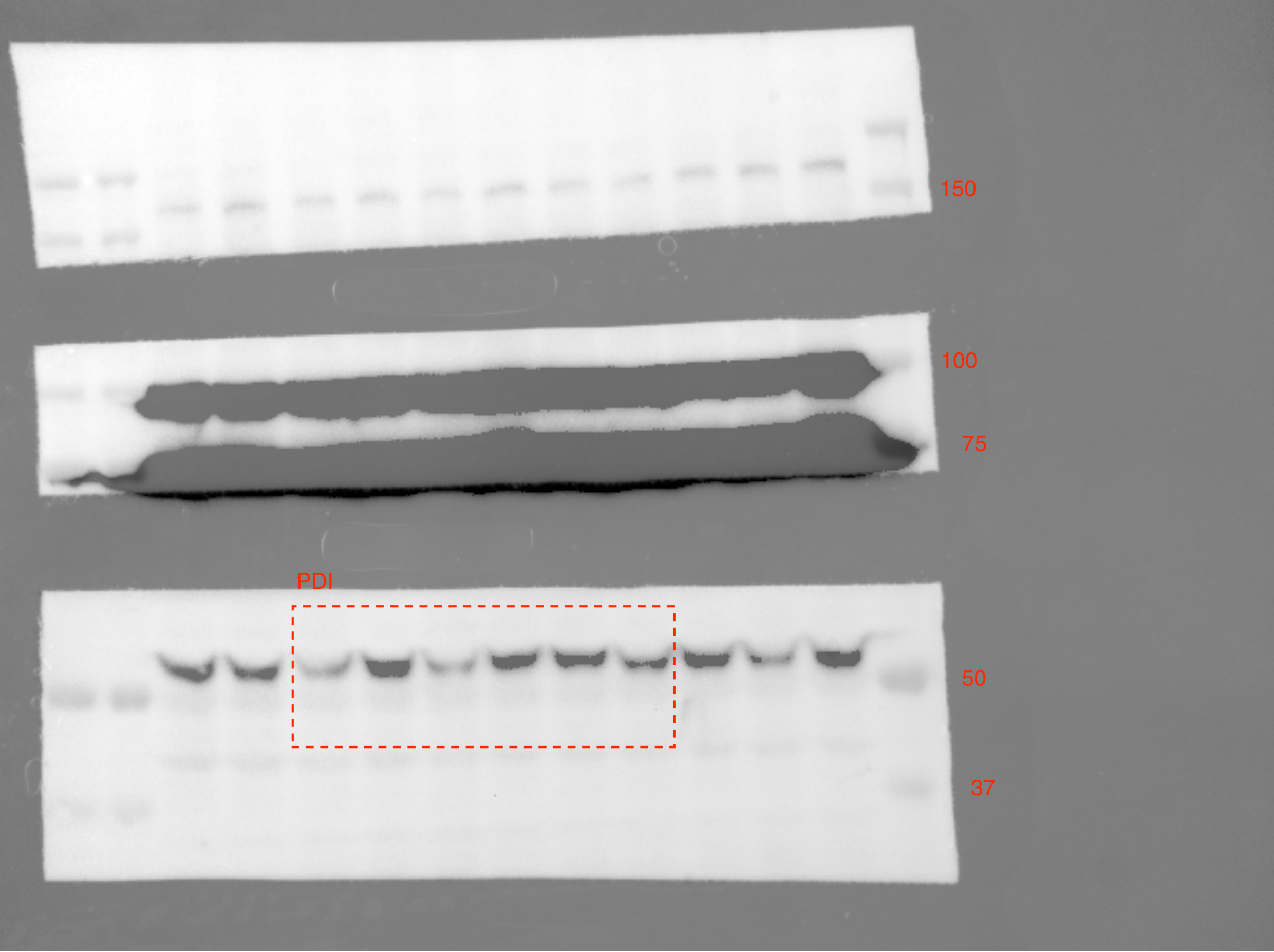

Supplement: Supplementary file 16 — Figure EV4 Source Data [file 44318_2026_757_MOESM16_ESM.zip › Figure EV4/Figure EV4B/WB PDI merged with marker.tif]

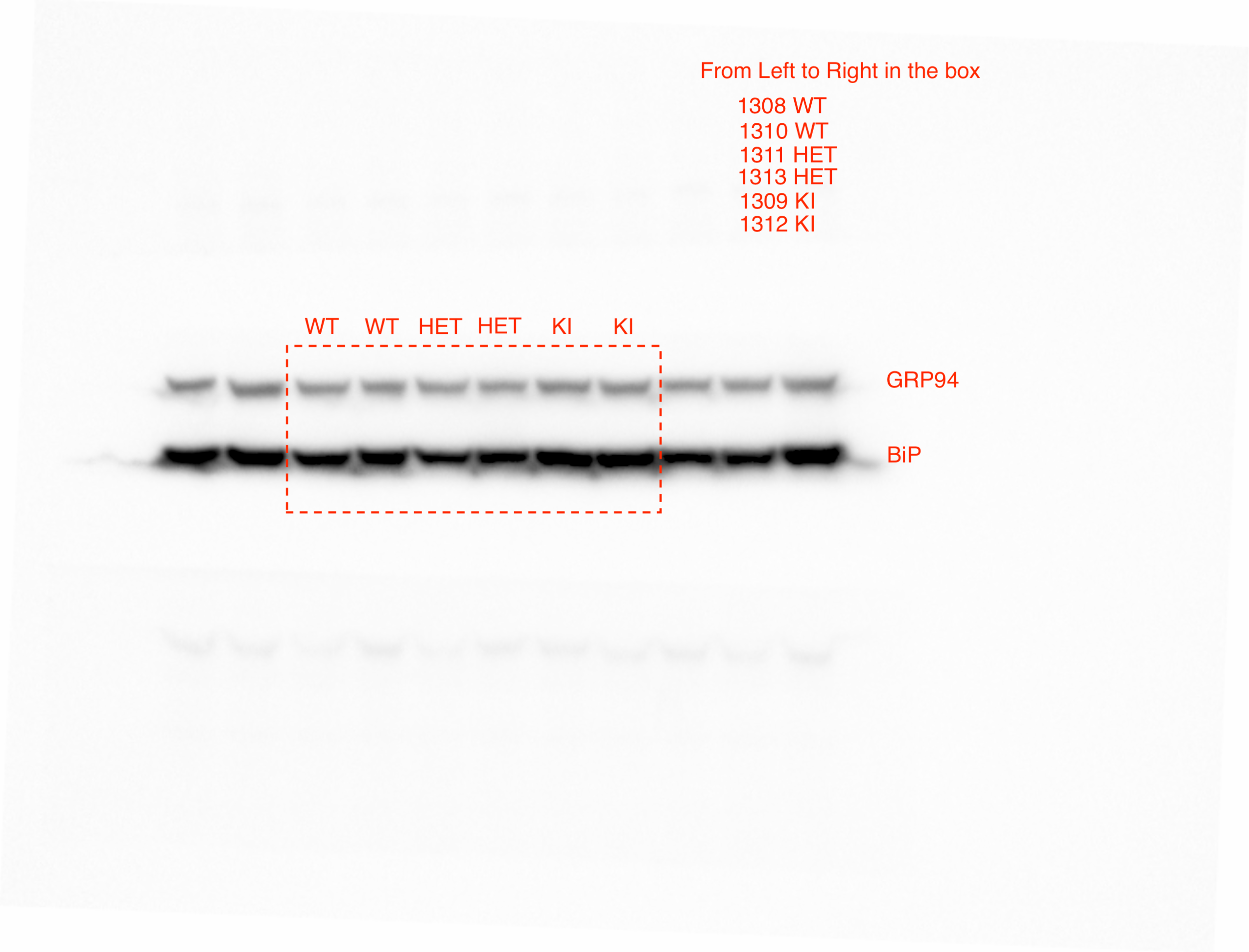

Supplement: Supplementary file 16 — Figure EV4 Source Data [file 44318_2026_757_MOESM16_ESM.zip › Figure EV4/Figure EV4B/WB BiP no marker.tif]
