# Supplementary material for: Functional rescue of a disease-linked ERAD pathway mutation via alternative splicing
Source: EMBO J. 2026 Mar 20;45(9):3230–51. doi: 10.1038/s44318-026-00757-5 (PMC13144729; doi:10.1038/s44318-026-00757-5)
Supplement: Supplementary file 18 — Expanded View Figures [file 44318_2026_757_MOESM18_ESM.pdf]

Expanded View Figures

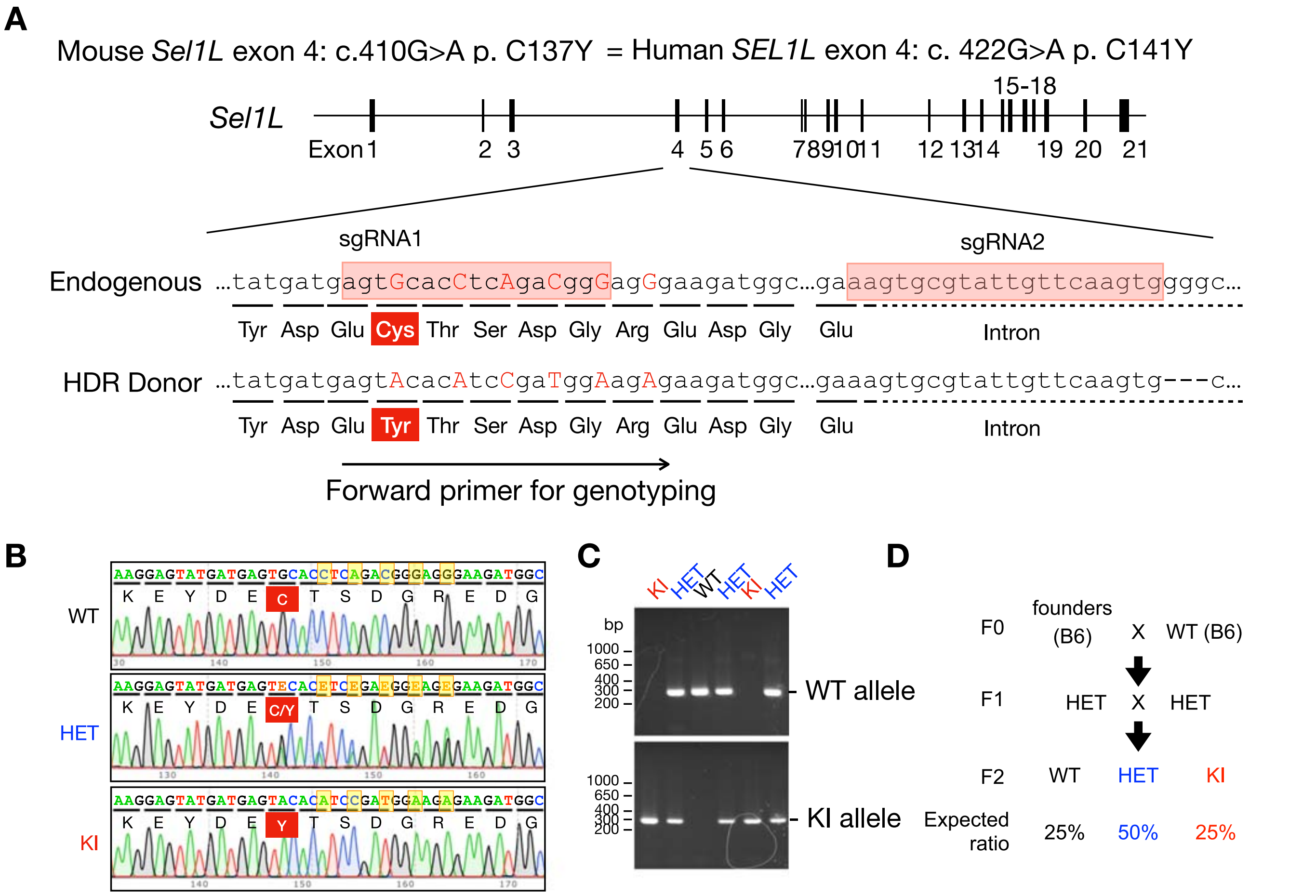

Figure EV1. The generation of SEL1L C141Y knock-in (KI) mice (related to Fig. 1).

(**A**) Diagram of sgRNAs and homology-directed repair (HDR) donor used to generate SEL1L C141Y knock-in mice via CRISPR-Cas9 technology. Silent mutations were introduced to facilitate genotyping, and the "GGG" sequence was deleted in the HDR donor (indicated as "- - -") to disrupt the PAM site. Forward primer for genotyping is indicated below the HDR donor sequence. (**B**) Sanger sequencing confirming the introduction of the SEL1L C141Y mutation along with surrounding silent mutations. E, overlapping peaks. Yellow shades, position of silent mutations. (**C**) DNA agarose gel electrophoresis for genotyping of SEL1L C141Y KI mice. (**D**) Breeding strategy for SEL1L C141Y KI mice. Three established founder lines were bred independently. Source data are available online for this figure.

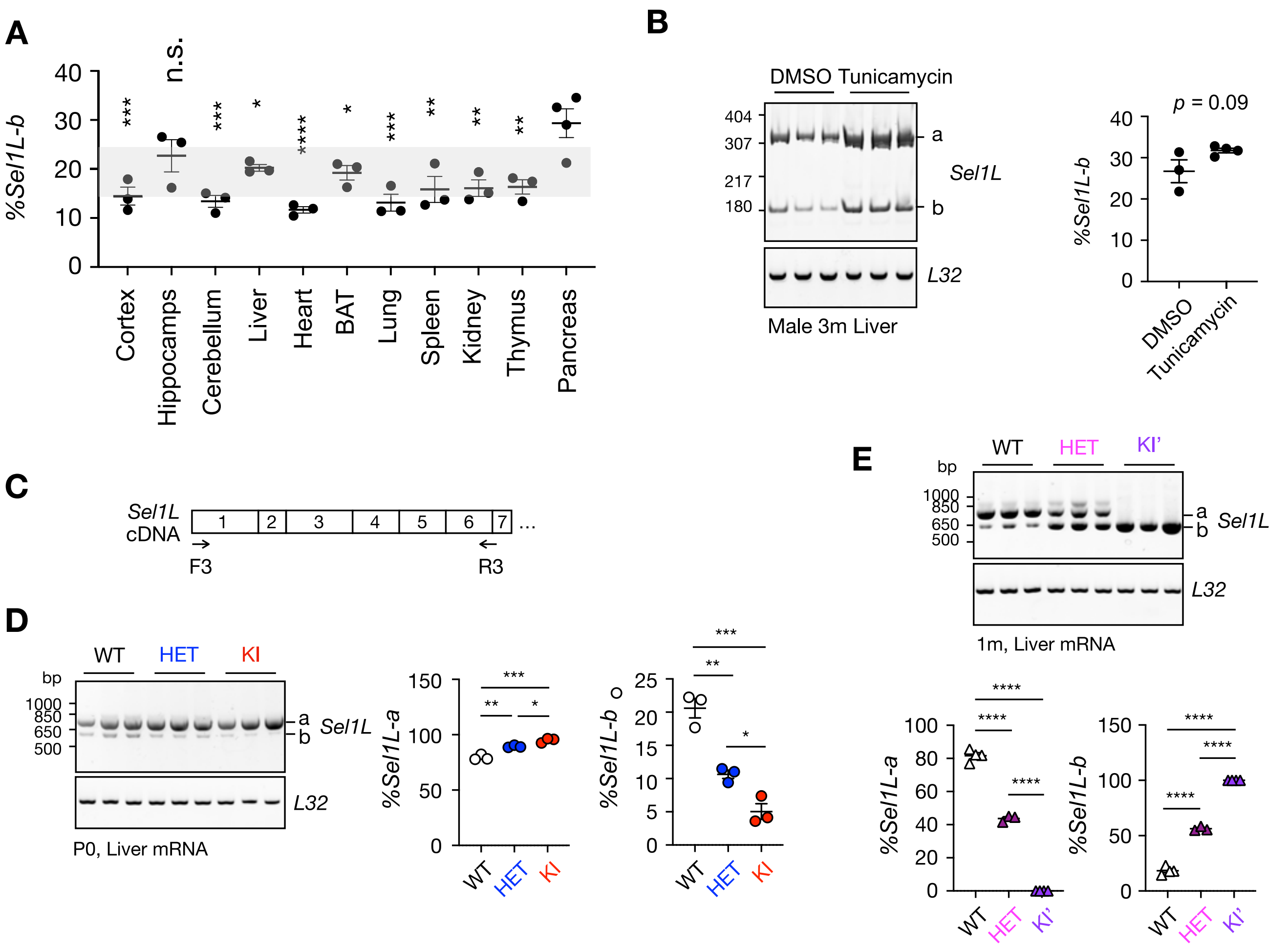

Figure EV2. Alternative splicing of *Sel1L* Exon4 under different conditions (related to Fig. 1).

(**A**) Quantification of DNA PAGE analysis of *Sel1L* exon 4 splicing in various mouse tissues shown in Fig. 1H. Statistics indicates the comparison between pancreas and other tissues. No statistically significant differences were observed between any of the other tissues. BAT, Brown adipose tissue. $n = 3$-4 mice/group. (**B**) DNA polyacrylamide gel electrophoresis (PAGE) analysis of *Sel1L* exon 4 splicing in DMSO or Tunicamycin (Tuni)-treated mouse livers, with quantification shown on the right. $n = 3$–4 mice/group. DMSO vs. Tunicamycin, $P = 0.092$. (**C**) Diagram of the primer design for (**D**, **E**). (**D**, **E**) The agarose electrophoresis of *Sel1L* isoforms in WT, HET, and KI/KI' mice, with quantification shown on the right or below. $n = 3$–4 mice/group. For (**C**), %*Sel1L-a*: WT vs. HET, $^{**}P = 0.0020$; WT vs. KI, $^{***}P = 0.0002$; HET vs. KI, $^{*}P = 0.031$. %*Sel1L-b*: WT vs. HET, $^{**}P = 0.0020$; WT vs. KI, $^{***}P = 0.0002$; HET vs. KI, $^{*}P = 0.031$. For (**D**), %*Sel1L-a*: WT vs. HET, $^{****}P < 0.0001$; WT vs. KI, $^{****}P < 0.0001$; HET vs. KI, $^{****}P < 0.0001$. %*Sel1L-b*: WT vs. HET, $^{****}P < 0.0001$; WT vs. KI, $^{****}P < 0.0001$; HET vs. KI, $^{****}P < 0.0001$. Data are represented as means ± SEM. n.s., not significant. $^{*}P < 0.05$; $^{**}P < 0.01$; $^{***}P < 0.001$, $^{****}P < 0.0001$, by one-way ANOVA followed by Tukey's post hoc test for (**A**, **D**, **E**), two-tailed t test for (**B**). Source data are available online for this figure.

 © The Author(s)

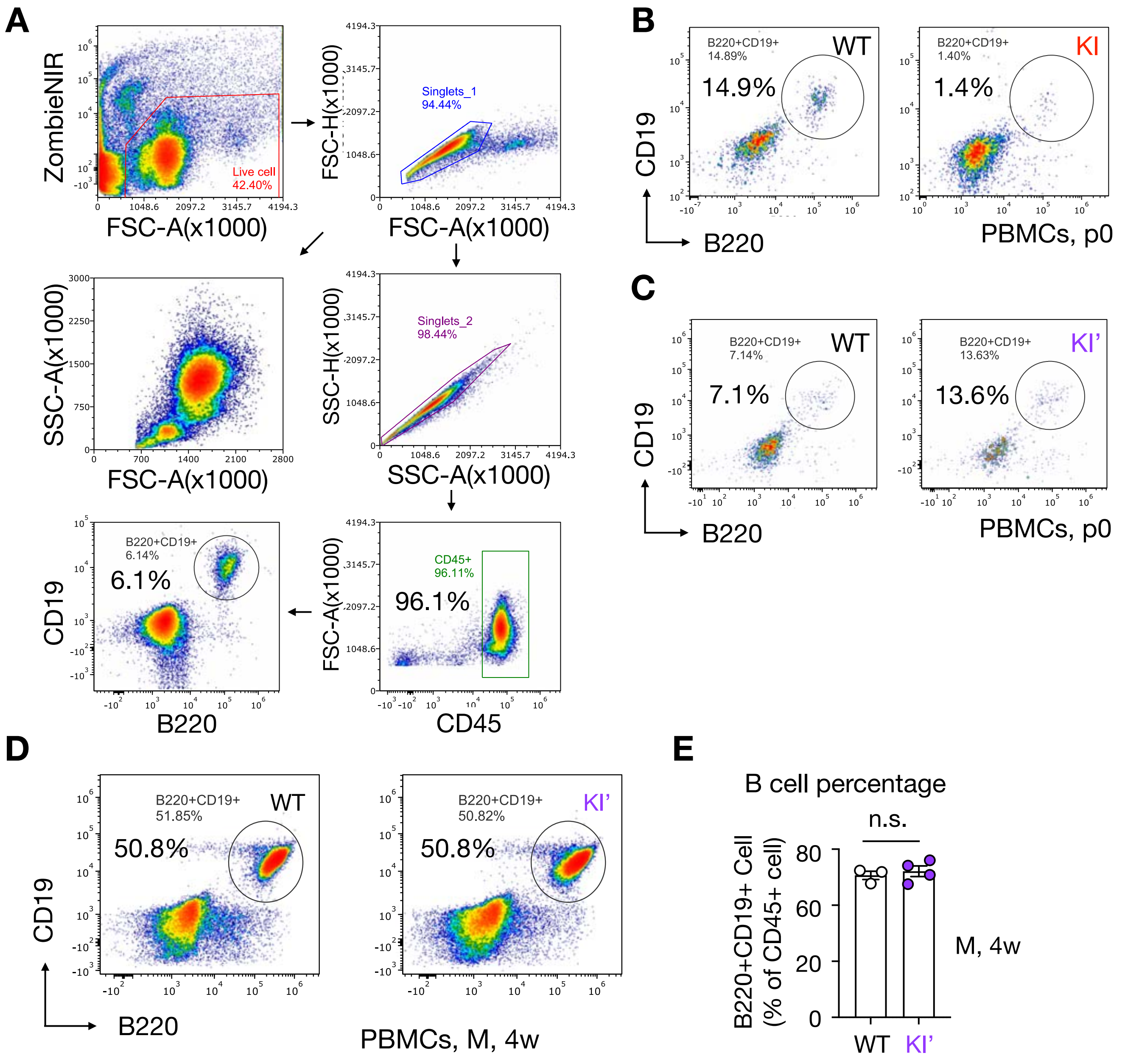

Figure EV3. B cell deficiency in the circulation of SEL1L C141Y KI mice (related to Fig. 4).

(**A**) Gating strategy for flow cytometry. Cells were first gated for live cells using Zombie NIR viability dye, followed by singlet gating to exclude doublets. Live singlet cells were then gated for CD45⁺ leukocytes, and B cells were identified as CD19⁺B220⁺ within the CD45⁺ population. (**B–D**) Representative flow cytometry plots showing CD19⁺B220⁺ B cells in PBMCs from WT and KI pups at p0 (**B**), and WT and KI′ pups at P0 (**C**) and 4 weeks of age (**D**). (**E**) Quantification of B cells as a percentage of CD45⁺ cells as shown in (**D**). $n = 3$–4 mice/group. WT vs. KI′, $P = 0.29$. Data are represented as means ± SEM. n.s., not significant using two-tailed Student's t test for (**E**). Source data are available online for this figure.

© The Author(s)

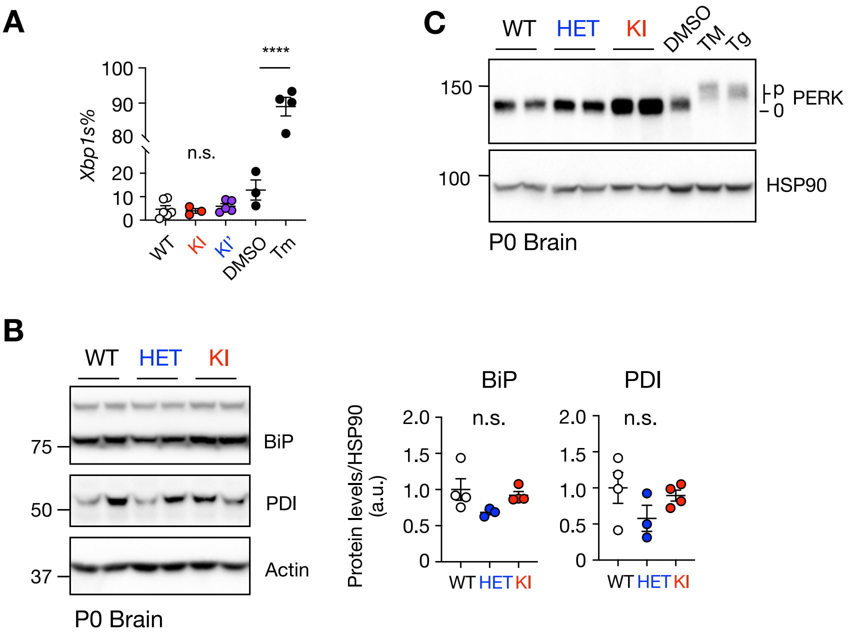

Figure EV4. SEL1L C141Y KI mice did not cause overt UPR activation (related to Fig. 5).

(**A**) Quantification of percentage of XBP1 splicing in Fig. 5G. WT vs. KI, $P > 0.99$; WT vs. KI′, $P = 0.99$, DMSO vs. Tm, ****$P < 0.0001$. (**B**) Western blot analysis of ER chaperones BiP and PDI in P0 WT, HET and KI brains, with quantification shown on the right. $n = 3$–4 mice/group. BiP: WT vs. HET, $P = 0.15$; WT vs. KI, $P = 0.84$; HET vs. KI, $P = 0.31$. PBI: WT vs. HET, $P = 0.25$; WT vs. KI, $P = 0.89$; HET vs. KI, $P = 0.44$. (**C**) Western blot analysis of PERK in p0 WT, HET and KI brains. $n = 3$–4 mice/group. p, phosphorylated. 0, unphosphorylated. Data are represented as means ± SEM. n.s. not significant; ****$P < 0.0001$ by one-way ANOVA followed by Tukey's post hoc test for (**A**, **B**). Source data are available online for this figure.

 © The Author(s)
